# Explainable Artificial Intelligence to Unveil Patterns of Antioxidant Peptides for Free Radical Regulation

**DOI:** 10.1021/acs.jcim.6c01872

**Published:** 2026-09-17

**Authors:** Maria Carolina J. A. Schneider, Bárbara Saraiva Souza, Leonardo Vasconcelos Ferreira, Marina Geisiely Damaso, André Silva Pimentel

**Affiliations:** Departamento de Química, Pontifícia Universidade Católica do Rio de Janeiro, Rio de Janeiro, RJ 22453-900 Brazil

## Abstract

Antioxidant peptides are emerging as promising therapeutic and nutraceutical agents because of their capacity to neutralize free radicals, modulate oxidative stress, and contribute to disease prevention in fields ranging from oncology to dermatology and food science. However, traditional computational approaches often fail to capture the biochemical semantics and evolutionary context of peptide sequences, limiting the predictive accuracy and mechanistic interpretability. Here, we present an explainable deep learning framework integrating evolutionary scale modeling (ESM) embeddings with a temporal convolutional network (TCN) and long short-term memory (LSTM) architecture to classify antioxidant peptides and uncover key sequence determinants of activity. ESM embeddings provide rich evolutionary and structural priors, enabling the model to capture both local *k*-mer motifs and global sequence dependencies. To ensure transparency, we employed a multimethod interpretability strategy using Anchor, LIME, and SHAP, which identified robust, potentially biologically meaningful hypotheses containing residues rich in cysteine (C), arginine (R), lysine (K), histidine (H), aspartic acid (D), glutamic acid (E), threonine (T), glutamine (Q), proline (P), methionine (M), tyrosine (Y), and tryptophan (W) as central to antioxidant activity. Anchor offered deterministic, high-precision motif rules, while LIME and SHAP highlighted redundant and context-dependent contributions, together forming a layered interpretability framework. This study not only achieves state-of-the-art classification performance but also bridges black-box modeling and biochemical understanding, offering a generalizable, hypothesis-generating platform for rational peptide design and discovery that is important in human health.

## Graphical abstract

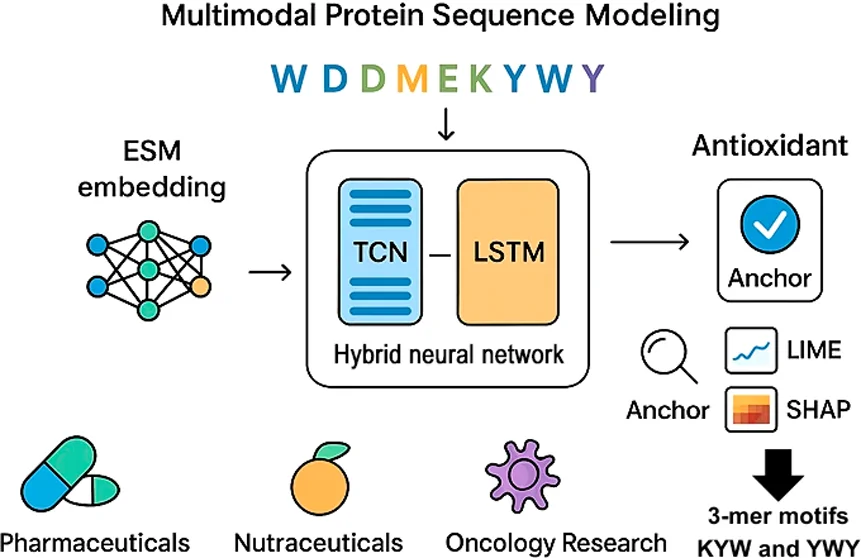

## Introduction

Antioxidant peptides represent a promising class of bioactive molecules with growing relevance in human health.^1–10^ These short amino acid sequences possess intrinsic free-radical-scavenging abilities, enabling them to neutralize reactive oxygen species (ROS) and reactive nitrogen species (RNS), key drivers of oxidative stress.^11,12^ In physiological systems, oxidative stress is closely linked to cellular aging,^13–15^ chronic inflammation,^16–18^ metabolic disorders,^19–21^ and oncogenic transformation,^6,9,22^ making antioxidant peptides attractive as therapeutic agents. Their activity stems from multiple mechanisms, including free-radical scavenging, metal-ion chelation, hydrogen donation, and modulation of endogenous antioxidant pathways, often mediated by specific sequence motifs enriched in residues such as histidine, proline, and aromatic amino acids. Therefore, the antioxidant activity of peptides is generally attributed to specific amino acid residues whose side-chain groups exhibit redox potential, such as the thiol group in cysteine (C), the thioether in methionine (M), the phenolic hydroxyl in tyrosine (Y), the indole ring in tryptophan (W), and the imidazole group in histidine (H).^11,23,24^ Peptides containing histidine or other coordinating residues bind transition-metal ions such as iron and copper, which are catalysts of radical formation through Fenton reactions. This chelation inhibits ROS generation by preventing metal-catalyzed oxygen reduction.^25^ Specific synthetic peptides have been shown to act via dual modes, both chelating metal ions and scavenging radicals effectively, demonstrating how sequence motifs drive antioxidant potential.^25–27^ Unlike synthetic antioxidants, these peptides are derived from natural protein sources or rational design, offering biocompatibility, low toxicity, and tunable bioactivity, which has driven their increasing exploration in nutraceuticals, dermatological formulations, and cancer therapeutics.

In food science, antioxidant peptides are known for their ability to delay lipid peroxidation, which helps extend the shelf life of functional foods and simultaneously offers health benefits when used as dietary supplements. These peptides, often rich in acidic amino acid residues like glutamic acid (E) and aspartic acid (D), work by scavenging free radicals such as superoxide anions and preventing the formation of lipid peroxides.^28–31^ They also hold promise as peptides capable of mitigating oxidative stress-related skin aging, enhancing wound healing, and protecting against UV-induced damage.^6,13,32–38^ Within oncology, the potential of antioxidant peptides extends beyond simple ROS neutralization, as they may play dual roles in both cancer prevention and therapy by modulating redox signaling pathways that influence tumor progression.^6,7,9,22,39–46^ This broad spectrum of applications highlights their versatility but also underscores the urgent need for systematic methods to understand and optimize their structure–function relationships.

Despite their promise, the discovery and rational design of antioxidant peptides face substantial challenges. Experimental identification remains resource-intensive, and traditional bioinformatics approaches often rely on sequence similarity rather than on a mechanistic understanding of functional motifs. Current models rarely provide interpretability, which is crucial for identifying sequence features (such as *k*-mer motifs and residue patterns) that underpin antioxidant activity. The absence of transparent computational models limits the ability to guide rational design strategies for next-generation bioactive molecules with improved stability, potency, and selectivity for specific applications in food science, dermatological therapeutics, and cancer research. Addressing this gap requires explainable artificial intelligence (XAI) frameworks capable of bridging predictive accuracy with interpretability, thereby uncovering actionable biological insights.

Traditional machine learning (ML) approaches for predicting antioxidant peptides often rely heavily on manually engineered global features (such as amino acid composition, physicochemical descriptors, sequence fingerprints, pairwise residue composition, and derived indices) rather than representations that retain residue order, semantic context, or evolutionary or structural information. These methods, while simpler and more interpretable in some aspects, tend to suffer from overfitting, poor generalization, and underperformance, particularly when the data are limited, heterogeneous in sequence length, or when key functional motifs are subtle. For example, the modeling of antioxidant peptides presented modest and variable performance, indicating considerable residual error and suggesting that many important sequence-semantic effects were not captured by simply selecting among global descriptors.^47^ Similarly, some models built using features, though performing well on some metrics, were limited in retaining sequence-specific information during training (i.e., relative order, positional motifs) and thus may miss functional *k-mer* or motif effects. Furthermore, their good performance was tied to strong feature engineering and feature fusion but still lacked explicit modeling of sequence semantics.^48^ In addition, the structure-antioxidant activity relationship of peptides in free radical systems found that the predictive power is highly dependent on terminal residue identity; however, such models tend to generalize poorly to peptides whose functional motifs are internal or distributed.^49^ These studies^50–54^ illustrate that while traditional ML with feature engineering can yield reasonably good results, it often fails to capture the semantic richness of peptide sequences: the order of residues, interactions between nonadjacent *k*-mers, evolutionary conservation, and subtle structural context. Consequently, these methods tend to overfit on data sets of limited sample size or biased composition and underperform when predicting activity for novel peptides outside the training distribution.^55^ Some analyses focusing on terminal-position descriptors similarly demonstrate dependency on specific positions but generalize poorly when functional determinants are internal or distributed, again pointing to the inability of simple global descriptors to represent sequence context.^56^ Recent surveys and method comparisons also highlight that oversimplified encodings and small training sets cause instability of predictive performance, motivating the move toward learned, context-rich representations and model architectures that explicitly capture *k-mer* motifs and long-range dependencies.^57^

This study presents a comprehensive XAI-driven framework for the classification and interpretation of the antioxidant peptides. The data set used in this study has two classes of antioxidant properties: free radical scavenger and chelation; however, the free radical scavenger (FRS) class was the only class used in this study. Therefore, the reported motif interpretations are limited to the free-radical scavenging class. It is important to mention that antioxidant activity is mechanistically diverse and that future studies incorporating multiple antioxidant mechanisms, including metal chelation, could provide a more comprehensive understanding of the sequence determinants underlying the different modes of antioxidant action. As BiLSTM-based architectures for antioxidant peptides demonstrate strong predictive performance on curated antioxidant peptide data sets,^52^ we introduce a hybrid deep learning model that integrates ESM embeddings with temporal convolutional networks (TCNs) and long short-term memory (LSTM) layers^58^ to capture both local and global biochemical features of peptide sequences. TCNs offer a computationally efficient alternative to recurrent models for detecting motif patterns distributed across the sequences. Such results motivate the use of TCN modules as local motif detectors in peptide classifiers that must be sensitive to short *k-mer* signals.^59^ Recurrent architectures, particularly LSTM-based networks, may be efficiently used for peptide classification tasks where modeling sequential context is crucial. Combining recurrent and convolutional pathways therefore allows local motif sensitivity and long-range context aggregation.^60^ ESM embeddings provide evolutionary and structural context for each residue, while TCN and LSTM layers complement each other by extracting motif-level patterns and sequential dependencies, respectively. Beyond predictive modeling, this framework focuses on interpretability using high-precision model-agnostic anchor explanations (Anchor),^61^ local interpretable model-agnostic explanations (LIME),^62^ and SHapley Additive exPlanations (SHAP),^63^ offering complementary perspectives on how *3- and 6-mer* motifs contribute to antioxidant activity. Anchor identifies minimal, decisive motifs essential for classification; LIME provides interpretable local approximations, highlighting synergistic contributions; and SHAP quantifies the global feature importance in a mathematically grounded manner.

## Methodology

The reliability of any machine learning model is fundamentally dependent on the quality, representativeness, and balance of its underlying data set. In this study, nonantioxidant and antioxidant peptide sequences were systematically curated from the literature^53^ and preprocessed to ensure both biological relevance and computational robustness. Each peptide is labeled for two classes, free-radical scavenger (FRS) and chelator, but only the FRS class was used in this study. The FRS class was labeled 1 (positive, antioxidant) if its source had measured/indicated an activity and otherwise 0 (negative, non-FRS). Therefore, the original data set consisted of a curated library of 688 experimentally validated antioxidant peptides, annotated as antioxidant = 1, and 229 nonantioxidant experimentally validated peptides, annotated as nonantioxidant = 0. Before these preprocessing steps (undersampling, sequence-level mix-up augmentation, and sampling), we also randomly separated 20 peptide samples (10 antioxidant and 10 nonantioxidant peptides) from the original data set for model testing (testing data set 1). We also used a testing data set with 31 antioxidant peptides from the APD6 database.^64^ It is important to mention that these 31 antioxidant peptides were not included in the original data set.

To address class imbalance, which is a frequent challenge in antioxidant prediction where antioxidant peptides typically outnumber nonantioxidant peptides, the data set was balanced through undersampling. The majority class (antioxidant peptides) was reduced to match the size of the minority class (nonantioxidant peptides), thereby preventing data duplicity and bias toward positive predictions. Both classes were then randomly shuffled and resampled to yield a fixed number of sequences per class (*n = 219*), ensuring equal representation and improving statistical fairness.

Given the intrinsic challenges of biological sequence data (such as limited sample sizes, redundancy, and class imbalance), a multistage augmentation and sampling strategy was implemented. First, a sequence-level mix-up augmentation was performed within each class using a combination of all 219 antioxidant peptides (229 × 229 = 47,961) and all 219 nonantioxidant peptides (219 × 219 = 47,961), totaling 95,922 peptides. Unlike conventional numeric mix-up, this approach concatenated peptide pairs from the same class, generating synthetic hybrids while retaining biologically meaningful antioxidant or nonantioxidant properties. Because the labels remained consistent with the source sequences, the augmented data preserved class fidelity while enriching sequence diversity, thereby improving the model’s ability to generalize across underrepresented sequence variations. The resulting data set was then rigorously cleaned to remove possible duplicate entries, reducing redundancy and preventing model overfitting to repeated sequences. We also removed peptide sequences that were either very short (<7 amino acids) with low semantic complexity or very long (>40 amino acids) to preserve structural homology, thereby avoiding the inclusion of sequences of little relevance to the study. Then, we sorted 20,000 peptides for each class, totaling 40,000 peptides with reduced redundancy to avoid overfitting. From our experience, this data set size is suitable for training and validation using advanced deep learning strategies. However, it is important to mention that synthetic sequences may not necessarily preserve the true antioxidant properties of the original peptides.

The resulting data set thus represented a carefully curated, synthetically diversified, and statistically balanced collection of peptide sequences. This design allowed the downstream model to learn discriminative patterns with a reduced risk of overfitting, class bias, and data sparsity. Additional safeguards were implemented at the beginning of the data preprocessing and included a stratified train-validation split that preserved class ratios and enforced >90% identity and redundancy filters to prevent homologous sequences from being distributed across partitions, thereby avoiding overly optimistic performance estimates. High-similarity motif pairs were identified by using Jaccard similarity coefficients exceeding 0.80, providing a stringent criterion for shared local sequence patterns. The results indicate significant *6-mer* motif leakage in both augmented data sets (with and without splitting), suggesting that augmentation procedures tend to preserve or replicate local sequence fragments. This is particularly problematic in peptide classification tasks where short motifs often encode biochemical functionality. Cosine similarity thresholds greater than 0.95 were used to identify semantically equivalent or highly similar sequences across the splits. This analysis captures deeper relationships beyond direct sequence identity, including structural or functional similarity encoded in the latent space. The findings show that semantic leakage is minimal in the original data set but becomes pronounced in the augmented data sets, reinforcing the notion that augmentation can generate embeddings that are nearly indistinguishable across training and validation sets. This is especially critical for transformer-based models, where the embedding similarity strongly correlates with predictive behavior.

To further quantify cross-split dependencies, a neighborhood-based approach was employed by using the Nearest Neighbors implementation from scikit-learn. By identifying *k*-nearest neighbors (*k* = 5) across training and validation sets, the analysis reveals the extent to which validation samples are embedded within the local manifold of the training data. The presence of cross-split nearest neighbors indicates that validation sequences may not represent truly independent samples, thereby compromising the validity of the performance metrics. This effect is again most prominent in the augmented data sets, where dense sampling of sequence space leads to overlapping neighborhoods.

From a methodological standpoint, the integration of CD-HIT clustering, k-mer Jaccard similarity, cosine similarity in the embedding space, and k-nearest neighbor analysis provides a robust framework for leakage auditing. For researchers developing explainable models (e.g., SHAP analyses on embeddings or physicochemical descriptors), controlling for such leakage is essential to ensure that identified features reflect genuine biological signals rather than artifacts of data set construction.

For input encoding, peptide sequences were transformed into one-hot representations with a maximum input length of 100 residues, applying zero-padding symmetrically to shorter peptides while avoiding truncation of longer ones. This ensured consistency in tensor dimensions for the TCN pathway. To further enhance robustness against positional artifacts, a reverse-sequence augmentation strategy was introduced to increase the diversity of the training data while preserving class labels. In this approach, each peptide sequence was complemented with its reversed counterpart (e.g., RRRKKK → KKKRRR). Although the reversed sequence does not represent a new biological peptide in terms of amino acid composition, it constitutes a distinct input representation for the neural network, allowing the model to observe alternative sequence arrangements during training. This augmentation strategy is commonly employed in deep learning frameworks, including methodologies available within the PyTorch ecosystem, to improve model robustness, reduce overfitting, and encourage the learning of more generalizable sequence patterns. By exposing the network to both forward and reversed representations, the model becomes less sensitive to positional biases and can better capture compositional and contextual features relevant to peptide classification. As the final data set was strictly balanced, a simple 80/20 random stratified data split was used with two additional independent test data sets, and no additional k-fold or nested cross-validation was deemed necessary for model optimization. The model performance was obtained using the following metrics: accuracy, precision, recall, F1-score, Matthews correlation coefficient (MCC), Cohen’s Kappa, and confusion matrix. Collectively, this pipeline integrated biologically informed redundancy and identity sequence filtering, augmentation, and balancing procedures, as presented in Figure 1, thereby providing a rigorous foundation for embedding extraction, model optimization, and explainability analyses.

**Figure 1:**
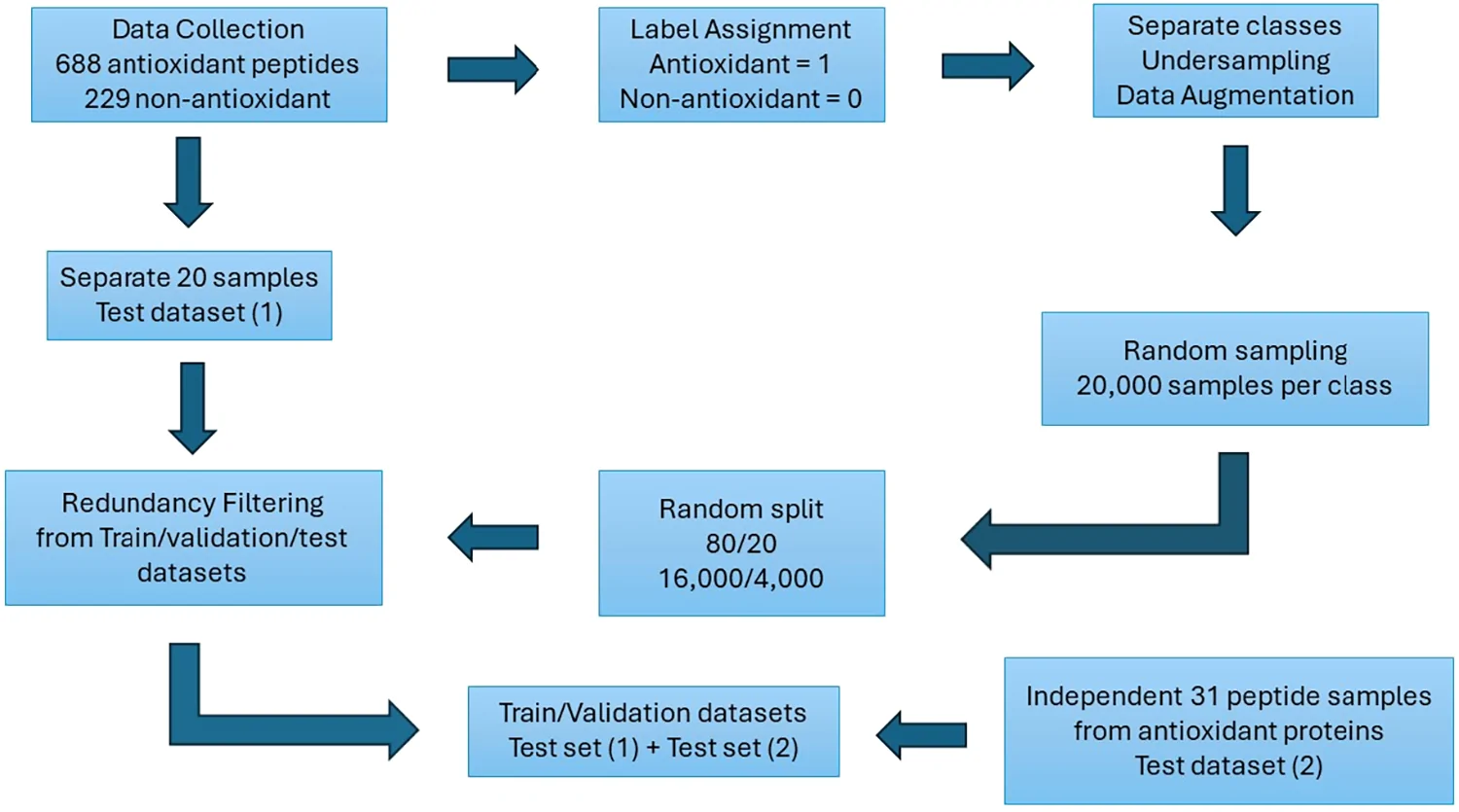
Step-by-step data set construction pipeline, including the steps for sampling, random split, identity and redundancy sequence filtering, and train/validation/test separation for the prevention of overlap between subsets.

Then, this work introduces a multimodal deep learning framework designed to classify antioxidant peptides by integrating advanced sequence modeling methods with an interpretable artificial intelligence. The architecture leverages three complementary components: ESM embeddings,^65–68^ TCNs, and LSTM layers using the PyTorch library. Together, these components capture both local biochemical patterns and global contextual features within the peptide sequences. ESM, a transformer-based protein language model trained on millions of protein sequences, provides high-dimensional embeddings that encode the evolutionary and structural contexts of amino acids. These embeddings represent each residue in a biologically meaningful way, capturing conservation, biochemical similarity, and higher-order sequence dependencies that are often invisible to alignment-based techniques. Averaging these residue embeddings produces a condensed peptide-level representation that serves as a biologically informed prior for the classification.

Complementing the ESM embeddings, the TCN module focuses on extracting short-range sequence motifs that contribute to the antioxidant activity. Unlike recurrent networks, TCNs use dilated one-dimensional convolutions, which allow them to achieve exponentially large receptive fields while preserving the sequence order. This makes them particularly effective for modeling peptides, where motifs separated by variable numbers of residues can exert a significant influence on biological function. TCNs act as localized motif detectors, systematically scanning the sequence for patterns of amino acids that encode structural features relevant to radical scavenging or redox activities. By leveraging dilation, TCNs efficiently capture both adjacent residue interactions and medium-range dependencies without the vanishing gradient issues that are common in standard recurrent architectures.

In parallel, the LSTM network models long-range sequential dependencies at the peptide level. LSTMs are well-suited for learning context over sequential inputs because their gating mechanisms (input, forget, and output gates) regulate information flow across long distances in the sequence. When fed with the biologically enriched ESM embeddings, the LSTM learns higher-order sequence semantics, effectively integrating the global structural and biochemical context. This sequential reasoning is critical because antioxidant activity often depends not only on local motifs but also on how these motifs are distributed within the peptide and how they interact in a three-dimensional space. By combining the locality of TCNs with the sequential reasoning of LSTMs, the model captures information across multiple biological scales.

A fusion module integrates the outputs of the TCN and LSTM streams, adaptively weighting their contributions based on the learned gating mechanisms. This multimodal integration enables the classifier to dynamically emphasize structural information from ESM embeddings or motif-level information from TCN features, depending on the characteristics of each peptide. The fusion strategy is particularly powerful in peptide research, where antioxidant activity is rarely defined by a single motif or global pattern but rather emerges from complex, hierarchical interactions between amino acids and their physicochemical properties.

The optimization of the proposed TCN+LSTM framework was performed through a combination of architectural design choices and training regularization strategies aimed at maximizing generalization while minimizing overfitting. Initially, the one-hot encoded peptide sequences were processed through a temporal convolutional network (TCN) composed of residual dilated convolutional blocks with exponentially increasing dilation factors, batch normalization, GELU activation functions, dropout regularization, and Kaiming weight initialization. The TCN-derived local sequence features were then integrated with the ESM embeddings through an adaptive gated fusion mechanism that dynamically learns the relative contribution of each representation. The fused features were subsequently processed by a three-layer LayerNorm-LSTM architecture with a hidden size of 128 and dropout regularization, enabling the capture of long-range sequence dependencies and contextual relationships. Model training employed the AdamW optimizer with an initial learning rate of 1 × 10^–4^ and weight decay of 1 × 10^–4^, combined with label-smoothing cross-entropy loss (smoothing factor = 0.1), to improve calibration and reduce overconfident predictions. Additional optimization techniques included gradient accumulation (two mini-batches per update), gradient clipping (maximum norm = 1.0) to stabilize training, learning-rate warmup during the first ten epochs, and adaptive learning-rate reduction using ReduceLROnPlateau when validation loss stagnated. The optimization process was monitored through validation loss, and an early stopping criterion with a patience of 10 epochs was employed, saving the best-performing model and restoring its parameters whenever validation performance ceased to improve.

Beyond predictive performance, this framework prioritizes interpretability, making use of state-of-the-art XAI techniques: Anchor,^61^ LIME,^62^ and SHAP.^63^ These methods offer complementary insights into the modeĺ’s decision-making process. Anchor^61^ provides high-precision, rule-based explanations by identifying minimal sets of *k-mers* (short, overlapping substrings of the peptide) that are sufficient to guarantee the model’s classification with high confidence. This is especially valuable for experimental peptide design because it isolates core sequence motifs strongly associated with antioxidant activity, enabling rational modifications of peptide candidates.

LIME^62^ offers a locally linear approximation of the model’s behavior by perturbing the input sequence and measuring the impact of these perturbations on prediction probabilities. In practice, LIME^62^ generates an interpretable weighting of each *k-mer*, revealing how different parts of the sequence collectively drive the prediction. While Anchor focuses on minimal decisive motifs, LIME^62^ exposes the synergistic and antagonistic contributions of amino acid regions, providing a more nuanced view of peptide functionality and potential compensatory effects between residues.

SHAP^63^ complements these local approaches with a theoretically grounded, additive attribution framework. Rooted in cooperative game theory, SHAP^63^ quantifies the contribution of each feature (in this case, residues or *k-mers*) to the final prediction relative to a baseline expectation. This allows the aggregation of SHAP^63^ values across sequences, uncovering global trends and motifs that consistently drive antioxidant activity classification. SHAP values quantify the contribution of each input feature to the model’s prediction relative to a baseline prediction. In this study, the absolute magnitude of a SHAP value reflects the strength of a k-mer motif’s influence on the predicted antioxidant activity, whereas the sign indicates whether the motif increases or decreases the predicted probability of belonging to the antioxidant class. Consequently, motifs with larger absolute SHAP values have a greater impact on the model’s decision, although these values represent the model’s learned associations rather than direct experimental evidence of biological function. Unlike LIME^62^ or Anchor,^61^ SHAP^63^ provides a mathematically rigorous decomposition of the model’s decision into additive contributions, enabling systematic identification of residues that have cooperative or antagonistic effects across diverse peptide contexts.

The integration of these XAI tools creates a multiresolution interpretability framework: Anchor^61^ highlights deterministic sequence elements, LIME^62^ provides human-readable local approximations of model reasoning, and SHAP^63^ delivers a comprehensive, quantitative view of feature influence across the data set. Together, they bridge the gap between high-performance prediction and biological interpretability, facilitating hypothesis generation and guiding experimental validation. The synergy among biologically informed embeddings, hybrid neural architectures, and layered explainability enables this framework not only to predict antioxidant activity with high confidence but also to illuminate the biochemical rationale underlying these predictions. This combination of predictive power and interpretability makes it a powerful tool for peptide discovery, drug development, and structural bioinformatics.

## Results

Figure 2 illustrates the training (blue curve) and validation (orange curve) loss trajectories over 35 epochs during the optimization of a deep learning framework designed for antioxidant peptide classification. This model integrates TCN and LSTM layers with embeddings derived from the ESM-2 protein language model (esm2_t6_8M_UR50D). The ESM embeddings provide rich, context-aware representations that capture evolutionary constraints and semantic relationships among amino acids, while the TCN and LSTM layers extract hierarchical local motifs and sequential dependencies that are critical for peptide activity prediction.

**Figure 2:**
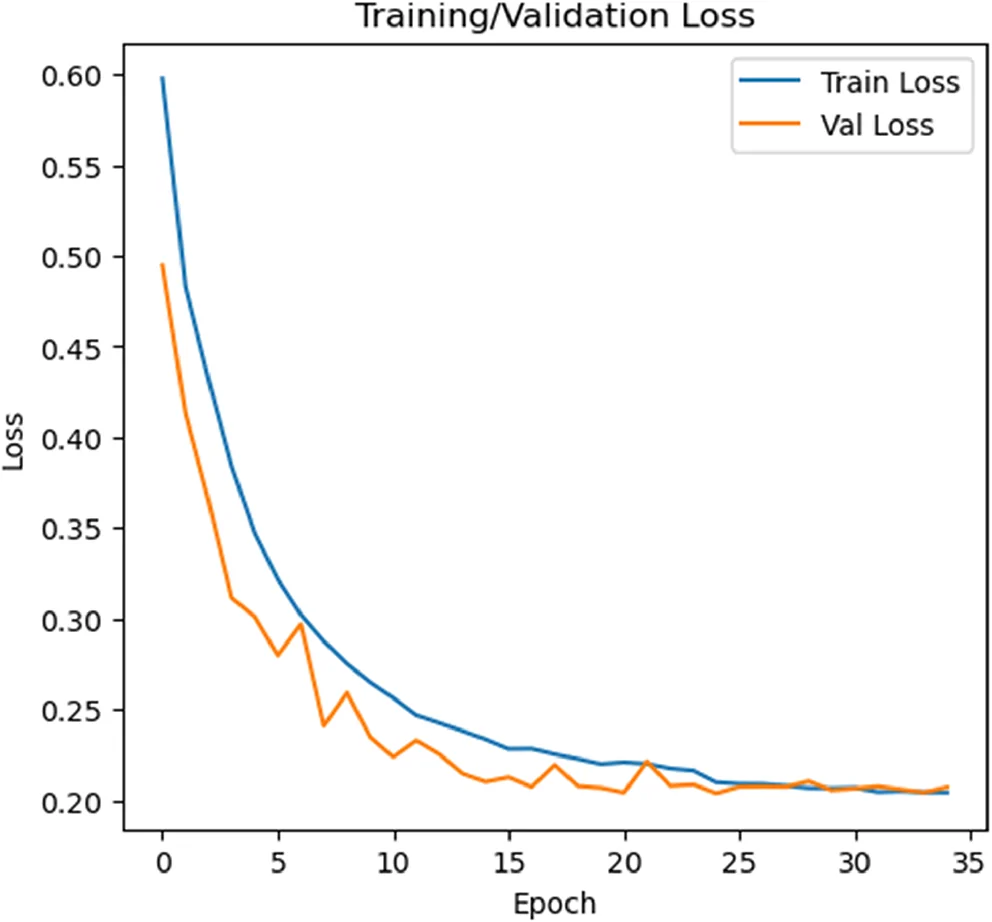
Training (blue line) and validation (orange line) losses for balanced augmented data set with 35 epochs during optimization of the deep learning model (TCN + LSTM) to respectively classify antioxidant peptides using an Evolutionary Scale Modeling (ESM) language model (esm2_t6_8M_UR50D) to capture both global semantic and local structural features.

The loss curves show a steep decline during the initial training epochs (0–10), reflecting rapid convergence as the model learns discriminative sequence features. After epoch 10, both curves progressively plateau, indicating stabilization of learning and diminishing marginal improvements in performance. Notably, the validation loss closely tracks the training loss, with minimal divergence, which demonstrates strong generalization to unseen data and indicates that the data set augmentation and balancing strategies effectively mitigated overfitting, a common challenge in peptide-based predictive modeling where data sets are often small and imbalanced. By approximately epoch 20, the validation loss achieves near-convergence at ∼0.20, suggesting robust predictive accuracy and a well-regularized model capable of identifying antioxidant-specific motifs.

This history underscores the effectiveness of integrating pretrained protein language models with hybrid TCN-LSTM architectures. The ESM embeddings act as a semantic scaffold, allowing the model to generalize beyond simple amino acid composition toward deeper structural and functional correlations of the antioxidant activity. The smooth convergence patterns further validate that this pipeline, aided by data augmentation, achieves a balanced compromise between model expressiveness and generalization, which is essential for designing interpretable AI frameworks that can guide peptide optimization in biomedical applications.

Table 1 summarizes the evaluation metrics of the optimized deep learning framework, which integrates TCN and LSTM layers with embeddings from the ESM-2 protein language model (esm2_t6_8M_UR50D). This model was designed to classify antioxidant peptides by capturing both global semantic relationships among amino acids (derived from the pretrained ESM embeddings) and local sequential patterns extracted through the TCN and LSTM layers. The results demonstrate exceptional classification performance across both the training and validation data sets, reflecting both high predictive capacity and generalization.

**Table 1:** Performance Statistical Metrics (Accuracy, Precision, Recall, F1 Score, Matthews Correlation Coefficient (MCC), and Cohen’s Kappa) of Training and Validation Data sets after Optimization of the Deep Learning Model (TCN + LSTM) to Classify Antioxidant Peptides Using an Evolutionary Scale Modeling (ESM) Language Model (esm2_t6_8M_UR50D) to Capture Both Global Semantic and Local Structural Features

data set	accuracy	precision	recall	F1 score	MCC	Cohen’s Kappa
training	0.999	1.000	0.999	0.999	0.999	0.999
validation	0.997	1.000	0.995	0.997	0.995	0.995

On the training set, the model achieved an accuracy of 99.94%, with a precision of 100%, a recall of 99.89%, and an F1-score of 99.94%. These values demonstrate that the model almost perfectly separates antioxidant from nonantioxidant peptides, with negligible false positives or false negatives. The Matthews correlation coefficient (MCC) and Cohen’s kappa, both at 99.89%, further reinforce this excellent training, as these metrics are particularly robust to class imbalances and approach their theoretical maximum, indicating near-perfect classification agreement.

Importantly, the model maintains exceptional generalization on the validation set, achieving an accuracy of 99.75%, a precision of 100%, a recall of 99.50%, and an F1-score of 99.75%. Again, MCC and Cohen’s kappa remain extremely high (0.9950), demonstrating strong reliability and consistency of predictions across independent data. The slight reduction from training to validation metrics is expected and reflects a well-regularized model rather than overfitting, a particularly significant achievement, given the complexity of peptide sequence data and the inherent variability of biological data sets.

It is important to mention that most data sets contain peptides with mixed antioxidation mechanisms (direct redox activity, metal chelation, structural effects, and electrostatic interactions). Our model aims to classify antioxidant peptides by free radical scavenging, as stated in the paper’s objectives. We curated two data sets (Peptipedia^69^ and AnOxPePred^53^) to remove antioxidant peptides trained by free radical scavenging in our data set. Using our trained model, we also tested only the antioxidant peptides by free radical scavenging, resulting in 59 true positives from 63 samples (an accuracy of 90.48%) in the Peptipedia^69^ data set, and 27 true positives from 28 samples (an accuracy of 96.43%) in the AnOxPePred^53^ data set. These tests prove that our model works well with antioxidant peptides. Also, we tested both antioxidant and nonantioxidant peptides from the AOPP,^51^ AnOXPP,^52^ and AnOxPePred^53^ data sets (containing mixed antioxidation mechanisms) after filtering the peptides trained in our data set. As expected, we found poor accuracies: 41.18% for AOPP,^51^ 41.64% for AnOxPP,^52^ and 58.01% for AnOxPePred.^53^ This shows the importance of curating the data sets with only the free radical scavenging mechanism for our model to predict excellent results.

The metrics in Table 1 illustrate the synergistic effect of combining ESM embeddings with a hybrid TCN-LSTM architecture: pretrained protein language models provide contextualized amino acid embeddings that encode evolutionary and structural information, while the sequential modeling layers enable the detection of *k-mer* motifs statistically associated with antioxidant peptide classification and long-range dependencies. This combination yields a robust, high-precision classifier capable of distinguishing antioxidant peptides with exceptional confidence. Such performance metrics indicate not only the effectiveness of this deep learning approach but also its suitability for downstream interpretability analyses (e.g., Anchor, LIME, and SHAP), where high classification reliability is essential to extract interpretable insights into the sequence patterns learned by the model about antioxidant peptide motifs.

Figure 3 presents the confusion matrices for both the training (left) and validation (right) data sets after 35 epochs of optimizing a deep learning framework that integrates TCN and LSTM layers with the ESM-2 protein language model (esm2_t6_8M_UR50D). This architecture was designed to leverage the semantic richness of pretrained ESM embeddings (which encode evolutionary and structural information about amino acids) and combine them with sequential modeling layers capable of extracting local motifs and long-range dependencies that are crucial for distinguishing antioxidant peptides.

**Figure 3:**
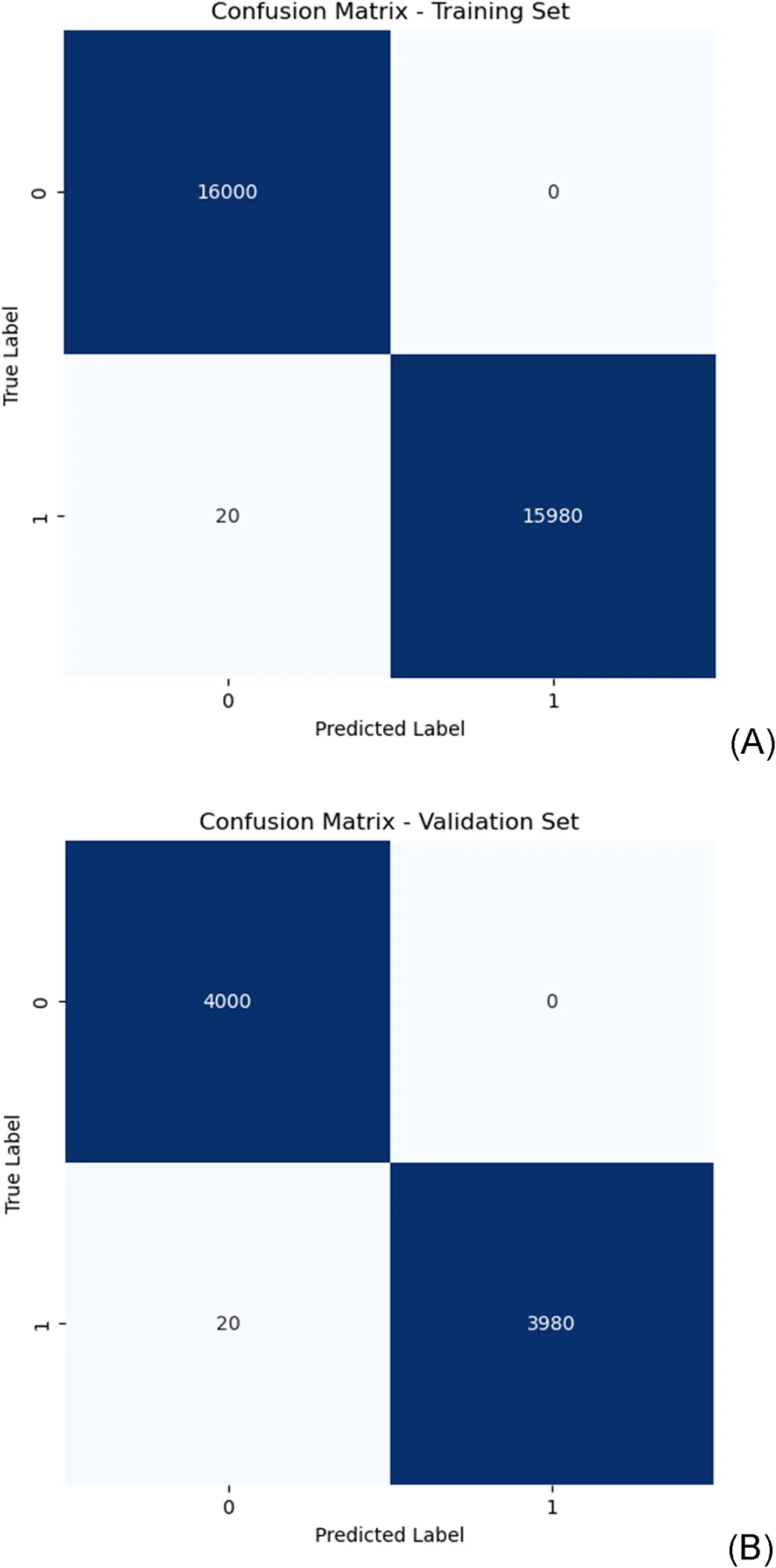
Confusion matrices of training (A) and validation (B) data sets after 35 epoch optimizations of the deep learning model (TCN + LSTM) to respectively classify antioxidant peptides using an Evolutionary Scale Modeling (ESM) language model (esm2_t6_8M_UR50D) to capture both global semantic and local structural features.

The training confusion matrix reveals an almost perfect classification: all 16,000 negative samples (class 0) were correctly classified, and 15,980 out of 16,000 positive samples (class 1) were correctly predicted, with only 20 false negatives and no false positives. This near-ideal performance reflects the model’s ability to fully capture the discriminative features of antioxidant peptides, demonstrating high sensitivity and specificity without overfitting. The validation confusion matrix shows similarly strong performance: all 4000 negative samples were correctly classified, while only 20 false negatives were observed among 4000 positive samples, yielding 3980 correct predictions. The consistency between the training and validation matrices strongly indicates robust generalization, even when evaluated on unseen data. This is a critical achievement given the complexity and heterogeneity of peptide sequence data sets, where overfitting is a persistent challenge.

These results highlight the model’s capability to accurately separate antioxidant peptides from nonantioxidant sequences with exceptional confidence. The absence of false positives in both data sets emphasizes the model’s conservative decision boundary, minimizing the likelihood of incorrectly classifying inactive peptides as antioxidant candidates, a key requirement in biomedical and therapeutic peptide discovery pipelines. Furthermore, this high level of predictive accuracy provides a strong foundation for XAI analyses (Anchor, LIME, SHAP), ensuring that the motifs and *k-mer* patterns identified as determinants of antioxidant activity are both biologically meaningful and reliably inferred.

To deepen the understanding of the decision-making processes underlying antioxidant peptide classification, we applied a multimethod interpretability framework combining Anchor, LIME, and SHAP analyses to sequences with diverse biochemical properties (Figures 3–7). These XAI approaches offer complementary insights: Anchor isolates minimal sets of motifs sufficient for deterministic predictions with full confidence, LIME provides a probabilistic local approximation of feature relevance, and SHAP quantifies global feature contributions across all possible combinations of motifs. Together, these methods provide a hierarchical and biologically grounded understanding of how the model leverages peptide sequence semantics encoded by the ESM embeddings to achieve accurate predictions.

For sequence 9 (**AVPYPQRRWH**) in Figure 4, Anchor pinpointed **VPY**, **RWH**, and **RRW** as terminally positioned motifs that were sufficient for 100% classification precision, indicating strong reliance on arginine- and histidine-rich residues known for radical scavenging and electrostatic interactions. LIME expanded this motif set, adding **QRR** and **PQR**, highlighting the importance of overlapping arginine clusters, while SHAP introduced **AVP** and **YPQ**, underscoring contributions from the peptide’s N-terminus. This convergence indicates that these motifs are consistently associated with the model’s predictions, although it does not establish a causal biochemical mechanism. It is primarily anchored in terminal motifs, with additional distributed features fine-tuning predictions. Similarly, for sequence 10 (**WDDMEKYWY**) in Figure 5, Anchor emphasized **WDD** as a decisive acidic motif, consistent with the role of negatively charged residues in metal chelation and redox activity. LIME revealed **KYW** and **YWY**, highlighting aromatic contributions, while SHAP reinforced **WDD** and added **MEK**, capturing the interplay between charged and aromatic residues. This triangulation underscores **WDD** as a motif strongly associated with the model’s prediction and potentially biologically meaningful, while emphasizing complementary motifs that influence structural stability and antioxidant potency. In sequence 11 (**YVEELYTY**) in Figure 6, Anchor and SHAP converged on **YVE** and **YTY**, motifs enriched in tyrosine, a residue central to free radical neutralization through phenolic chemistry. LIME introduced **ELY**, expanding interpretability to acidic residues modulating polarity. The alignment of findings across methods strengthens confidence that tyrosine-containing motifs are consistently recognized by the model, consistent with previously reported biochemical observations, although the present study does not directly demonstrate the underlying mechanism. For sequence 26 (**VHDYAWEEREQGSR**) in Figure 7, Anchor highlighted **AWE** and **VHD**, motifs involving histidine and tryptophan residues frequently implicated in antioxidant activity. LIME added **HDY** and **EQG**, while SHAP emphasized **QGS**, identifying additional internal motifs with moderate contributions. The diversity of motifs here demonstrates SHAP’s value in detecting subtle dependencies not captured by deterministic methods. Finally, sequence 29 (**IKKDEDTQAMP**) in Figure 8 revealed **KKD**, **DED**, and **KDE** as dominant motifs via Anchor, strongly pointing to charged residue clusters involved in metal chelation. LIME confirmed **KDE** and **DED**, while SHAP introduced **IKK** and **AMP**, capturing contributions from N-terminal lysine-rich regions and a hydrophobic C-terminal motif. Table 2 summarizes the Anchor, LIME, and SHAP explanations of the deep learning model (TCN + LSTM) with an Evolutionary Scale Modeling (ESM) language model to capture both global semantic and local structural features (3-mer motifs) important to interpret antioxidant peptides (sequences 9, 10, 11, 26, and 29).

**Figure 4:**
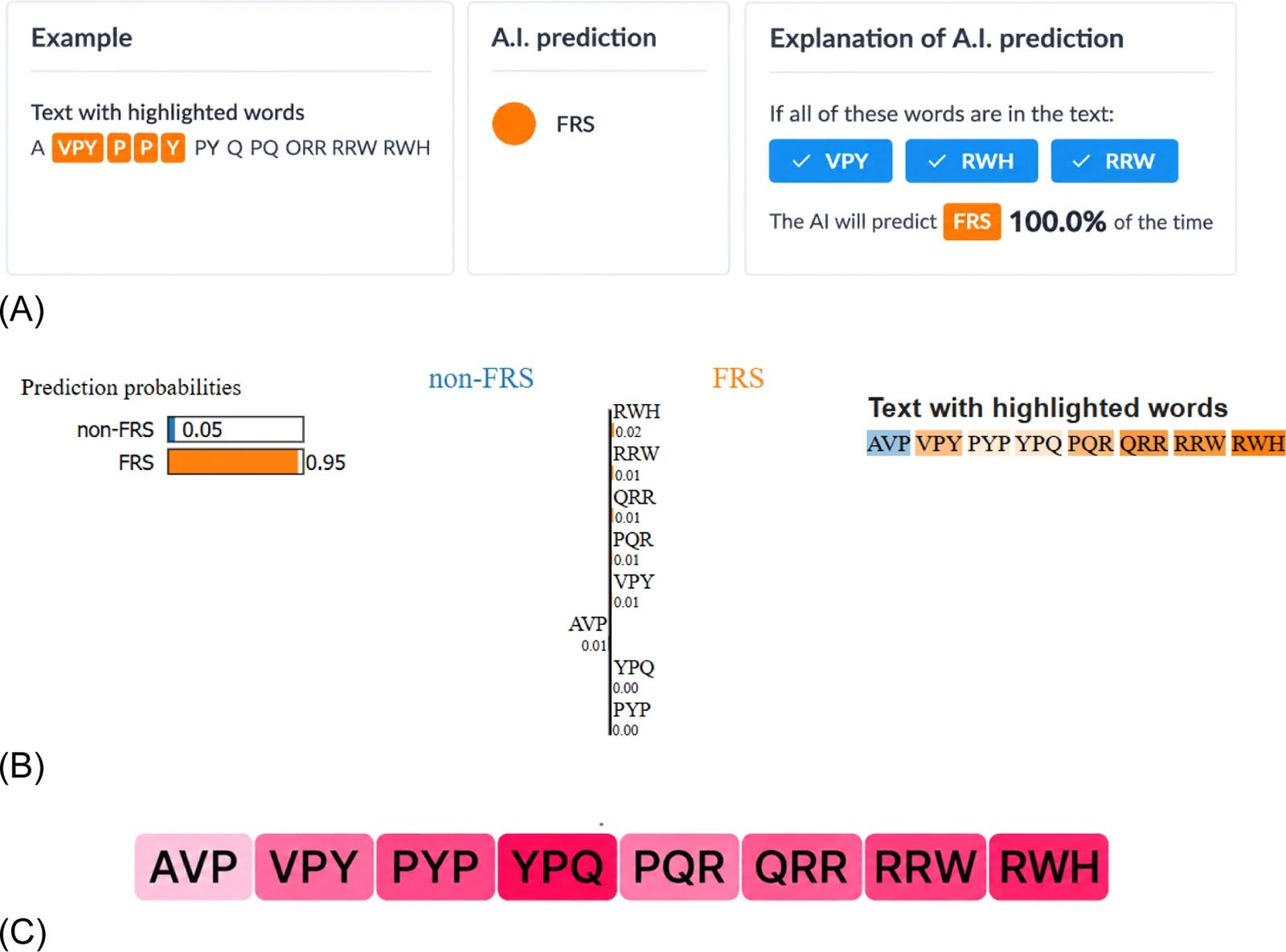
(A) Anchor, (B) LIME, and (C) SHAP explanations of the deep learning model (TCN + LSTM) with an Evolutionary Scale Modeling (ESM) language model (esm2_t6_8M_UR50D) to capture both global semantic and local structural features (*3-mer* motifs) important to interpret antioxidant peptides (sequence 10).

**Figure 5:**
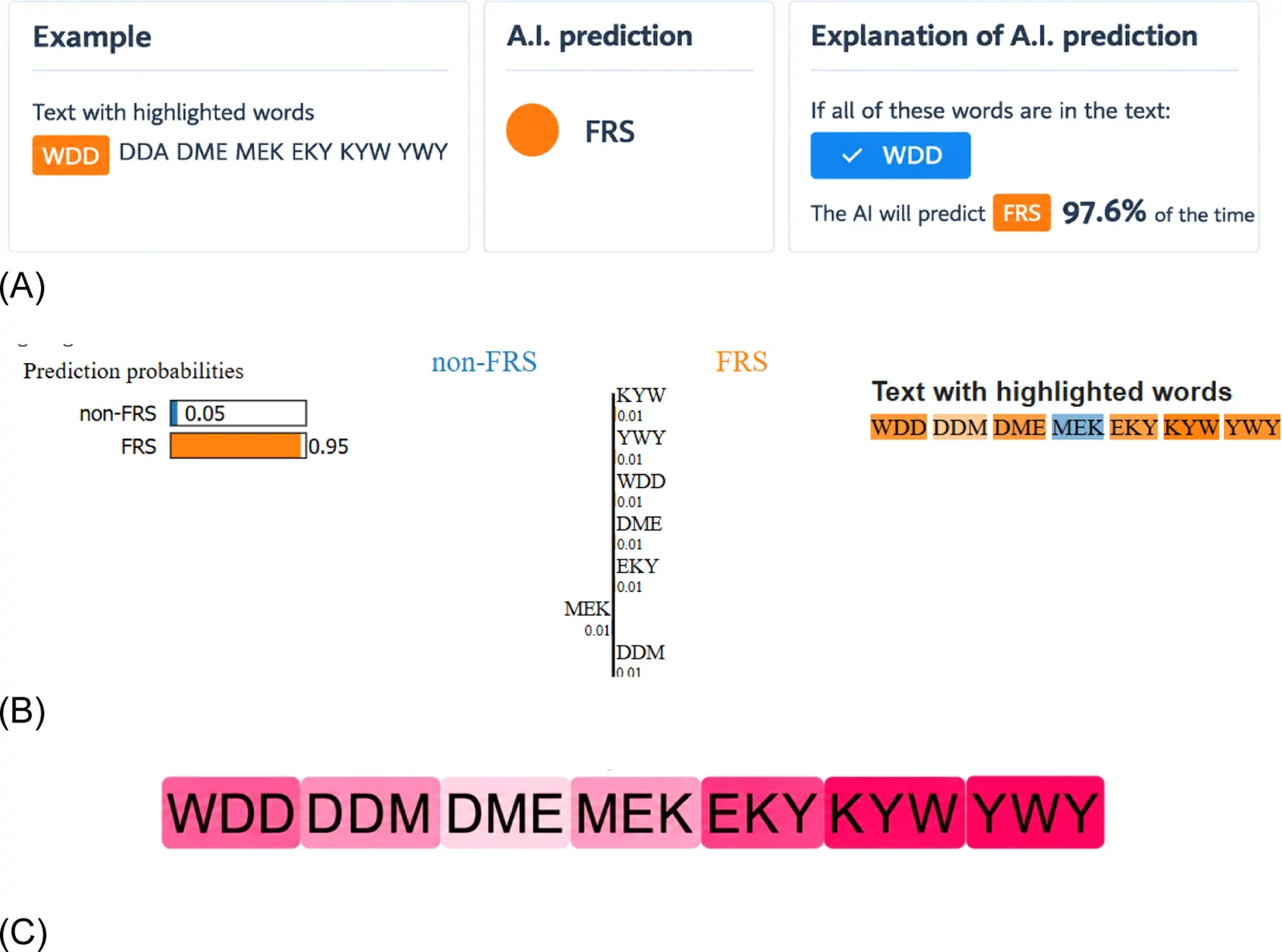
(A) Anchor, (B) LIME, and (C) SHAP explanations of the deep learning model (TCN + LSTM) with an Evolutionary Scale Modeling (ESM) language model (esm2_t6_8M_UR50D) to capture both global semantic and local structural features (*3-mer* motifs) important to interpret antioxidant peptides (sequence 10).

**Figure 6:**
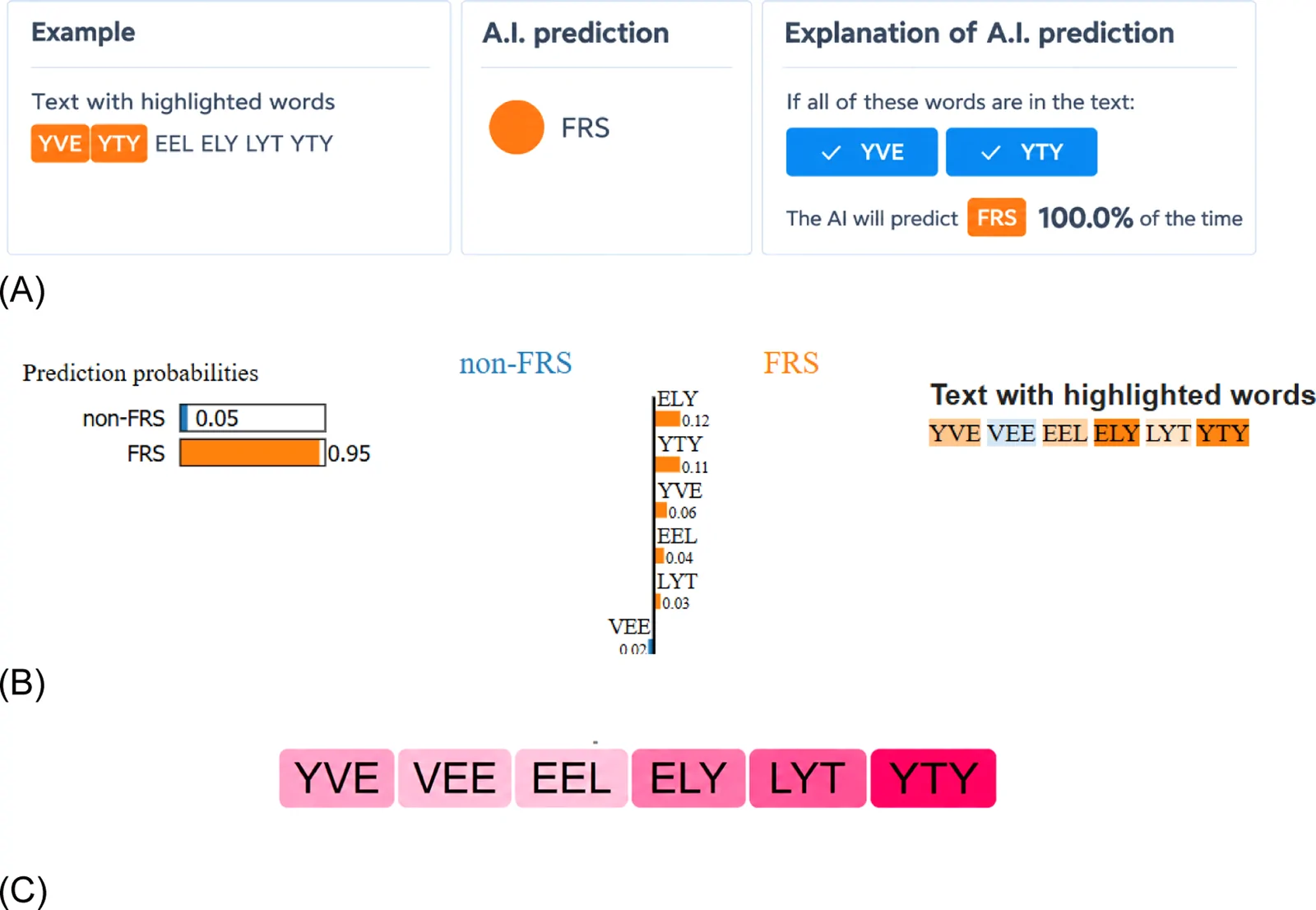
(A) Anchor, (B) LIME, and (C) SHAP explanations of the deep learning model (TCN + LSTM) with an Evolutionary Scale Modeling (ESM) language model (esm2_t6_8M_UR50D) to capture both global semantic and local structural features (*3-mer* motifs) important to interpret antioxidant peptides (sequence 11).

**Figure 7:**
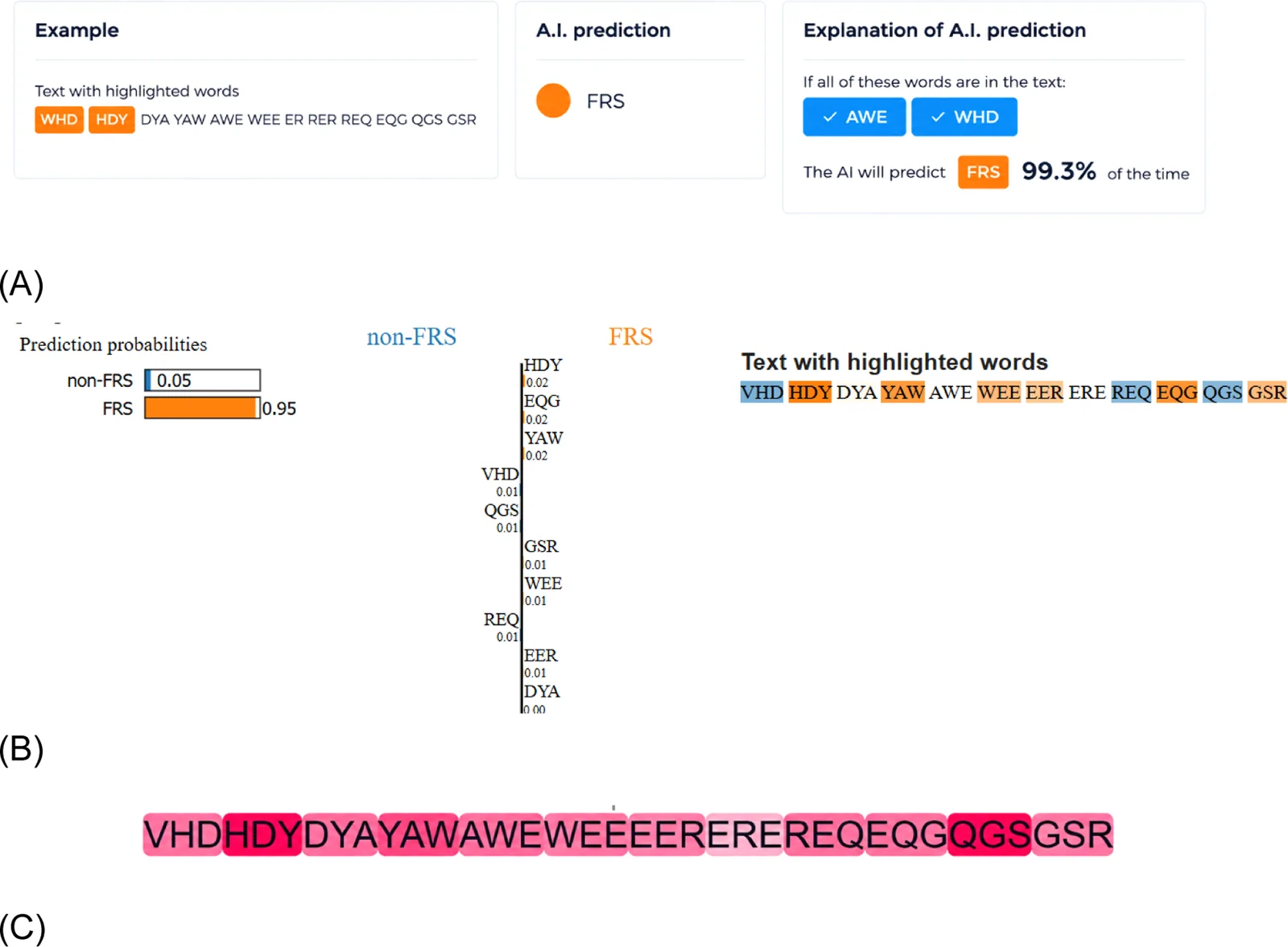
(A) Anchor, (B) LIME, and (C) SHAP explanations of the deep learning model (TCN + LSTM) with an Evolutionary Scale Modeling (ESM) language model (esm2_t6_8M_UR50D) to capture both global semantic and local structural features (*3-mer* motifs) important to interpret antioxidant peptides (sequence 26).

**Figure 8:**
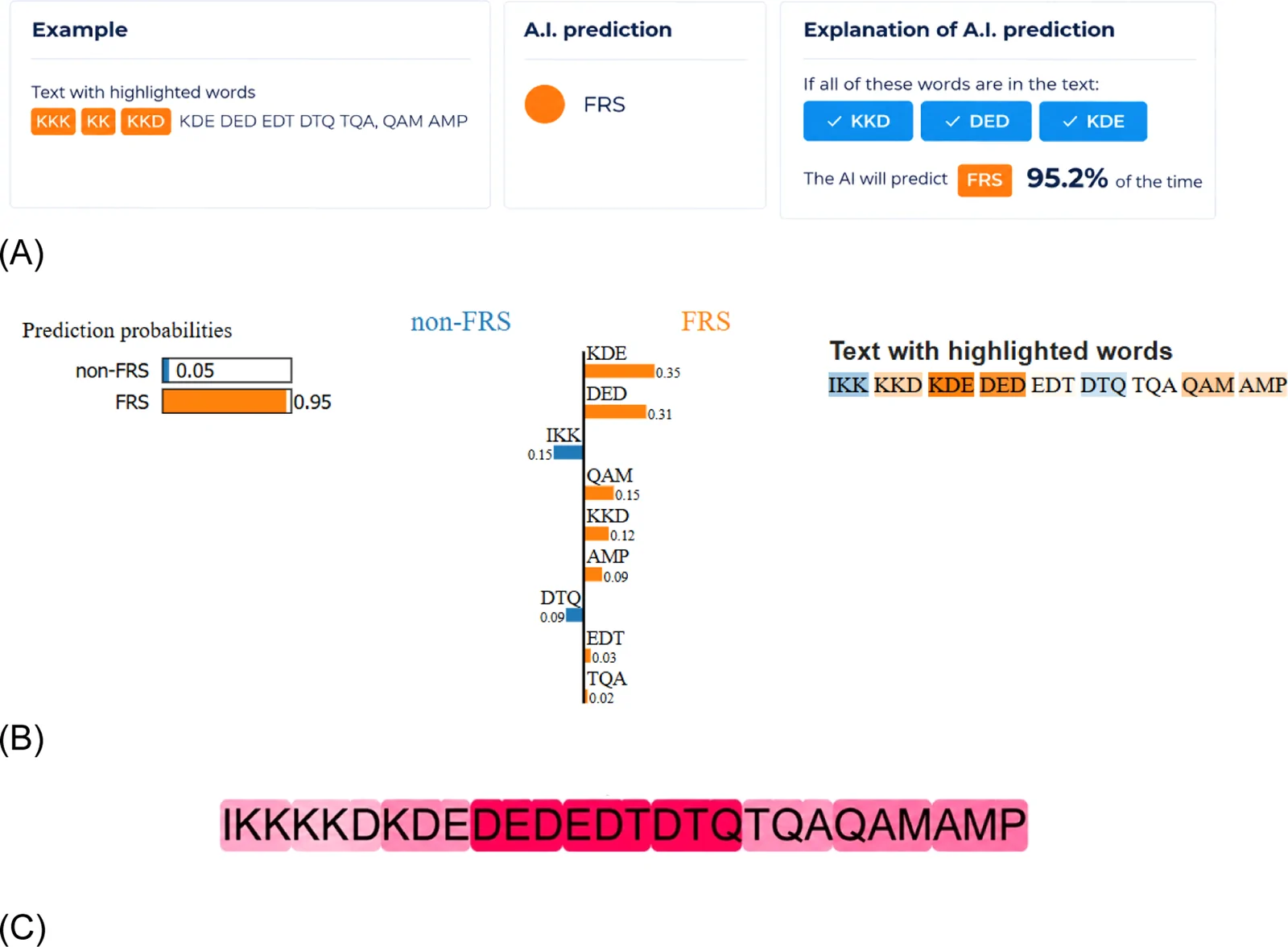
(A) Anchor, (B) LIME, and (C) SHAP explanations of the deep learning model (TCN + LSTM) with an Evolutionary Scale Modeling (ESM) language model (esm2_t6_8M_UR50D) to capture both global semantic and local structural features (*3-mer* motifs) important to interpret antioxidant peptides (sequence 29).

**Table 2:** Anchor, LIME, and SHAP Explanations of the Deep Learning Model (TCN + LSTM) with an Evolutionary Scale Modeling (ESM) Language Model (esm2_t6_8M_UR50D) to Capture Both Global Semantic and Local Structural Features (*3-mer* Motifs) Important to Interpret Antioxidant Peptides (Sequences 9, 10, 11, 26, and 29)

Peptide	Anchor	LIME	SHAP
AVPYPQRRWH	VPY	RWH	AVP
	RWH	RRW	YPQ
	RRW	QRR	RWH
		PQR	
		VPY	
WDDMEKYWY	WDD	KYW	WDD
		YWY	MEK
		WDD	YWY
		DME	
		EKY	
YVEELYTY	YVE	ELY	YVE
	YTY	YTY	YTY
VHDYAWEEREQGSR	AWE	HDY	HDY
	VHD	EQG	AWE
			QGS
IKKDEDTQAMP	KKD	KDE	IKK
	DED	DED	DED
	KDE		AMP

## Discussion

These analyses demonstrate that Anchor, LIME, and SHAP offer a complementary, multiperspective interpretability framework for antioxidant peptide classification. Anchor provides sparse, high-precision, rule-based anchors that guarantee predictions when motifs are present. This is ideal for extracting biochemically actionable motifs and offers a deterministic understanding of the model’s decision pathway, identifying minimal, high-confidence motifs that serve as deterministic biochemical “anchors” for classification. LIME introduces probabilistic feature rankings, capturing redundant and cooperative motifs that reflect how local perturbations affect predictions. It often highlights more motifs than Anchor, showcasing how multiple residue clusters contribute synergistically. SHAP delivers global, theoretically grounded feature attribution, quantifying each motif’s contribution across all possible feature combinations. It is especially effective in uncovering nonobvious, context-dependent influences from internal sequence positions or motifs outside the Anchor coverage. It offers globally consistent feature attribution, quantifying each motif’s contribution across all permutations. It highlights both terminal and internal subsequences, confirming the robustness of anchors and surfacing hidden sequence dependencies.

By integrating these perspectives, we constructed a hierarchy of motif relevance: motifs repeatedly identified across all three methods (e.g., **RWH**, **WDD**, **YTY**, and **DED**) emerge as the most consistently identified statistically associated motifs of antioxidant activity, while motifs unique to LIME or SHAP highlight exploratory avenues for hypothesis generation. This layered interpretability framework demonstrates that the TCN-LSTM model trained on ESM embeddings captures sequence features statistically associated with antioxidant peptide classification and long-range dependencies, providing interpretable, statistically supported explanations that generate potentially biologically meaningful hypotheses extending beyond black-box predictions.

Collectively, these results highlight that Anchor excels at identifying minimal, high-confidence motifs, LIME effectively reveals redundant and overlapping subsequences contributing to model confidence, and SHAP provides a comprehensive view of distributed contributions, including motifs with subtle contextual effects. Motifs repeatedly identified across all three methods (e.g., **RWH**, **WDD**, **YTY**, and **DED**) represent robust biochemical determinants of antioxidant potential, while features exclusive to SHAP or LIME offer opportunities for hypothesis generation and further experimental investigations.

This interpretability framework demonstrates that the TCN-LSTM model trained on ESM embeddings captures both local sequence determinants and long-range biochemical dependencies, advancing peptide-level understanding beyond traditional machine learning approaches. By combining deterministic rule-based reasoning from Anchor with LIME’s approximation and SHAP’s attributional depth, we provide a layered interpretability strategy capable of bridging black-box deep learning predictions with potentially biologically meaningful hypotheses suitable for experimental investigation.

This work demonstrates the transformative potential of XAI for advancing peptide bioinformatics, specifically, in the classification and interpretation of antioxidant peptides. By integrating an ESM with deep neural architectures combining TCNs and LSTM layers, this study moves beyond conventional sequence analysis methods, which often struggle to capture the interplay between local structural determinants and the global biochemical context. The XAI framework employed (anchored in complementary methods (Anchor, LIME, and SHAP)) positions this model not only as a high-performance predictive tool but also as a platform for hypothesis generation and mechanistic discovery.

A major implication of this work lies in its ability to identify sequence patterns that are statistically associated with antioxidant activity. Rather than offering black-box predictions, the combination of deterministic and probabilistic interpretability techniques enables researchers to pinpoint biologically relevant motifs, interpret their computational contributions, discuss their biochemical plausibility, and propose new avenues for peptide engineering. This approach creates opportunities to rationally design or optimize antioxidant peptides, accelerating innovation in diverse domains, such as nutraceuticals, dermatology, oncology, and drug discovery. For instance, the identified motifs can inform targeted peptide synthesis to enhance radical-scavenging efficiency or improve stability, bridging computational predictions with experimental design in a systematic and data-driven manner.

The applications of this work extend beyond those of antioxidant research. The methodological pipeline using embeddings from protein language models, hybrid neural architectures, and multimethod interpretability offers a general framework for analyzing bioactive peptides with complex mechanisms of action, including antimicrobial, anticancer, and immunomodulatory peptides. Furthermore, the incorporation of interpretability tools ensures regulatory transparency and enhances trust in AI-driven biomedical predictions, which are particularly relevant for clinical translation and therapeutic development. By mapping model decisions to chemical motifs, this study provides an interpretable link between machine learning outputs and hypotheses regarding potential molecular mechanisms, enabling cross-disciplinary communication between computational scientists and experimental biologists.

Nevertheless, this work has its own limitations. Although XAI methods improve transparency, their interpretations are model-dependent approximations and require careful validation through biochemical and biophysical experiments. Anchor, LIME, and SHAP highlight influential motifs but do not directly measure the mechanistic causality. Laboratory validation is essential to confirm whether predicted motifs directly influence the radical scavenging activity. Moreover, while the model leverages evolutionary and structural priors through ESM embeddings, its accuracy and generalizability are constrained by the diversity and quality of training data sets. Peptide classes or motifs underrepresented in the training data may be overlooked or mischaracterized, limiting extrapolation to novel peptide families. Additionally, the focus on *3-mer* motifs, while interpretable, may obscure higher-order structural features that contribute to function such as long-range electrostatic interactions or secondary structure elements.

### How is the Model’s Ability to Predict *6-mer* Motifs?

We also used this questioning to inquire about other aspects that are important for inferring how good the models are. Four tests were conducted with three distinct data set configurations: (i) the original data set (686 training/228 validation sequences), (ii) the fully augmented data set (32 000 training/8 000 validation sequences), and (iii) the augmented data set with controlled splitting (32 000 training/8 000 validation sequences). The methodology for all of them is further explained in the Supporting Information.

The first analysis assessed data leakage, redundancies at the sequence level, and motif overlap between the training and the validation partitions. The partitions were screened for exact duplicates, CD-HIT cluster leakage, shared *k-mers*, semantic leakage, and cross-nearest-neighbor leakage. Table S1 shows the results and highlights a critical challenge in peptide machine learning workflows: data augmentation, while beneficial for model generalization, can substantially increase multiple forms of leakage, including sequence redundancy, motif overlap, and embedding similarity. Even when standard splitting procedures are applied, these leakage pathways may persist unless explicitly controlled. With this in mind, *k-mer* leakage was removed by filtering the sequences with *6-mer* motifs. The results emphasize the importance of multilevel leakage detection, combining clustering, motif analysis, and embedding-based similarity, particularly when working with high-capacity models such as ESM that are sensitive to subtle sequence correlations.

However, we agree that the exceptionally high-performance metrics warrant careful verification to exclude potential sources of performance inflation. For this reason, we performed a comprehensive data leakage analysis by using multiple complementary criteria, the results of which are summarized in Table S1. First, we confirmed that there were no exact duplicate sequences and no CD-HIT cluster overlap between the training and validation sets in any data set configuration, indicating that identical or highly similar sequences were not shared across data partitions. We further evaluated potential motif leakage by searching for shared *6-mer* motifs across training and validation data sets. Although a small number of shared motifs were initially detected in the augmented data sets, these sequences were removed prior to model training, resulting in zero residual *6-mer* leakage after filtering (Table S1). Also, there is the possibility that shared sequence motifs or semantic similarity could influence the model performance. To investigate this, we quantified semantic leakage using ESM-2 embedding cosine similarity and evaluated cross-partition nearest neighbors in the learned representation space. These analyses intentionally measure the intrinsic biological similarity rather than data leakage. Because antioxidant peptides naturally occupy a relatively constrained sequence space, biologically related peptides are expected to exhibit similar embeddings, even when no identical sequences or motifs are shared. Consequently, the observed semantic neighbors reflect the inherent continuity of the peptide sequence landscape rather than leakage introduced during data set construction.

A second analysis assessed semantic similarities, data set diversity both within and between classes, the silhouette score, and permutation tests. The results in Table S2 highlight a critical trade-off in data set design for antioxidant peptide classification. While the original data set exhibits low intraclass diversity and high interclass similarity (conditions that limit model expressiveness), the augmented data sets significantly expand diversity and reduce interclass overlap, thereby improving the geometric separability of classes. However, these benefits must be interpreted in conjunction with potential data leakage effects (as discussed in Table S1), since excessive similarity across splits can artificially inflate performance metrics. For researchers employing ESM embeddings and explainable machine learning frameworks, these findings underscore the importance of balancing diversity, separation, and independence to achieve robust and biologically meaningful models. To further characterize the data set, we quantified intraclass diversity, interclass similarity, and cluster separability (Table S2). The original data set exhibits high interclass separation, whereas the augmented data sets display substantially increased intraclass diversity together with reduced interclass similarity. Importantly, permutation tests and bootstrap confidence intervals demonstrate that these differences are statistically significant, indicating that augmentation increases the sequence diversity without collapsing the class structure. These results suggest that the augmentation procedure expands the sampling of the existing biological sequence manifold rather than generating artificial clusters that could trivially inflate the classification performance. Although data augmentation contributes to improved model robustness by increasing the diversity of the training data, we do not attribute the observed predictive performance to augmentation. Rather, we believe that the performance arises from the combined effects of contextual ESM-2 embeddings, the complementary TCN-LSTM architecture, rigorous removal of duplicate sequences and shared motifs, and careful assessment of multiple forms of potential data leakage.

A third analysis assessed the importance of the data augmentation process by evaluating the three different data set configurations after training for Accuracy, Precision, Recall, F1 score, MCC, and Cohen’s Kappa. The results in Table S3 emphasize the critical interplay between data set construction, representation learning, and model architecture. While ESM-based embeddings combined with TCN+LSTM networks can achieve high predictive performance, the validity of these results depends heavily on the independence and diversity of the training and validation data. For research applications involving antioxidant peptide discovery, it is essential to interpret such performance metrics in the context of leakage analyses and diversity assessments to ensure that the model captures genuine biochemical signals rather than artifacts of data set design. It is also important to note that a successful classification model depends on its generalization ability, a characteristic that is mainly achieved through training with a sufficiently large amount of data. In the context of this research, it is not feasible to obtain that amount; therefore, the data augmentation process was considered necessary. Figure S1 shows the training and validation loss trajectories for the three data sets. Figure S1 underscores the importance of interpreting those curves in the broader context of data set construction and representation quality. While convergence of training and validation losses is generally desirable, in this case, it must be carefully evaluated alongside diversity metrics and leakage analyses to ensure that the model is learning meaningful biochemical patterns rather than exploiting artifacts of data augmentation. Table S4 summarizes some existing models from the literature. Definitely, the performance metrics of our model are higher than those of previously published models in the literature. In addition, our model is the only one with XAI methods implemented for antioxidant peptides in the literature.

The progression from Figures S2 to S4 demonstrates a clear improvement in model performance driven by data-centric strategies. The integration of ESM-derived embeddings with a hybrid TCN+LSTM architecture proves to be effective in capturing both local structural motifs and global sequence semantics relevant to antioxidant activity. The confusion matrices provide a comprehensive evaluation framework, revealing not only classification accuracy but also the nature of prediction errors, thereby offering critical insights into the model behavior and guiding further optimization efforts in peptide function prediction.

Figures S5 and S6 provide strong evidence that the integration of ESM-based embeddings with data-centric strategies significantly enhances the structure of the learned representation space. UMAP and t-SNE together offer complementary perspectives, capturing global manifold organization and local cluster fidelity, respectively, thereby validating the effectiveness of the proposed deep learning framework in modeling the complex sequence–function relationships underlying antioxidant peptide activity. Our augmentation strategy was designed to increase the diversity of sequence representations available during model training while preserving the amino acid composition and local sequence patterns of the original antioxidant peptides. As shown in Figures S5 and S6, the UMAP and t-SNE projections demonstrate that the augmented sequences largely occupy the same embedding space as the original peptides, indicating that the augmentation does not generate artificial clusters or substantially alter the underlying data distribution. Instead, the augmented samples remain embedded within the same feature manifold learned from the original data set, suggesting that the biological characteristics captured by the protein language model are largely preserved. Nevertheless, the augmented sequences are synthetic constructs and therefore should not be interpreted as experimentally validated peptides. Consequently, although they provide additional training examples that improve the robustness of model optimization, they may not fully represent naturally occurring biological sequences. Furthermore, because augmentation modifies the sequence context, some generated peptides may contain motif combinations that are uncommon or absent in nature. For this reason, our biological interpretation is based primarily on motifs that are consistently identified across many sequences by multiple explainability methods (Anchor, LIME, and SHAP), rather than on individual augmented peptides.

Regarding the predictive performance, we agree that data augmentation contributes to the excellent classification results by increasing the effective size and diversity of the training data and reducing overfitting. However, we do not attribute the model’s performance solely to the augmentation procedure. The strong predictive capability also results from the combination of contextual ESM-2 embeddings, the complementary TCN-LSTM architecture, and the agreement among multiple interpretability approaches. Table S5 shows the ablation studies on model components using the original and augmented data sets. The ablation study for the original data set demonstrates that ESM embeddings constitute the principal source of predictive information within the framework, while the downstream modules provide complementary mechanisms for representation learning. The relatively small differences observed among all configurations indicate that the architecture is robust and that antioxidation-related information is already strongly encoded within pretrained protein language representations. Moreover, the embedding analyses presented in Figures S5 and S6 indicate that augmentation enriches the sampling of the original feature space rather than introducing a substantially different data distribution, supporting the conclusion that the model learns meaningful patterns already present in the experimental data set instead of relying exclusively on synthetic sequences. It is important to emphasize that while the data augmentation technique improves model training and generalization, independent data sets remain essential to confirm the biological significance and transferability of the identified sequence motifs.

Now, let us answer the question: How is the model’s ability to predict *6-mer* motifs? Table S6 presents a detailed comparison of local explanation methods (Anchor explanation method, LIME, and SHAP) applied to correctly classified antioxidant peptides in an external test set. These explanations are derived from a hybrid TCN + LSTM architecture operating on embeddings generated by ESM-2, thereby reflecting both sequence-level semantics and structural context encoded in the latent space. Table S6 provides a unique opportunity to assess not only the consistency of interpretability methods but also the biochemical plausibility of the identified *6-mer* motifs contributing to antioxidant activity.

A first notable observation is the systematic discrepancy in coverage behavior for Anchor explanations. With the exception of a single instance with coverage = 1.0, most anchors exhibit coverage ≈0.0 despite high precision (≥0.95). This reinforces earlier concerns that Anchor explanations in this context are often overly specific or constrained by the perturbation distribution. The text material in the Supporting Information offers a comprehensive discussion about the plausibility of Anchor explanations. In peptide sequence space, where valid perturbations are highly structured, such near-zero coverage suggests that the extracted rules are effectively instance-specific and lack generalizability. Consequently, while the reported high precision indicates local fidelity, the anchors fail to provide broadly applicable biochemical rules. This limitation is particularly critical when interpreting sequence–function relationships, as potentially biologically meaningful hypotheses are expected to recur across multiple sequences rather than being confined to isolated instances. Here in this study, we found that the Anchor explanation for *6-mers* is overly restrictive and collapses the rule space. This does not seem to happen with the Anchor explanation for 3-mers because the explanations are not too rare compared with *6-mers*.

In contrast, LIME and SHAP provide graded importance scores for multiple *6-mers*, enabling a more nuanced interpretation of the feature contributions. LIME identifies influential *6-mers* with relatively large positive weights (e.g., *NDFFQE*, *KGMLKW*, *FRSGIL*, *IACRLY*), suggesting strong local contributions to the predicted antioxidant class. SHAP, while assigning smaller magnitude values due to its additive attribution framework, often highlights overlapping motifs (e.g., *KNAAVT*, *GFWDSV*, and *LKWKND*), thereby reinforcing the relevance of specific sequence regions. Importantly, the convergence between LIME and SHAP on several *6-mers* indicates a degree of robustness in the attribution despite their fundamentally different theoretical foundations (local linear approximation versus Shapley value decomposition).

Notably, several of these motifs appear consistently across Anchor, LIME, and SHAP, albeit with different representations. For example, motifs such as *KNAAVT*, *GFWDSV*, *FRSGIL*, and *LKWKND* are identified by at least two methods, suggesting that they represent robust determinants of antioxidant activity rather than artifacts of a specific interpretability technique. This cross-method agreement is particularly important in the context of high-dimensional embeddings from ESM, where attribution can be sensitive to methodological assumptions. However, a critical limitation emerges when considering the magnitude and interpretability of SHAP values, which are often extremely small (on the order of 10^–3^). While this is expected due to normalization across features, it complicates direct comparison with LIME scores and raises questions about practical significance. Moreover, the presence of both positive and negative SHAP contributions for similar motifs (e.g., *VQYPVE* showing both positive and negative influence) suggests context-dependent effects, where the contribution of a *6-mer* depends on its sequence environment rather than acting as an independent determinant.

From a methodological perspective, the divergence between anchor and attribution-based methods (LIME/SHAP) highlights a fundamental issue: rule-based explanations struggle in highly structured, discrete domains like peptide sequences, whereas attribution methods better capture distributed representations learned by deep models. Anchors attempt to identify minimal sufficient conditions, but in the ESM embedding space, antioxidant activity is likely encoded as a distributed, nonlinear combination of multiple motifs, making simple rule extraction inherently limited.

Table S7 also presents a comprehensive comparison of local interpretability methods (Anchor explanation method, LIME, and SHAP) applied to an external testing data set composed of 31 antioxidant peptides derived from the APD6 database. The predictions are generated by a hybrid TCN + LSTM architecture leveraging contextual embeddings from ESM-2, enabling the capture of both long-range semantic dependencies and local structural patterns in peptide sequences. Table S7 provides a critical framework for assessing the consistency, robustness, and biochemical plausibility of explanation methods in a high-dimensional, structured domain.

A consistent and critical observation across all entries in Table S6 is that Anchor explanations again exhibit systematically high precision (≈0.95–1.0) but zero coverage, reinforcing earlier concerns about their practical interpretability in protein sequence space. Within the formalism of Anchors, this indicates that the extracted rules are nongeneralizable. Consequently, while Anchor *6-mers* (e.g., *LSFRAR*, *GWANTL*, *RFIYMK*, *FLPGLI*) may highlight relevant subsequences, their explanatory value is limited by the lack of statistical support and generality. In contrast, LIME and SHAP provide distributed and quantitative attributions that are more suitable for interpreting models built on ESM-2 embeddings. LIME once more identifies influential *6-mers* with relatively large positive contributions (e.g., *RARAYG*, *GFGKPR*, *FKIPSV*, *VGVGST*, *KGGKSW*, *WRKPRW*), often capturing motifs enriched in specific physicochemical properties. SHAP, although again assigning smaller magnitude contributions due to its additive formulation, consistently highlights overlapping motifs (e.g., *AYGFRG*, *TLKNVA*, *FIYMKG*, *SVLCAV*, *IGSTIF*, *WRWWPK*), thereby reinforcing their importance. The convergence between LIME and SHAP across multiple sequences suggests that these motifs represent robust determinants encoded in the learned embedding space, rather than artifacts of a specific explanation method.

Biological validation is essential for establishing the functional relevance of identified motifs. In the present study, our objective was to assess the consistency and robustness of model-derived explanations rather than to experimentally validate individual motifs. To minimize the possibility that the interpretability results reflected training artifacts, Anchor, LIME, and SHAP analyses were performed on independent validation data sets, and any repeated or redundant k-mer motifs shared between the training and validation/testing sets were removed prior to model training, resulting in zero residual k-mer leakage after filtering. Nevertheless, we acknowledge that computational interpretability alone cannot establish a causal biological function. Accordingly, it is important to emphasize that experimental approaches, such as motif substitution or deletion studies, represent important future work to further confirm the mechanistic role of the identified motifs in the antioxidant activity.

It is important to note that the superiority of the proposed TCN+LSTM architecture, coupled with ESM embeddings, is evident when compared to conventional machine learning approaches. Although traditional classifiers such as Random Forest, ExtraTrees, Support Vector Machines, Gradient Boosting, K-Nearest Neighbors, and XGBoost can achieve reasonable predictive performance using k-mer frequency representations or one-hot encoded sequences, their results remain consistently inferior to those obtained by our proposed deep learning framework for both the original and augmented data sets (see Tables S8–S11). Also, these traditional models do not generalize, and their performance is substantially lower on independent test data sets. This performance gap highlights the limitations of handcrafted or sparse sequence representations, which primarily capture local composition while failing to adequately model contextual sequence grammar, long-range residue dependencies, and higher-order semantic relationships. In contrast, ESM embeddings provide rich contextualized representations learned from millions of protein sequences, while the TCN and LSTM modules further refine these representations through the complementary modeling of local motifs and sequential dependencies. Furthermore, although data augmentation substantially improves the predictive performance of traditional classifiers like random forest models, their explainability remains intrinsically constrained by the limited biological information encoded in *k-mer* counts (see Tables S12 and S13 for LIME analysis). Feature importance analyses in these models typically identify isolated sequence fragments or individual positions, providing only a partial view of the molecular determinants underlying antioxidation properties. Conversely, the TCN+LSTM architecture operating on ESM embeddings enables a much richer and biologically meaningful interpretation of the learned patterns. Through explainable artificial intelligence approaches, such as SHAP, LIME, and Anchor analyses, the model can reveal how contextual interactions, sequence motifs, and long-range dependencies collectively contribute to antioxidation peptide predictions. Consequently, our proposed framework offers a favorable balance between predictive performance and biological interpretability, making it particularly suitable for antioxidant peptide predictions, where understanding the mechanistic basis of predictions is as important as achieving high classification accuracy.

### Sequence Context and Support for the Biological Interpretation of *6-mer* Motifs

To provide additional sequence context beyond individual residues, the interpretability analyses were extended to contiguous *6-mer* motifs. Across independent validation data sets, LIME and SHAP consistently identified overlapping *6-mer* motifs enriched in aromatic (F, W, Y), positively charged (K, R), hydrophobic (L), and cysteine-containing (C) residues, whereas Anchor identified a smaller subset of these patterns. Importantly, redundant *6-mer* motifs shared between the training and validation data sets were removed prior to model training, resulting in zero residual *k-mer* leakage after filtering. The recurrence of similar motifs across independent data sets and complementary explainability methods suggests that the model captures statistically robust sequence patterns rather than memorizing specific peptide fragments.

Several recurrent motifs, including FLPGLI, GWANTL, RFIYMK, SVLCAV, and WRKPRW, were consistently highlighted across the explainability methods. These motifs combine aromatic, hydrophobic, positively charged, and, in some cases, cysteine residues whose physicochemical properties are consistent with biochemical features previously associated with antioxidant peptides. Nevertheless, the present computational analyses establish statistical associations learned by the model rather than experimentally validated molecular mechanisms. Accordingly, these motifs should be regarded as potentially biologically meaningful candidates for future functional investigation, rather than definitive determinants of antioxidant activity.

An additional observation is the strong agreement between LIME and SHAP, which frequently identified identical or overlapping *6-mer* motifs differing by only one or two residues. This consistency reflects the contribution of contiguous sequence regions to model predictions and supports the robustness of learned sequence representations. In contrast, the more limited and rule-based explanations produced by Anchor highlight the value of combining complementary explainability approaches to obtain a more comprehensive interpretation of deep learning models applied to peptide sequence classification.

## Conclusion

This study demonstrates the power of integrating protein language models with a hybrid deep learning architecture and multimethod interpretability tools to advance antioxidant peptide classification. By using ESM-2 embeddings, which capture both evolutionary context and biochemical semantics, and combining TCN and LSTM layers to extract local and long-range sequence patterns, we achieve highly accurate predictions while providing potentially biologically meaningful explanations. The integration of Anchor, LIME, and SHAP analyses enables the identification of key sequence motifs that may contribute to model decisions, rather than focusing only on individual amino acids while neglecting the sequence context in which they occur, transforming model predictions from opaque outputs into interpretable hypotheses regarding the importance of *k-mer* motifs for peptide classification. Beyond classification performance, this approach provides a more transparent framework that may facilitate the generation of mechanistic hypotheses for the peptide design and discovery. Although the validation strategy supports the robustness of the proposed model under the conditions examined in this study, additional validation using independent data sets and experimental studies would further strengthen its applicability. Furthermore, while the proposed framework shows promise, its broader generalizability to other peptide-related tasks, including peptide therapeutics, nutraceuticals, and functional food design for oxidative-stress-related diseases, remains to be established through future investigations.

## Supplementary Material



## Data Availability

Code, input data, and processing scripts for this paper are available at a GitHub repository (https://github.com/andresilvapimentel/Antioxidant-peptide-explainer). The data analysis scripts of this paper are also available in the interactive notebook Google Colab (for running jobs on a NVIDIA A100 80GB with 9.7 TFLOPS or NVIDIA A100 40GB with 9.7 TFLOPS GPUs) or Jupyter notebook (for running jobs locally on a PC I9 14900K, PL Z790 M 64 GB Fury with a NVIDIA RTX4090 24GB GPU).

## Notes

Data leakage assessment; quantitative assessment of the intraclass diversity for antioxidant and nonantioxidant peptides; Performance statistical metrics; metrics comparison between some existing models in literature; ablation study; training and validation losses; confusion matrixes; multimodal embedding space of antioxidant peptide sequences; Anchor, Lime, and SHAP explanations; Traditional model performances using the counting k-mers and one-hot encoding methods; and LIME explanations of the random forest model for the independent test data sets 1 and 2 (PDF)

## References

[1] Jiang Y., Sun J., Chandrapala J., Majzoobi M., Brennan C., Zeng X., Sun B. (2024). Recent Progress of Food-derived Bioactive Peptides: Extraction, Purification,
Function, and Encapsulation. Food Front..

[2] Moretta A., Scieuzo C., Petrone A. M., Salvia R., Manniello M. D., Franco A., Lucchetti D., Vassallo A., Vogel H., Sgambato A., Falabella P. (2021). Antimicrobial
Peptides: A New Hope
in Biomedical and Pharmaceutical Fields. Front.
Cell. Infect. Microbiol..

[3] Wang L., Wang N., Zhang W., Cheng X., Yan Z., Shao G., Wang X., Wang R., Fu C. (2022). Therapeutic
Peptides: Current Applications and Future Directions. Signal Transduction Targeted Ther..

[4] Xu B., Wang Y., Zha L., Chen M., Yu P., Dong Q., Suwantong O., Zhao Y. (2025). Novel Antioxidant Peptides
from Hydrolyzed Proteins of Volvariella Volvacea: Purification, Characterization
and Protective Effects on H2O2-Induced Human Skin Fibroblasts. Food Biosci..

[5] Alzaydi A., Barbhuiya R. I., Routray W., Elsayed A., Singh A. (2023). Bioactive
Peptides: Synthesis, Applications, and Associated Challenges. Food Bioeng..

[6] Zhu Y., Wang K., Jia X., Fu C., Yu H., Wang Y. (2024). Antioxidant Peptides, the Guardian of Life from Oxidative Stress. Med. Res. Rev..

[7] Zhu Z., Xu Z., Li Y., Fan Y., Zhou Y., Song K., Meng L. (2024). Antioxidant Function
and Application of Plant-Derived Peptides. Antioxidants.

[8] Wang D., Xu Z., Wang Y., Li Y., Zheng W., Chai Y., Wei G., Huang A. (2025). Identification and Characterization of Novel Antioxidant
Peptides from Yunnan Dry-Cured Beef: A Combined in Silico and in Vitro
Study. Food Chem..

[9] Zhang Y., Li Y., Quan Z., Xiao P., Duan J.-A. (2024). New Insights into
Antioxidant Peptides: An Overview of Efficient Screening, Evaluation
Models, Molecular Mechanisms, and Applications. Antioxidants.

[10] Roy S., Ramakrishnan R., Afzia N., Ghosh T., Zhang W. (2024). Recent Progress
in the Antimicrobial and Antioxidant Peptide Activated Film/Coatings
for Food Packaging Applications: A Review. Food
Biosci..

[11] Wu R., Huang J., Huan R., Chen L., Yi C., Liu D., Wang M., Liu C., He H. (2021). New Insights into the
Structure-Activity Relationships of Antioxidative Peptide PMRGGGGYHY. Food Chem..

[12] Zou L., Yu X., Cai K., Xu B., Chen C., Xiao G. (2023). Identification
of Antioxidant Peptides Targeting Keap1–Nrf2–ARE Pathway
from in Vitro Digestion of Pork Sausage with Partial Substitution
of NaCl by KCl. Food Res. Int..

[13] Veiga E., Ferreira L., Correia M., Pires P. C., Hameed H., Araújo A. R. T. S., Cefali L. C., Mazzola P. G., Hamishehkar H., Veiga F., Paiva-Santos A. C. (2023). Anti-Aging
Peptides for Advanced Skincare: Focus on Nanodelivery Systems. J. Drug Delivery Sci. Technol..

[14] R K., Kumar A., Vinod Kumar K., Sengupta A., Kundal K., Sharma S., Pawar A., Krishna P. S., Alfatah M., Ray S., Tiwari B., Kumar R. (2024). AagingBase: A Comprehensive Database
of Anti-Aging Peptides.. Database.

[15] Zhu M., Meng P., Ling X., Zhou L. (2020). Advancements in Therapeutic
Drugs Targeting of Senescence. Ther. Adv. Chronic
Dis..

[16] La
Manna S., Di Natale C., Florio D., Marasco D. (2018). Peptides as
Therapeutic Agents for Inflammatory-Related Diseases. Int. J. Mol. Sci..

[17] Chakrabarti S., Jahandideh F., Wu J. (2014). Food-Derived Bioactive Peptides on
Inflammation and Oxidative Stress. Biomed Res.
Int..

[18] Liu H., Zhang L., Yu J., Shao S. (2024). Advances in the Application
and Mechanism of Bioactive Peptides in the Treatment of Inflammation. Front. Immunol..

[19] Valuparampil
Varghese M., Niihori M., Eccles C. A., Kurdyukov S., James J., Rafikova O., Rafikov R. (2020). Antioxidant-Conjugated
Peptide Attenuated Metabolic Reprogramming in Pulmonary Hypertension. Antioxidants.

[20] Qiao Q., Chen L., Li X., Lu X., Xu Q. (2021). Roles of Dietary
Bioactive Peptides in Redox Balance and Metabolic Disorders. Oxid. Med. Cell. Longev..

[21] Hamadou M. (2025). Bioactive
Peptides and Metabolic Health: A Mechanistic Review of the Impact
on Insulin Sensitivity, Lipid Profiles, and Inflammation. Appl. Food Res..

[22] Ghadiri N., Javidan M., Sheikhi S., Taştan Ö., Parodi A., Liao Z., Tayybi Azar M., Ganjalıkhani-Hakemi M. (2024). Bioactive Peptides: An Alternative
Therapeutic Approach for Cancer Management. Front. Immunol..

[23] OZAWA H., MIYAZAWA T., BURDEOS G. C., MIYAZAWA T. (2022). Biological Functions
of Antioxidant Dipeptides. J. Nutr. Sci. Vitaminol.
(Tokyo)..

[24] Ohashi Y., Onuma R., Naganuma T., Ogawa T., Naude R., Nokihara K., Muramoto K. (2015). Antioxidant Properties of Tripeptides
Revealed by a Comparison of Six Different Assays. Food Sci. Technol. Res..

[25] Csire G., Canabady-Rochelle L., Averlant-Petit M.-C., Selmeczi K., Stefan L. (2020). Both Metal-Chelating
and Free Radical-Scavenging Synthetic Pentapeptides as Efficient Inhibitors
of Reactive Oxygen Species Generation. Metallomics.

[26] Xu B., Dong Q., Yu C., Chen H., Zhao Y., Zhang B., Yu P., Chen M. (2024). Advances in Research
on the Activity Evaluation, Mechanism and Structure-Activity Relationships
of Natural Antioxidant Peptides. Antioxidants.

[27] Xu F., Zhang Y., Qiu Y., Yang F., Liu G., Dong X., Chen G., Cao C., Zhang Q., Zhang S., Li B. (2022). Three Novel Antioxidant
Peptides
Isolated from C-Phycocyanin against H2O2-Induced Oxidative Stress
in Zebrafish via Nrf2 Signaling Pathway. Front.
Mar. Sci..

[28] Jie Y., Zhao H., Zhang B. (2019). The Role of
an Acidic Peptide in
Controlling the Oxidation Process of Walnut Oil. Foods.

[29] Zhu Y., Lao F., Pan X., Wu J. (2022). Food Protein-Derived Antioxidant
Peptides: Molecular Mechanism, Stability and Bioavailability. Biomolecules.

[30] Hogan S., Zhang L., Li J., Wang H., Zhou K. (2009). Development
of Antioxidant Rich Peptides from Milk Protein by Microbial Proteases
and Analysis of Their Effects on Lipid Peroxidation in Cooked Beef. Food Chem..

[31] Nwachukwu I. D., Aluko R. E. (2019). Structural and Functional
Properties of Food Protein-Derived
Antioxidant Peptides. J. Food Biochem..

[32] Pintea A., Manea A., Pintea C., Vlad R.-A., Bîrsan M., Antonoaea P., Rédai E. M., Ciurba A. (2025). Peptides: Emerging
Candidates for the Prevention and Treatment of Skin Senescence: A
Review. Biomolecules.

[33] Song Y., Wu C., Zhang X., Bian W., Liu N., Yin S., Yang M., Luo M., Tang J., Yang X. (2019). A Short Peptide
Potentially Promotes the Healing of Skin Wound. Biosci. Rep..

[34] Md
Fadilah N. I., Shahabudin N. A., Mohd Razif R. A., Sanyal A., Ghosh A., Baharin K. I., Ahmad H., Maarof M., Motta A., Fauzi M. B. (2024). Discovery of Bioactive
Peptides as Therapeutic Agents for Skin Wound Repair. J. Tissue Eng..

[35] Mistry K., van der Steen B., Clifford T., van Holthoon F., Kleinnijenhuis A., Prawitt J., Labus M., Vanhoecke B., Lovat P. E., McConnell A. (2021). Potentiating Cutaneous Wound Healing
in Young and Aged Skin with Nutraceutical Collagen Peptides. Clin. Exp. Dermatol..

[36] Liao I., He C., Liu X. L., Barresi-Thornton R., Wangari-Olivero J., Qian M. K., Neo B., Betts R., Ding C., Juchaux F., Chen Y., Marat X., Zheng Q., Bouez C. (2024). The Application of
a Multi-component Reaction Peptide as a Model
Regenerative Active to Enhance Skin Wound-healing Postlaser Procedure
in a Double-blinded Placebo-controlled Clinical Trial. J. Cosmet. Dermatol..

[37] Sakuma A., Kai Y., Yamasaki Y., Tanaka T., Sakurai T. (2024). Oral Administration
of Cysteine Peptides Attenuates UV-B-Induced Skin Erythema and Pigmentation
in Humans. Sci. Rep..

[38] Liu H., Guo X., Yi T., Zhu Y., Ren X., Guo R., Dai Y., Liang S. (2021). Frog Skin
Derived Peptides With Potential Protective
Effects on Ultraviolet B–Induced Cutaneous Photodamage. Front. Immunol..

[39] Liu Y., Wu G., Feng L., Li J., Xia Y., Guo W., Zhao K. (2025). Harnessing Antioxidants
in Cancer Therapy: Opportunities, Challenges,
and Future Directions. Antioxidants.

[40] Felício M. R., Silva O. N., Gonçalves S., Santos N. C., Franco O. L. (2017). Peptides
with Dual Antimicrobial and Anticancer Activities. Front. Chem..

[41] Zhang H., Mao Z., Kang Y., Zhang W., Mei L., Ji X. (2023). Redox Regulation
and Its Emerging Roles in Cancer Treatment. Coord. Chem. Rev..

[42] Zinovkin R. A., Lyamzaev K. G., Chernyak B. V. (2023). Current Perspectives
of Mitochondria-Targeted
Antioxidants in Cancer Prevention and Treatment. Front. Cell Dev. Biol..

[43] Jafari A., Babajani A., Sarrami
Forooshani R., Yazdani M., Rezaei-Tavirani M. (2022). Clinical Applications
and Anticancer Effects of Antimicrobial Peptides: From Bench to Bedside. Front. Oncol..

[44] Chi C.-F., Hu F.-Y., Wang B., Li T., Ding G.-F. (2015). Antioxidant
and Anticancer Peptides from the Protein Hydrolysate of Blood Clam
(Tegillarca Granosa) Muscle. J. Funct. Foods.

[45] Petsantad P., Sangtanoo P., Srimongkol P., Saisavoey T., Reamtong O., Chaitanawisuti N., Karnchanatat A. (2020). The Antioxidant
Potential of Peptides Obtained from the Spotted Babylon Snail (*Babylonia Areolata*) in Treating Human Colon Adenocarcinoma
(Caco-2) Cells. RSC Adv..

[46] Xie M., Liu D., Yang Y. (2020). Anti-Cancer
Peptides: Classification, Mechanism of
Action, Reconstruction and Modification. Open
Biol..

[47] Du Z., Wang D., Li Y. (2022). Comprehensive
Evaluation and Comparison
of Machine Learning Methods in QSAR Modeling of Antioxidant Tripeptides. ACS Omega.

[48] Li R., Wang H., Yu Q., Cai J., Jiang L., Luo X., Zou Q., Lv Z. (2025). AOPxSVM: A
Support Vector Machine
for Identifying Antioxidant Peptides Using a Block Substitution Matrix
and Amino Acid Composition, Transformation, and Distribution Embeddings. Foods.

[49] Li Y.-W., Li B. (2013). Characterization of Structure–Antioxidant Activity Relationship
of Peptides in Free Radical Systems Using QSAR Models: Key Sequence
Positions and Their Amino Acid Properties. J.
Theor. Biol..

[50] Shen Y., Liu C., Chi K., Gao Q., Bai X., Xu Y., Guo N. (2022). Development of a Machine Learning-Based Predictor for Identifying
and Discovering Antioxidant Peptides Based on a New Strategy. Food Control.

[51] Li W., Liu X., Liu Y., Zheng Z. (2025). High-Accuracy Identification and
Structure–Activity Analysis of Antioxidant Peptides via Deep
Learning and Quantum Chemistry. J. Chem. Inf.
Model..

[52] Qin D., Jiao L., Wang R., Zhao Y., Hao Y., Liang G. (2023). Prediction of Antioxidant Peptides Using a Quantitative Structure–activity
Relationship Predictor (AnOxPP) Based on Bidirectional Long Short-Term
Memory Neural Network and Interpretable Amino Acid Descriptors. Comput. Biol. Med..

[53] Olsen T. H., Yesiltas B., Marin F. I., Pertseva M., García-Moreno P. J., Gregersen S., Overgaard M. T., Jacobsen C., Lund O., Hansen E. B., Marcatili P. (2020). AnOxPePred: Using Deep Learning for
the Prediction of Antioxidative Properties of Peptides. Sci. Rep..

[54] Jiang Y., Wang R., Feng J., Jin J., Liang S., Li Z., Yu Y., Ma A., Su R., Zou Q., Ma Q., Wei L. (2023). Explainable Deep Hypergraph
Learning Modeling the Peptide
Secondary Structure Prediction. Adv. Sci..

[55] Zhai Y., Chen Y., Teng Z., Zhao Y. (2020). Identifying Antioxidant
Proteins by Using Amino Acid Composition and Protein-Protein Interactions. Front. Cell Dev. Biol..

[56] Li Y., Li B., He J., Qian P. (2011). Structure–Activity Relationship
Study of Antioxidative Peptides by QSAR Modeling: The Amino Acid next
to *C* -terminus Affects the Activity. J. Pept. Sci..

[57] Jiang W., Zhu Z., Pan F., Zhao L., Zhao L. (2025). Machine Learning-Driven
Screening of Antioxidant Peptides from Macadamia Nuts: In Vitro Experimental
Validation and Mechanistic Insights. Food Res.
Int..

[58] Li J., Xiong S., Shi H., Cui F., Zhang Z., Wei L. (2025). NeuroPred-AIMP: Multimodal Deep Learning
for Neuropeptide Prediction
via Protein Language Modeling and Temporal Convolutional Networks. J. Chem. Inf. Model..

[59] Singh V., Singh S. K. (2023). A Separable Temporal
Convolutional Networks Based Deep
Learning Technique for Discovering Antiviral Medicines. Sci. Rep..

[60] Yi H.-C., You Z.-H., Zhou X., Cheng L., Li X., Jiang T.-H., Chen Z.-H. (2019). ACP-DL: A Deep Learning Long Short-Term
Memory Model to Predict Anticancer Peptides Using High-Efficiency
Feature Representation. Mol. Ther. Nucleic Acids.

[61] Ribeiro, M. T. ; Singh, S. ; Guestrin, C. Anchors: High-Precision Model-Agnostic Explanations, Proceedings of the AAAI Conference on Artificial Intelligence. Vol. 321; 2018 10.1609/aaai.v32i1.11491.

[62] Ribeiro, M. T. ; Singh, S. ; Guestrin, C. “Why Should I Trust You?”: Explaining the Predictions of Any Classifier 2016, arXiv:1602.04938. arXiv.org e-Print archive. https://arxiv.org/abs/1602.04938 (accessed Dec 27, 2023).

[63] Štrumbelj E., Kononenko I. (2014). Explaining Prediction Models and Individual Predictions
with Feature Contributions. Knowl. Inf. Syst..

[64] Wang G., Schmidt C., Li X., Wang Z. (2026). APD6: The Antimicrobial
Peptide Database Is Expanded to Promote Research and Development by
Deploying an Unprecedented Information Pipeline. Nucleic Acids Res..

[65] Lin Z., Akin H., Rao R., Hie B., Zhu Z., Lu W., Smetanin N., Verkuil R., Kabeli O., Shmueli Y., dos Santos Costa A., Fazel-Zarandi M., Sercu T., Candido S., Rives A. (2023). Evolutionary-Scale
Prediction of Atomic-Level Protein Structure with
a Language Model. Science (1979)..

[66] Hie, B. ; Candido, S. ; Lin, Z. ; Kabeli, O. ; Rao, R. ; Smetanin, N. ; Sercu, T. ; Rives, A. A High-Level Programming Language for Generative Protein Design. 2022 10.1101/2022.12.21.521526.

[67] Rives A., Meier J., Sercu T., Goyal S., Lin Z., Liu J., Guo D., Ott M., Zitnick C. L., Ma J., Fergus R. (2021). Biological Structure
and Function Emerge from Scaling
Unsupervised Learning to 250 Million Protein Sequences. Proc. Natl. Acad. Sci. U. S. A..

[68] Verkuil, R. ; Kabeli, O. ; Du, Y. ; Wicky, B. I. M. ; Milles, L. F. ; Dauparas, J. ; Baker, D. ; Ovchinnikov, S. ; Sercu, T. ; Rives, A. Language Models Generalize beyond Natural Proteins. 2022 10.1101/2022.12.21.521521.

[69] Cabas-Mora G., Daza A., Soto-Garcia N., Garrido V., Alvarez D., Navarrete M., Sarmiento-Varon L., Sepulveda Yanez J. H., Davari M. D., Cadet F., Olivera-Nappa A., Uribe-Paredes R., Medina-Ortiz D. (2024). Peptipedia
v2.0: a peptide sequence
database and user-friendly web platform. A major update. Database.

