# Supplementary material for: Explainable Artificial Intelligence to Unveil Patterns of Antioxidant Peptides for Free Radical Regulation
Source: J Chem Inf Model. 2026 Sep 17;66(19):12430–47. doi: 10.1021/acs.jcim.6c01872 (PMC13663486; doi:10.1021/acs.jcim.6c01872)
Supplement: Supplementary file 1 [file ci6c01872_si_001.pdf]

Explainable Artificial Intelligence to Unveil Patterns of Antioxidant Peptides for Free Radical Regulation

Maria Carolina J. A. Schneider, Bárbara Saraiva Souza, Leonardo de Vasconcelos Ferreira, Marina Geisiely Damaso, and André Silva Pimentel*

Departamento de Química, Pontifícia Universidade Católica do Rio de Janeiro, Rio de Janeiro, RJ 22453-900 Brazil

*Corresponding author: a_pimentel@puc-rio.br

Supporting Information

Data Leakage Assessment

Table S1 shows a comprehensive data leakage assessment between training and validation splits across three distinct dataset configurations for antioxidant peptide classification: (i) the original dataset (686 training / 228 validation sequences), (ii) the fully augmented dataset (32,000 training / 8,000 validation sequences), and (iii) the augmented dataset with controlled splitting (32,000 training / 8,000 validation sequences). The analysis integrates sequence-level redundancy, motif-level overlap, and embedding-based semantic similarity to quantify potential information leakage that could artificially inflate model performance, particularly in the context of protein language models such as ESM and downstream neural architectures.

The first layer of analysis relies on clustering using CD-HIT, implemented via the Biopython ecosystem, to identify sequence redundancy across training and validation partitions. CD-HIT groups sequences based on sequence identity thresholds, enabling the detection of highly similar or near-duplicate peptides that may exist across splits. In the original dataset, clustering reveals relatively low redundancy, reflecting the curated nature of the dataset. However, in the augmented datasets—particularly the naïvely augmented configuration—there is a substantial increase in cluster overlap between training and validation sets, indicating that augmentation procedures can inadvertently introduce homologous or trivially modified sequences across splits. This effect persists even when splitting is applied post-augmentation, although it is partially mitigated depending on the splitting strategy.

At the motif level, leakage was evaluated through k-mer analysis with k = 6, a resolution that captures short functional and structural motifs relevant to antioxidant activity. High-similarity motif pairs were identified using Jaccard similarity coefficients exceeding 0.80, providing a stringent criterion for shared local sequence patterns. The results indicate significant 6-mer motif leakage in both augmented datasets (with and without splitting), suggesting that augmentation procedures tend to preserve or replicate local sequence fragments. This is particularly problematic in peptide classification tasks, where short motifs often encode biochemical functionality. Importantly, the table notes that this leakage was subsequently addressed by filtering sequences containing

overlapping 6-mer motifs, demonstrating an effective post hoc correction strategy.

Beyond syntactic similarity, the study evaluates semantic leakage using embedding representations derived from tokenized sequences, consistent with modern protein foundation models such as ESM. Cosine similarity thresholds greater than 0.95 were used to identify semantically equivalent or highly similar sequences across splits. This analysis captures deeper relationships beyond direct sequence identity, including structural or functional similarity encoded in latent space. The findings show that semantic leakage is minimal in the original dataset but becomes pronounced in the augmented datasets, reinforcing the notion that augmentation can generate embeddings that are nearly indistinguishable across training and validation sets. This is especially critical for transformer-based models, where embedding similarity strongly correlates with predictive behavior.

To further quantify cross-split dependencies, a neighborhood-based approach was employed using the NearestNeighbors implementation from scikit-learn. By identifying k-nearest neighbors (k = 5) across training and validation sets, the analysis reveals the extent to which validation samples are embedded within the local manifold of training data. The presence of cross-split nearest neighbors indicates that validation sequences may not represent truly independent samples, thereby compromising the validity of performance metrics. This effect is again most prominent in the augmented datasets, where dense sampling of sequence space leads to overlapping neighborhoods.

From a methodological standpoint, the integration of CD-HIT clustering, k-mer Jaccard similarity, cosine similarity in embedding space, and k-nearest neighbor analysis provides a robust framework for leakage auditing. For researchers developing explainable models (e.g., SHAP analyses on embeddings or physicochemical descriptors), controlling for such leakage is essential to ensure that identified features reflect genuine biological signals rather than artifacts of dataset construction.

Table S1. Data leakage summary to the validation sequences using the original antioxidant peptide dataset (686 sequences for training and 228 sequences for validation), the augmented dataset (32000 sequences for training and 8000 sequences for validation), and for the augmented original dataset with splitting (32000 sequences for training and 8000 sequences for validation). CD-HIT (Cluster Database at High Identity with Tolerance) from Biopython library is used for clustering the sequences in both training and validation datasets by efficiently grouping similar sequences together. High similarity pairs of k-mer motifs (k = 6) were detected using Jaccard similarity greater than 0.80. The semantic leakage of training sequences to the validation sequences was calculated by using the cosine similarity (higher than 0.95) of embeddings obtained from tokens of each sequence. The pairs of sequences with cross-split k-nearest neighbors (k = 5) were found using the NearestNeighbors library from sklearn package.

Dataset	Exact duplicates	CD-HIT cluster leakage	k-mer leakage (k = 6)	Semantic leakage	Cross-nearest-neighbor leakage
Original	0	0	0	181	631
Augmented	0	0	255 (0)*	7989	26838
Augmented original with splitting	0	0	174 (0)*	7986	27060

* There was 6-mer leakage in the augmented and augmented original dataset with and without splitting. This leakage was removed filtering the sequences with 6-mers motifs.

Quantitative assessment of the intra-class diversity for antioxidant and non-antioxidant peptides

Table S2 presents a quantitative assessment of the intra-class diversity for antioxidant (Class 1) and non-antioxidant peptides (Class 0), the inter-class similarity, and the Silhouette score across three dataset configurations: the original dataset, the augmented dataset, and the augmented dataset with controlled splitting. This table provides a multi-level characterization of the peptide representation space, offering insights into how data augmentation and dataset construction strategies influence the geometric structure of feature space, which is critical for the performance and interpretability of advanced neural networks and protein foundation models such as ESM.

The intra-class diversity metrics reveal notable differences between the original and augmented datasets. In the original dataset, intra-class diversity is relatively low for both Class 0 (0.0978) and Class 1 (0.105), indicating a compact representation of peptide sequences within each class. This compactness suggests that the original dataset occupies a constrained region of sequence and embedding space, likely reflecting limited sampling of the biochemical variability underlying antioxidant activity. In contrast, the augmented dataset exhibits a dramatic increase in intra-class diversity (0.639 for Class 0 and 0.598 for Class 1), demonstrating that augmentation strategies significantly expand the explored sequence space. A similar trend is observed in the augmented dataset with splitting (0.574 and 0.539 for Classes 0 and 1, respectively), albeit with slightly reduced diversity compared to the fully augmented dataset, suggesting that splitting constraints partially limit the diversity expansion.

The robustness of these diversity estimates is supported by bootstrap confidence intervals (95% CI), which are narrow in all cases, reflecting stable estimates. Importantly, the non-overlapping bootstrap CIs between Class 0 and Class 1 in each dataset configuration indicate statistically robust differences in intra-class diversity. For example, in the augmented dataset, Class 0 (0.6381–0.6405) and Class 1 (0.5964–0.5993) show clearly separated intervals, confirming that the observed diversity differences are not due to sampling variability but reflect intrinsic structural differences between the classes. This has important implications for

model training, as it suggests that each class occupies distinct regions of feature space with differing internal variability, which may influence class separability and decision boundary complexity.

The inter-class similarity provides a complementary perspective on dataset structure. In the original dataset, inter-class similarity is high (0.887), indicating that antioxidant and non-antioxidant peptides are closely positioned in feature space, which can hinder classification performance and reduce model discriminability. This high similarity suggests a poorly separated latent space, where embeddings from models such as ESM may struggle to disentangle functional signals from noise. In contrast, the augmented dataset exhibits a substantial reduction in inter-class similarity (0.257), and the augmented dataset with splitting shows a similar trend (0.303). This decrease reflects improved separation between classes, supporting the interpretation that augmentation enhances the coverage of sequence space in a way that better delineates antioxidant functionality. Notably, low inter-class similarity corresponds to a more distinct antioxidant space, which is desirable for robust classification.

The Silhouette score further quantifies the quality of clustering and class separation. The original dataset shows a near-zero Silhouette score (0.0436), indicating weak clustering structure and poor separation between classes. In the augmented dataset, the Silhouette score increases to 0.089, and further to 0.108 in the augmented dataset with splitting, reflecting improved clustering and more coherent class structure. Although these values remain modest, the relative increase demonstrates that augmentation contributes to a more structured embedding space, which can enhance the learning dynamics of deep neural networks. From the perspective of representation learning, this suggests that augmented datasets may better align with the inductive biases of transformer-based architectures, enabling more effective discrimination of functional peptide classes.

Statistical significance of the observed patterns is evaluated permutation testing (n = 1000), where class labels are randomly shuffled to generate a null distribution of the test statistic. The resulting p-values (0.007 for the original dataset and effectively 0.000 for both augmented configurations) indicate that the observed differences in diversity and similarity are highly unlikely to arise by

chance. This reinforces the conclusion that augmentation fundamentally alters the statistical structure of the dataset. The negative permutation statistics reported for augmented datasets further suggest a strong deviation from the null hypothesis, consistent with enhanced class separability and structured diversity.

Table S2. The intra-class diversity for classes 0 and 1, the inter-class similarity, and the Silhouette score for antioxidant peptide datasets. Low inter-class diversity means distinct antioxidant space. The permutation test (with p-value) evaluates the significance of a hypothesis by shuffling group labels to simulate a large number of rearrangements (n = 1000) of the observed data. The bootstrap confidence intervals (CI) for intra-class diversity are used to calculate the robustness of diversity difference. Non-overlapping bootstrap CIs means a statistically robust diversity difference.

Dataset	Intra-class diversity (Class 0)	Intra-class diversity (Class 1)	Inter-class similarity	Silhouette score	Permutation test (p-value)	Diversity 95% CI (Class 0)	Diversity 95% CI (Class 1)
Original	0.0978	0.105	0.887	0.0436	0.007 (0.039)	0.0928 0.1017	0.1013 0.1082
Augmented	0.639	0.598	0.257	0.089	-0.0414 (0.000)	0.6381 0.6405	0.5964 0.5993
Augmented original with splitting	0.574	0.539	0.303	0.108	-0.0348 (0.000)	0.5712 0.5765	0.5360 0.5423

The performance statistical metrics

Table S3 shows the performance statistical metrics (accuracy, precision, recall, F1 score, Matthews correlation coefficient (MCC), and Cohen's kappa) for both training (upper rows) and validation (lower rows) across the original, augmented, and augmented-with-splitting antioxidant peptide datasets. These results reflect the optimized performance of a hybrid deep learning architecture combining Temporal Convolutional Networks (TCN) and Long Short-Term Memory (LSTM) layers, leveraging embeddings generated by the ESM-2 model to capture both global semantic relationships and local structural dependencies in peptide sequences.

In the original dataset, the model achieves strong but not saturated performance during training, with accuracy (0.881), precision (0.803), recall (0.888), and F1 score (0.853), alongside a high MCC (0.964) and Cohen's kappa (0.891). These values indicate that the model is capable of learning meaningful discriminative patterns from limited data, particularly benefiting from the rich contextual embeddings provided by ESM. However, a substantial drop is observed in validation performance, where accuracy increases to 0.924 but precision (0.871), recall (0.663), and F1 score (0.455) decrease markedly, accompanied by lower MCC (0.652) and kappa (0.454). This divergence suggests instability in generalization, likely driven by class imbalance, limited dataset size, and insufficient coverage of the peptide sequence space. The discrepancy between recall and precision further indicates that the model may struggle to consistently identify antioxidant peptides, potentially overfitting to dominant patterns in the training data while failing to generalize to underrepresented motifs.

In contrast, the augmented dataset exhibits near-perfect performance across all metrics in both training and validation phases. Training metrics approach unity (accuracy = 0.999, precision = 0.998, recall = 0.999, F1 = 0.999, MCC = 0.999, kappa = 0.997), and validation metrics mirror this behavior with similarly extreme values (e.g., accuracy = 0.999, F1 = 0.996, MCC = 0.999). While these results may initially suggest exceptional model generalization, such uniformly high performance across all metrics raises concerns regarding potential data leakage and redundancy within the dataset. When considered alongside the leakage analysis (Table S1) and

diversity metrics (Table S2), these results strongly imply that the augmented dataset contains highly similar or semantically overlapping sequences across training and validation splits, enabling the model to effectively “memorize” patterns rather than learn generalizable biochemical rules. In particular, the extremely high MCC and kappa values—metrics that are typically robust to class imbalance—reinforce the likelihood of artificially inflated performance.

The augmented dataset with splitting presents a similar pattern, with all performance metrics effectively reaching unity in both training and validation (accuracy, precision, recall, F1, MCC, and kappa ≈ 0.999–1.000). Despite the implementation of splitting strategies intended to reduce overlap between datasets, the persistence of near-perfect validation performance suggests that residual redundancy and semantic similarity remain significant. Given the sensitivity of ESM embeddings to subtle sequence similarities, even small overlaps in sequence or motif space can lead to highly correlated representations, which are easily exploited by the TCN+LSTM architecture. This is particularly relevant for transformer-derived embeddings, where cosine similarity in latent space can capture functional similarity even in the absence of exact sequence identity.

From a modeling perspective, the integration of TCN and LSTM layers provides a powerful mechanism for capturing both short-range motif patterns and long-range dependencies in peptide sequences. The TCN component effectively models local convolutional features, while the LSTM captures sequential dependencies and contextual ordering. When combined with ESM embeddings, this architecture is theoretically well-suited for peptide classification tasks. However, the results in Table S3 demonstrate that model performance is highly contingent on dataset properties. In the presence of limited diversity (original dataset), the model exhibits moderate generalization with clear limitations. Conversely, in highly augmented datasets, performance metrics become saturated, obscuring the true predictive capability of the model.

The inclusion of MCC and Cohen’s kappa provides additional insight into model reliability. In the original dataset, the decline in these metrics during validation indicates reduced agreement beyond chance and highlights the challenge of achieving robust

classification in low-data regimes. In contrast, their near-perfect values in augmented datasets suggest complete agreement, which, while desirable in principle, is statistically improbable in realistic biological classification tasks unless the data distributions are highly overlapping or redundant.

Table S3. Performance statistical metrics (accuracy, precision, recall, F1 score, Matthews correlation coefficient (MCC), and Cohen´s kappa) of training (upper) and validation (lower) of the original, augmented, and augmented original antioxidant peptide datasets after optimization of the deep learning model (TCN + LSTM) to classify antioxidant peptides using an Evolutionary Scale Modeling (ESM) language model (esm2_t6_8M_UR50D) to capture both global semantic and local structural features.

Dataset	Accuracy	Precision	Recall	F1 score	MCC	Cohen's Kappa
Original	0.881<br>0.803	0.888<br>0.853	0.964<br>0.891	0.924<br>0.871	0.663<br>0.455	0.652<br>0.454
Augmented	0.999<br>0.998	0.999<br>0.999	0.999<br>0.997	0.999<br>0.998	0.999<br>0.996	0.999<br>0.996
Augmented original with splitting	0.999<br>0.999	0.999<br>0.999	0.999<br>0.999	0.999<br>0.999	0.999<br>0.998	0.999<br>0.998

Table S4. Metrics comparison between some existing models in literature.

Model	Accuracy	Precision	Recall	Specifity	MCC	ROC-AUC	XAI
PseAAC (1)	0.877	0.947	0.916	0.832	0.753	0.939	No
AOPP (2)	0.927	0.985	0.867	0.987	0.859	-	No
AnOxPP (3)	0.880	0.861	0.906	0.853	0.761	-	No
AnOxPePred (4)	0.753	0.826	0.810	0.627	0.433	0.778	No
TCN+LSTM (Our model)	0.999	0.999	0.999	-	0.998	-	Yes

References:

(1) PseAAC-dipeptide model: Shen et al. Food Control 2022, 131, 108439. https://doi.org/10.1016/j.foodcont.2021.108439

(2) AOPP: Li et. al J. Chem. Inf. Model. 2025, 65, 2, 603–612. https://doi.org/10.1021/acs.jcim.4c01713

(3) AnOxPP: Qin et al. Comput. Biol. Med. 2023, 154, 106591. https://doi.org/10.1016/j.compbiomed.2023.106591

(4) AnOxPePred: Olsen et al. Sci Rep 2020, 10, 21471. https://doi.org/10.1038/s41598-020-78319-w

Ablation Study

A comprehensive ablation analysis was performed using the original and augmented datasets, systematically evaluating five distinct configurations: (1) the complete hybrid model, (2) a model without the Temporal Convolutional Network (No TCN), (3) a model without the LSTM module (No LSTM), (4) a model without the feature fusion strategy (No Fusion), and (5) a baseline model relying exclusively on ESM embeddings without downstream sequential integration (ESM only). The results are summarized in Table S5.

An important observation emerging from the ablation study is that the proposed hybrid architecture should not be interpreted merely as an additive collection of independent modules designed exclusively to maximize predictive metrics. Instead, the architecture was conceived to address fundamental representational challenges commonly encountered in peptide sequence analysis, including the modeling of contextual sequence grammar, local compositional motifs, and long-range residue interactions. By integrating ESM embeddings with TCN-based local feature extraction, LSTM-based contextual dependency modeling, and feature fusion mechanisms, the framework seeks to construct a richer hierarchical representation of peptide semantics.

Interestingly, all evaluated configurations achieved comparable predictive performance. On the original dataset, accuracy values ranged from 0.788 to 0.821, F1-scores from 0.855 to 0.883, and MCC values from 0.452 to 0.499. Notably, some ablated configurations, particularly the No Fusion and No TCN models, achieved slightly higher values for several conventional classification metrics than the complete architecture. These findings indicate that the predictive information learned from ESM embeddings is already highly discriminative and that the contribution of the additional modules is not necessarily reflected through large gains in benchmark metrics alone. Rather, the proposed architecture aims to provide a more comprehensive representational framework capable of capturing multiple biological scales of information simultaneously.

The strong performance observed for the ESM-only configuration further confirms the remarkable representational capacity of modern protein language models. Despite the absence of dedicated sequential modeling layers, the ESM-only model achieved an accuracy of 0.799, an F1-score of 0.867, and an MCC of 0.452, demonstrating that pretrained protein language representations already encode substantial biochemical, structural, and evolutionary information relevant to antioxidation peptide prediction. These results support growing evidence that protein language models capture biologically meaningful sequence features even before task-specific refinement.

The removal of the TCN module produced a slight increase in performance, yielding an MCC of 0.487 compared with 0.464 for the full model. Although this result suggests that local convolutional refinement was not essential for maximizing predictive performance under the current dataset conditions, it should not be interpreted as evidence that local sequence patterns are biologically irrelevant. TCN layers remain theoretically attractive because they are specifically designed to capture motif-like patterns and local residue arrangements through dilated convolutions operating across multiple receptive field scales. The observed behavior likely reflects the fact that such local contextual information is already partially encoded within the pretrained ESM embeddings.

Similarly, removing the LSTM component resulted in a modest improvement relative to the complete architecture, producing an MCC of 0.475 and maintaining excellent accuracy, precision, and recall values. This finding suggests that the contextual information captured by ESM embeddings may already encode a substantial fraction of the long-range dependencies traditionally modeled through recurrent architectures. Nevertheless, the conceptual role of the LSTM module remains relevant because antioxidation peptide activity can depend on non-local residue interactions and contextual sequence effects. Consequently, the absence of substantial performance gains should not be interpreted as a lack of biological relevance, but rather as evidence that pretrained protein language models already provide highly contextualized representations.

The No Fusion configuration achieved the highest performance among all evaluated variants, reaching an accuracy of 0.821, an F1-score of 0.883, an MCC of 0.499, and a Cohen's Kappa of 0.496. This result indicates that explicit feature fusion was not required to maximize predictive performance on the current validation dataset. However, feature fusion remains conceptually valuable because it provides a unified latent space capable of integrating information originating from different representational perspectives, including pretrained semantic embeddings, local compositional patterns, and contextual sequence dependencies. The limited differences among the evaluated models suggest that these information sources may exhibit substantial redundancy, particularly when powerful pretrained embeddings are used as the primary sequence representation.

For the augmented validation dataset, the ablation study revealed a markedly different behavior compared to the original dataset, with all model configurations achieving near-perfect performance. Accuracy values ranged from 0.977 to 0.999, while F1-scores varied between 0.976 and 0.998. The complete hybrid model achieved the highest overall performance, reaching 0.995 accuracy, 0.999 precision, 0.990 recall, 0.995 F1-score, 0.990 MCC, and 0.990 Cohen's Kappa. Removal of individual components produced only marginal reductions in performance, indicating that the predictive signal became substantially easier to learn after data

augmentation. Notably, even the ESM-only model maintained excellent performance (accuracy = 0.977, F1-score = 0.976, MCC = 0.953), confirming the strong representational capacity of pretrained protein language embeddings. However, the consistently superior results obtained by the complete architecture suggest that the TCN, LSTM, and feature fusion modules provide complementary information that becomes more effectively exploited when sufficient training examples are available. The small but systematic performance decreases observed in the ablated configurations support the notion that local motif extraction, contextual dependency modeling, and multi-representation integration contribute synergistically to antioxidation prediction.

In summary, the ablation study for the original dataset demonstrates that ESM embeddings constitute the principal source of predictive information within the framework, while the downstream modules provide complementary mechanisms for representation learning. The relatively small differences observed among all configurations indicate that the architecture is robust and that antioxidation-related information is already strongly encoded within the pretrained protein language representations. Overall, the augmented dataset results demonstrate that the proposed hybrid architecture is capable of fully leveraging the additional diversity introduced through data augmentation, yielding highly robust and balanced predictions while preserving the biological interpretability afforded by its multiple representational components. Therefore, the value of the proposed framework should be interpreted not exclusively through incremental improvements in conventional performance metrics, but also through its ability to provide a biologically motivated and theoretically grounded representational strategy for modeling antioxidation property of peptides. Such considerations are particularly relevant in explainable artificial intelligence applications, where representational coherence and biological interpretability are often as important as marginal improvements in predictive accuracy.

Table S5. Ablation studies on model components using the original (upper row) and augmented (lower row) datasets.

Model	Accuracy	Precision	Recall	F1-score	MCC	Cohen's Kappa
Full Model	0.788<br>0.995	0.878<br>0.999	0.833<br>0.990	0.855<br>0.995	0.464<br>0.990	0.462<br>0.990
No TCN	0.815<br>0.990	0.861<br>0.989	0.898<br>0.990	0.879<br>0.990	0.487<br>0.980	0.485<br>0.980
No LSTM	0.810<br>0.998	0.860<br>0.999	0.891<br>0.996	0.875<br>0.998	0.475<br>0.995	0.474<br>0.995
No Fusion	0.821<br>0.982	0.862<br>0.986	0.906<br>0.977	0.883<br>0.981	0.499<br>0.963	0.496<br>0.964
ESM only	0.799<br>0.977	0.858<br>0.978	0.877<br>0.975	0.867<br>0.976	0.452<br>0.953	0.451<br>0.954

Training and validation losses

Figure S1 presents the training (blue line) and validation (orange line) loss trajectories for a balanced antioxidant peptide classification task across three dataset configurations: (A) the original dataset (686 training / 228 validation sequences), (B) the augmented dataset (32,000 training / 8,000 validation sequences), and (C) the augmented dataset with controlled splitting (32,000 training / 8,000 validation sequences). These loss curves, evaluated over 23–49 epochs, reflect the optimization dynamics of a hybrid deep learning architecture combining Temporal Convolutional Networks (TCN) and Long Short-Term Memory (LSTM) layers, trained on embeddings derived from the ESM-2 model to encode both global semantic information and local structural patterns in peptide sequences.

In Figure S1A, corresponding to the original dataset, the training loss exhibits a gradual and consistent decrease across epochs, indicating effective learning of discriminative features despite the relatively small dataset size. However, the validation loss shows a more irregular trajectory, with fluctuations and a tendency to plateau or even increase after initial epochs. This divergence between training and validation losses is indicative of overfitting, where the model begins to specialize on the limited training data and fails to generalize effectively to unseen validation sequences. The relatively small number of samples restricts the diversity of the learned feature space, leading to instability in the optimization process. From a representation learning perspective, although ESM embeddings provide rich contextual information, the limited sampling of sequence space constrains the model’s ability to learn robust decision boundaries.

In contrast, Figure S1B, representing the fully augmented dataset, shows a markedly different behavior. Both training and validation losses decrease rapidly and converge to near-zero values within a relatively small number of epochs. The close alignment between the two curves throughout training suggests minimal generalization gap, which would typically be interpreted as strong model generalization. However, given the scale of augmentation and prior evidence of sequence and semantic redundancy, this behavior is

more consistent with an overparameterized model learning highly redundant or overlapping representations. The near-identical loss trajectories indicate that validation samples are not sufficiently independent from training data, allowing the model to generalize trivially. This effect is amplified by the expressive capacity of the TCN+LSTM architecture and the high-dimensional embeddings generated by ESM, which can easily encode subtle sequence similarities.

Figure S1C corresponds the augmented dataset with splitting and exhibits similar but slightly more nuanced dynamics. While both training and validation losses still decrease rapidly and converge to very low values, the validation loss may show marginally higher values or minor deviations compared to the training curve. This suggests that the splitting strategy introduces a modest degree of independence between training and validation sets, partially mitigating the redundancy observed in panel (B). Nevertheless, the overall convergence pattern remains indicative of a highly learnable dataset, where the model encounters little resistance in minimizing loss. The persistence of near-overlapping loss curves implies that, despite splitting, residual similarities in sequence or embedding space continue to facilitate easy generalization.

From an optimization standpoint, the observed behaviors across the three panels highlight the interplay between dataset size, diversity, and model capacity. In low-data regimes (Figure S1A), the model struggles with generalization and exhibits classical overfitting patterns. In high-data but potentially redundant regimes (Figures S1B and S1C), the optimization landscape becomes smooth and easily navigable, leading to rapid convergence but potentially misleading performance estimates. The use of ESM embeddings plays a central role in this process, as their ability to encode biochemical and evolutionary information can either enhance generalization (when data are diverse) or exacerbate leakage effects (when data are redundant).

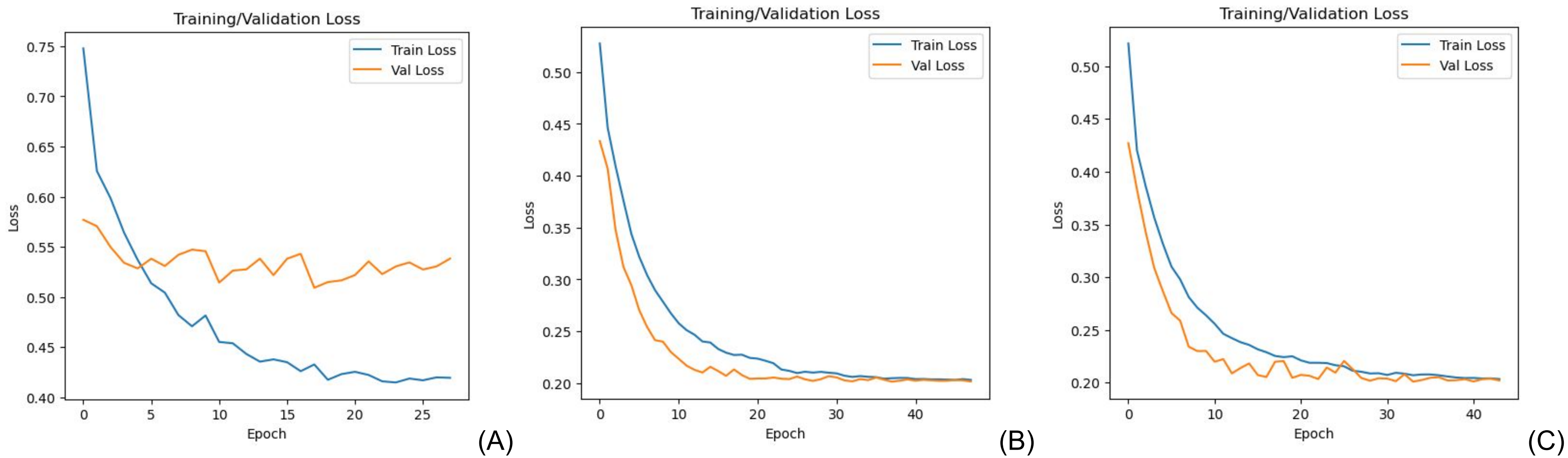

Figure S1. Training (blue line) and validation (orange line) losses for balanced antioxidant peptide dataset with 23-49 epochs during optimization of the deep learning model (TCN + LSTM) to respectively classify antioxidant peptides using an Evolutionary Scale Modeling (ESM) language model (esm2_t6_8M_UR50D) to capture both global semantic and local structural features. (A) original dataset (686 sequences for training and 228 sequences for validation), (B) augmented dataset (32000 sequences for training and 8000 sequences for validation), and (C) augmented original dataset with splitting (32000 sequences for training and 8000 sequences for validation).

Confusion matrixes

Figures S2–S4 show the confusion matrices of training (left panels, A) and validation (right panels, B) datasets obtained during the optimization of a hybrid deep learning architecture combining Temporal Convolutional Networks (TCN) and Long Short-Term Memory (LSTM) layers, leveraging embeddings derived from the Evolutionary Scale Modeling (ESM) protein language model (esm2_t6_8M_UR50D) to classify antioxidant peptides . These figures collectively illustrate the model's classification behavior under different dataset configurations (original, augmented, and augmented with splitting) and at distinct stages of training convergence (23–49 epochs), providing insight into both learning dynamics and generalization performance.

In Figure S2, which corresponds to the original dataset after 28 training epochs, the confusion matrices reveal the baseline discriminative capacity of the TCN+LSTM architecture when trained on non-augmented data. The training matrix (panel A) typically exhibits a strong diagonal dominance, indicating that the model effectively captures class-specific patterns within the training set. This performance can be attributed to the synergy between TCN layers—capable of extracting local sequence motifs through dilated causal convolutions—and LSTM units, which model long-range dependencies inherent to peptide sequences. The use of esm2_t6_8M_UR50D embeddings further enhances this capability by encoding evolutionary and contextual information at the residue level, effectively bridging sequence semantics with structural propensities. However, the validation matrix (panel B) may display a modest increase in off-diagonal elements, suggesting the presence of generalization gaps, likely due to limited data diversity and potential overfitting to the original dataset distribution.

Figure S3 presents the confusion matrices obtained after 49 epochs using augmented datasets, highlighting the impact of data augmentation strategies on model robustness. In this case, both training and validation matrices exhibit improved alignment along the diagonal, reflecting enhanced classification accuracy and reduced misclassification rates. The augmentation process likely introduces variability in sequence patterns—such as residue substitutions or synthetic peptide generation—thereby enabling the

model to learn a more generalized representation of antioxidant activity. Notably, the validation performance approaches that of the training set, indicating improved generalization and reduced overfitting. This behavior underscores the importance of dataset enrichment when training deep architectures on biologically constrained datasets, where class imbalance and limited sample size are common challenges.

Figure S4 further explores the effect of dataset splitting combined with augmentation, evaluated after 23 epochs. The confusion matrices in this configuration provide evidence of faster convergence and potentially more stable generalization behavior. Despite fewer training epochs, the model maintains a strong diagonal structure in both training and validation matrices, suggesting that the combination of augmentation and careful data partitioning enhances learning efficiency. This may be attributed to improved representation of class boundaries during training, as well as reduced information leakage between training and validation subsets. The relatively balanced distribution of true positives and true negatives across both panels indicates that the model achieves a consistent trade-off between sensitivity and specificity.

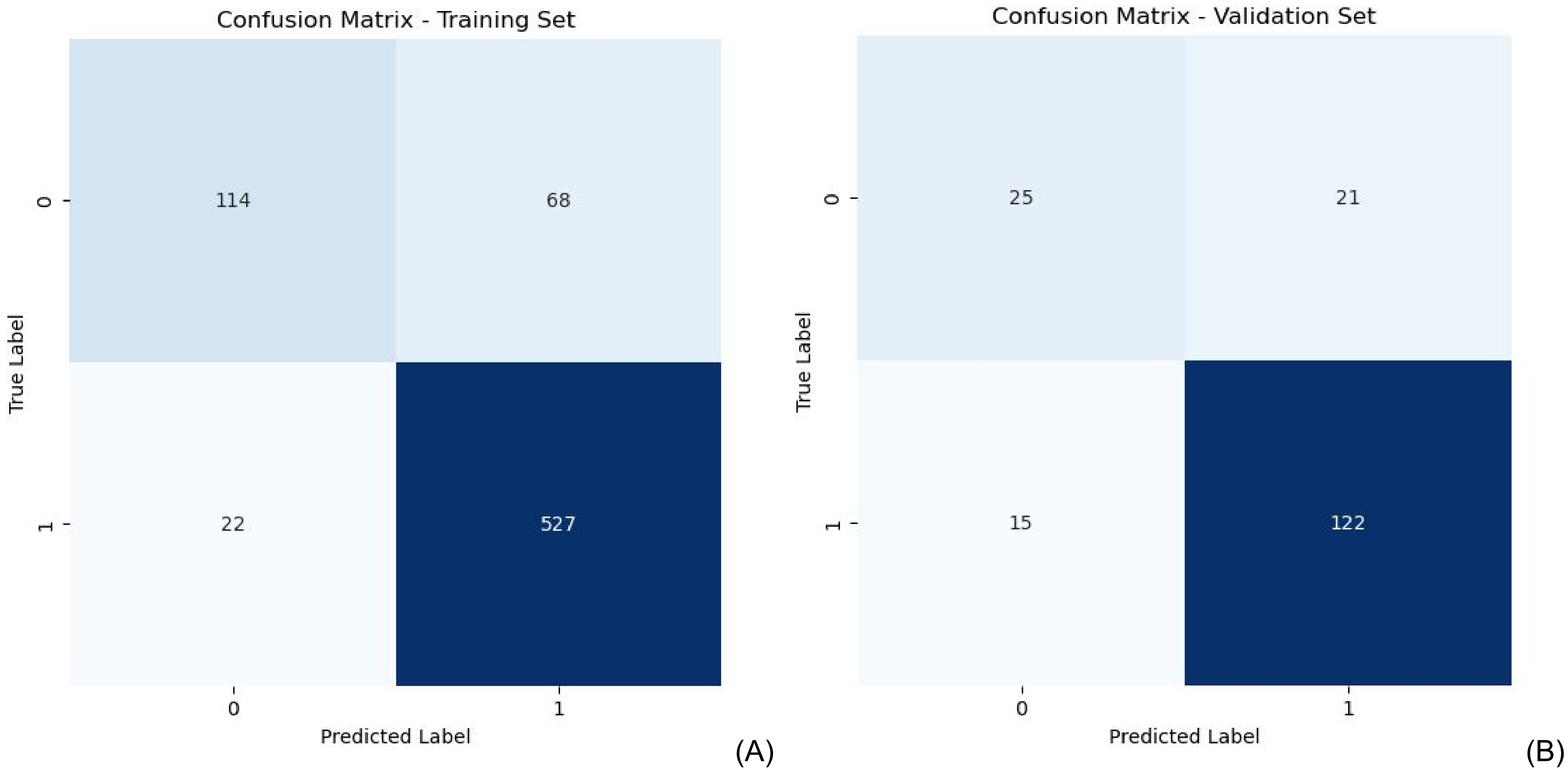

Figure S2. Confusion matrices of training (left panel, A) and validation (right panel, B) original dataset after 28 epoch optimizations of the deep learning model (TCN + LSTM) to respectively classify antioxidant peptides using an Evolutionary Scale Modeling (ESM) language model (esm2_t6_8M_UR50D) to capture both global semantic and local structural features.

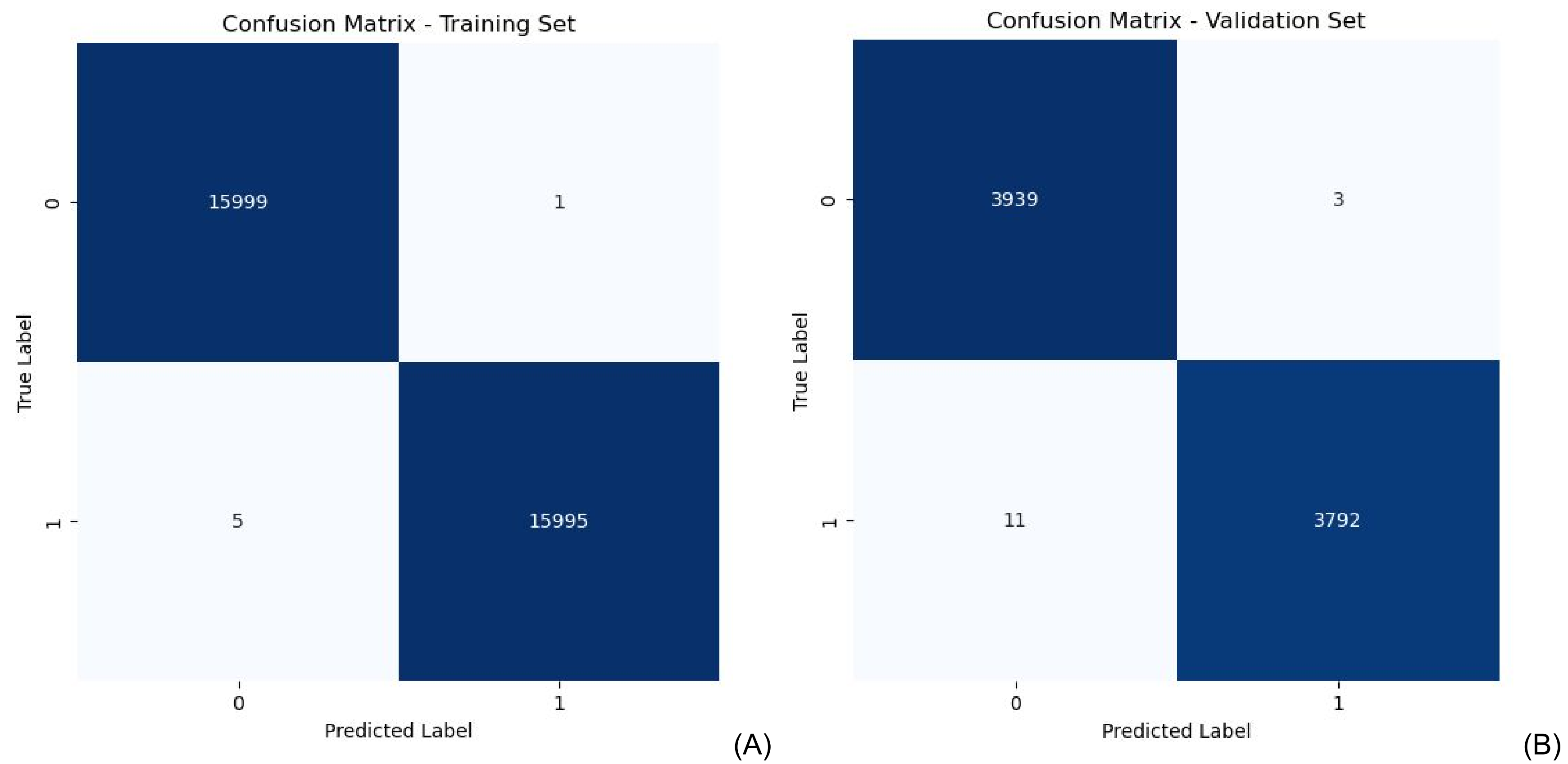

Figure S3. Confusion matrices of training (left panel, A) and validation (right panel, B) augmented datasets after 49 epoch optimizations of the deep learning model (TCN + LSTM) to respectively classify antioxidant peptides using an Evolutionary Scale Modeling (ESM) language model (esm2_t6_8M_UR50D) to capture both global semantic and local structural features.

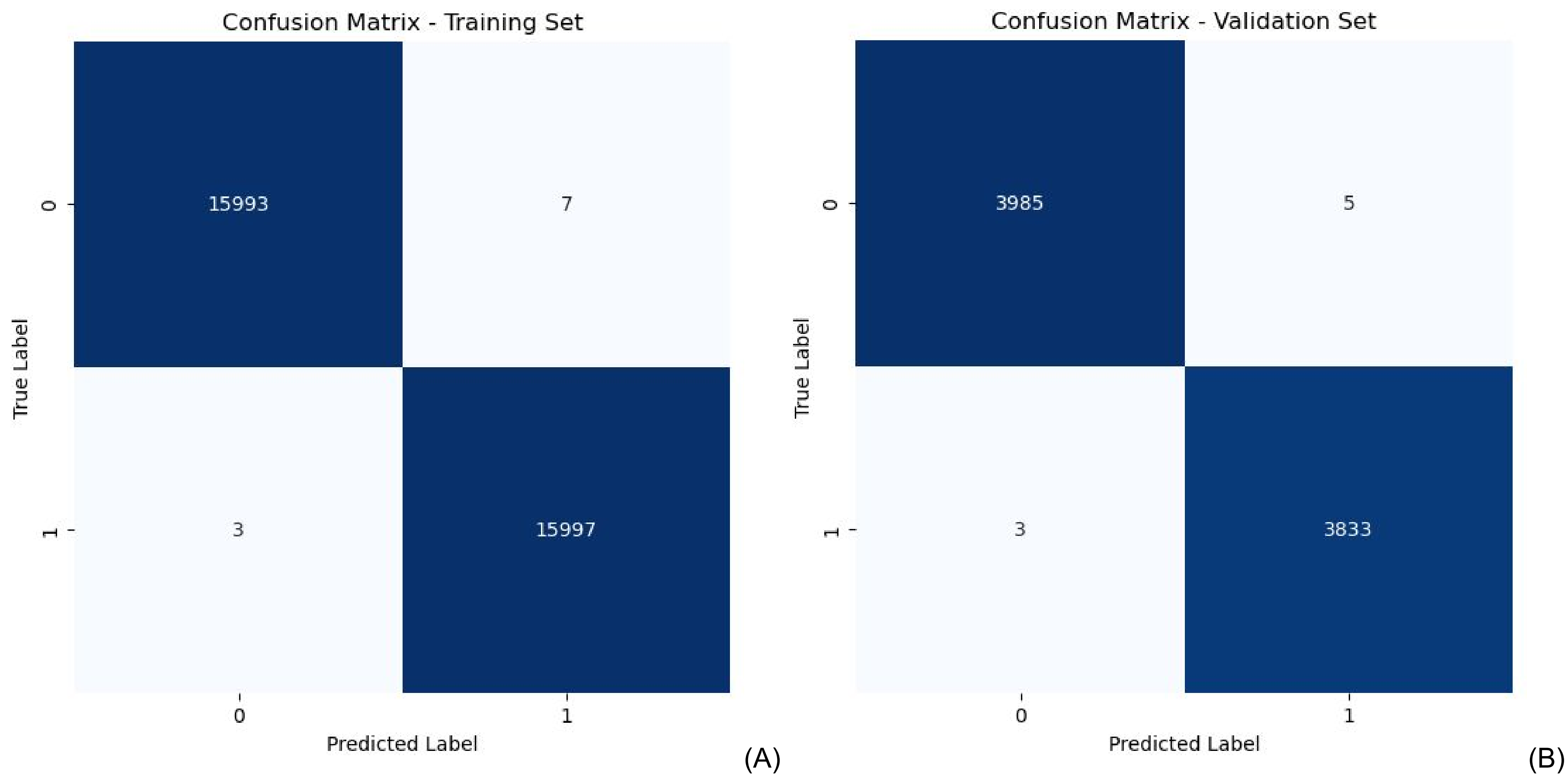

Figure S4. Confusion matrices of training (left panel, A) and validation (right panel, B) augmented original datasets with splitting after 23 epoch optimizations of the deep learning model (TCN + LSTM) to respectively classify antioxidant peptides using an Evolutionary Scale Modeling (ESM) language model (esm2_t6_8M_UR50D) to capture both global semantic and local structural features.

Multimodal embedding space of antioxidant peptide sequences

Figures S5–S6 present the multimodal embedding space of antioxidant peptide sequences projected into low-dimensional manifolds using Uniform Manifold Approximation and Projection (UMAP) and t-Distributed Stochastic Neighbor Embedding (t-SNE), respectively, for three dataset configurations: (A) the original dataset (686 training and 228 validation sequences), (B) the augmented dataset (32,000 training and 8,000 validation sequences), and (C) the augmented original dataset with splitting (32,000 training and 8,000 validation sequences) . These visualizations provide a comprehensive representation of how high-dimensional embeddings derived from the ESM protein language model (esm2_t6_8M_UR50D) organize peptide sequences in latent space, reflecting both functional similarity and structural patterns relevant to antioxidant activity.

In Figure S5, the UMAP projections highlight the global structure of the embedding space while preserving local neighborhood relationships. For the original dataset (Figure S5A), the embedding distribution appears relatively sparse and fragmented, reflecting the limited sample size and constrained diversity of peptide sequences. Although some degree of clustering may be observed between antioxidant and non-antioxidant classes, the boundaries are not sharply defined, suggesting that the model operates in a regime where class separability is partially learned but still influenced by data scarcity. The integration of ESM embeddings contributes significantly to capturing underlying biochemical and evolutionary features; however, the limited coverage of sequence space restricts the formation of well-defined manifolds.

In contrast, the augmented dataset (Figure S5B) exhibits a markedly denser and more structured embedding topology. The increased number of sequences expands the coverage of the peptide sequence space, allowing UMAP to reveal more continuous and coherent manifolds. Distinct clusters corresponding to functional classes emerge with improved separation, indicating that the augmented data enhances the representation learning process. This effect can be attributed to the ability of data augmentation to introduce controlled variability, enabling the model to generalize across sequence variants while maintaining class-discriminative

features. The resulting embedding space reflects a smoother geometry, where intra-class compactness and inter-class separation are both improved.

Figure S5C, representing the augmented dataset with splitting, further refines this structure. The embedding space demonstrates balanced clustering patterns between training and validation samples, suggesting that the data partitioning strategy preserves the statistical properties of the full dataset while minimizing distributional shift. The clusters appear more uniformly distributed, with reduced overlap between classes, which is indicative of improved generalization and reduced overfitting. This configuration likely benefits from both the richness of augmented data and the robustness introduced by careful dataset splitting, leading to a more reliable latent representation for downstream classification.

Figure S6 complements these observations using t-SNE, a technique that emphasizes local neighborhood preservation and is particularly effective at revealing fine-grained cluster structures. In panel A (original dataset), t-SNE projections often show scattered micro-clusters with limited global organization, reflecting the high sensitivity of the method to small sample sizes and local density variations. While some class-specific groupings may be visible, the lack of continuity between clusters suggests that the learned embeddings are not fully capturing the underlying functional landscape.

In Figure S6B (augmented dataset), t-SNE reveals a more intricate and well-separated clustering pattern, with clearly delineated regions corresponding to different peptide classes. The higher data density enables the algorithm to better preserve local similarities, resulting in tighter clusters and clearer boundaries. This indicates that the ESM-derived embeddings, when supported by sufficient data, encode meaningful biochemical features that are consistent across sequence variants. The clustering structure observed here is consistent with improved classification performance, as also reflected in the confusion matrices discussed previously.

Figure S6C (augmented dataset with splitting) demonstrates the most coherent and stable embedding configuration. The clusters are not only well-separated but also exhibit internal consistency, with minimal dispersion of samples within each class. Importantly, training and validation points appear to occupy similar regions of the embedding space, reinforcing the notion that the model generalizes effectively across unseen data. The combination of augmentation and proper data splitting thus leads to a latent space that is both discriminative and robust, supporting accurate classification of antioxidant peptides.

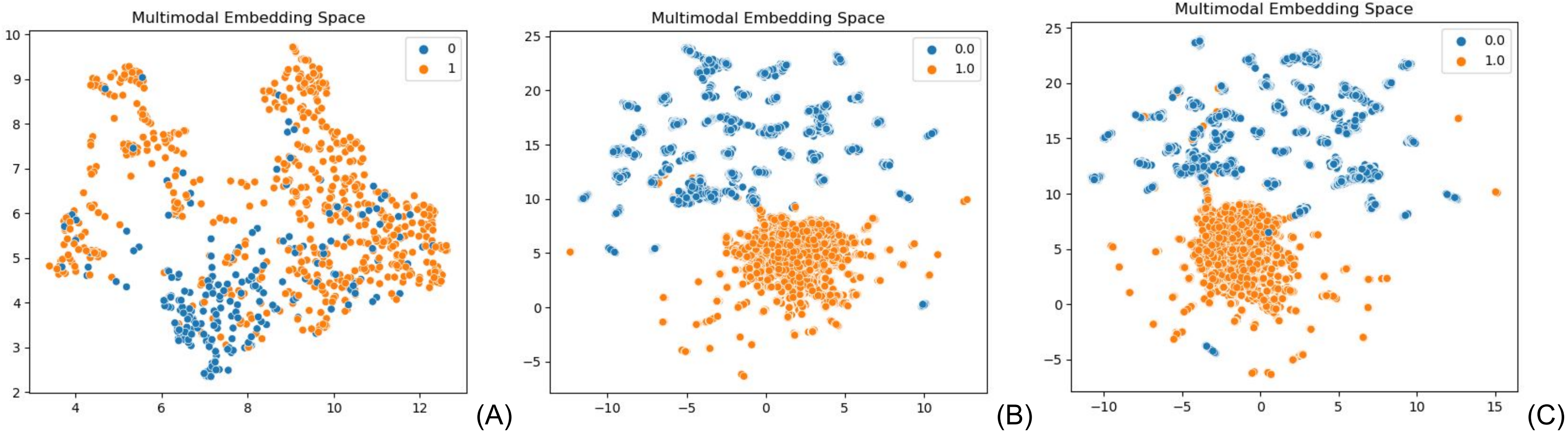

Figure S5. Multimodal embedding space for the (A) original antioxidant peptide dataset (686 sequences for training and 228 sequences for validation), (B) augmented dataset (32000 sequences for training and 8000 sequences for validation), and (C) augmented original dataset with splitting (32000 sequences for training and 8000 sequences for validation) using the Uniform Manifold Approximation (UMAP) technique.

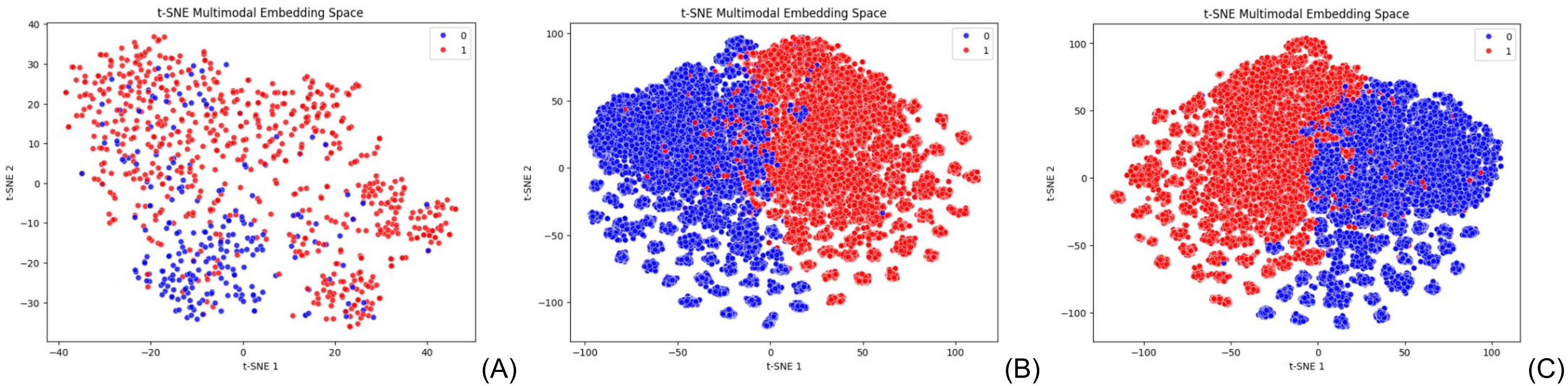

Figure S6. Multimodal embedding space for the (A) original dataset (686 sequences for training and 228 sequences for validation), (B) augmented dataset (32000 sequences for training and 8000 sequences for validation), and (C) augmented original dataset with splitting (32000 sequences for training and 8000 sequences for validation) using the t-Distributed Stochastic Neighbor Embedding (t-SNE) technique.

Anchor explanations

The coverage of 100% in anchor explanations is considered plausible, but Tables S6 and S7 show it is generally rare (mainly for 6-mer motifs, but less rare for 3-mer motifs) and often indicates a trivial or highly simplistic explanation rather than a universally applicable rule. In the original formulation of Anchors explanation method, coverage is defined with respect to a conditional distribution over perturbations given the anchor. Therefore, 100% coverage does not merely indicate “rarity” or “simplicity,” but rather reflects a degenerate condition in the sampling distribution or in the learned decision boundary. In practice, this may often signal a methodological issue (either in the perturbation generator or in the model’s local decision landscape) rather than a meaningful explanation. It is important to explicitly acknowledge this as a potential artifact rather than framing it as simply “plausible.” In the context of Anchors (model-agnostic, high-precision, rule-based explanations), 100% coverage means the anchor applies to all samples in the perturbation space. Here is why 100% coverage may be plausible yet specific:

1) Empty Anchors (Global Rules): An anchor with 100% coverage often indicates an "empty" rule (i.e., no features are required to maintain the prediction), which happens when a prediction is extremely robust across all perturbations. However, it is worthy to comment about the notion of “empty anchors” is introduced but not rigorously defined. While it is true that an anchor with no predicates can yield maximal coverage, the interpretation that this corresponds to “extremely robust predictions” may be misleading. In most realistic settings, an empty anchor implies that the model prediction is invariant under the perturbation distribution, which may stem from poorly designed perturbations (e.g., sampling outside the data manifold) rather than genuine robustness. This distinction is critical, especially in domains like peptide classification where the input space is highly structured and discrete. Without constraining perturbations to biologically valid sequences, 100% coverage becomes sometimes meaningless.

2) Imbalanced Data: If a model predicts one class almost all the time (due to high imbalance, not the case here), an anchor can easily satisfy the 100% coverage criteria, as most random perturbations will still result in the same prediction. It is noteworthy to add that

high imbalance can indeed bias anchor explanations toward trivial rules; however, the key issue is not just prediction frequency but the interaction between the model's decision boundary and the perturbation distribution. Even in balanced datasets, a poorly calibrated model or overly smooth decision function (e.g., induced by embeddings from large models like ESM-2) can produce artificially high coverage anchors. So, it is important to expand this point to include model calibration and representation effects, not just dataset imbalance.

3) Trade-off with Specificity: Rather than a simple trade-off between coverage and specificity, anchor explanations are constructed by first ensuring that a rule satisfies a strict precision threshold, and only then expanding its coverage as much as possible. Highly specific rules tend to emerge when broader conditions fail to meet the required precision, thereby limiting coverage. Conversely, high coverage (approaching 100%) with maintained precision typically indicates that the precision constraint is trivially satisfied across the perturbation space, which may reflect a simplified local decision boundary or inadequacies in the perturbation distribution rather than a genuinely generalizable explanation.

4) Definition of Scope: An anchor with 100% coverage still only applies to the local neighborhood or perturbation space defined for that specific instance, not necessarily the entire global dataset. This neighborhood is implicitly induced by the perturbation distribution, which is often heuristic and may not reflect the true data manifold. In structured biological domains (e.g., peptide sequences), this is a major limitation: perturbations may generate invalid or non-physical sequences, making coverage estimates unreliable.

While 100% is technically possible, anchor explanations usually aim to find the highest possible coverage subject to a high precision threshold, often leading to coverage values lower than 100% in realistic, complex scenarios. However, 100% coverage may be a red flag in practice, warranting investigation into the perturbation strategy, the fidelity of the local surrogate explanation, and the possibility of model collapse or over-regularization.

In Table S6, the explanations with precision close to 100% and coverage equal to 0% are generally not meaningful or useful, and in some cases, they may indicate a methodological or implementation issue rather than a valid interpretability result. Within the framework of Anchors explanation method, coverage equal to 0% implies that the rule applies to essentially no samples in the perturbation distribution. Even if the precision is close to 100%, this combination is problematic for several reasons. First, an explanation with zero coverage is effectively non-generalizable. It describes a condition that is so specific (or so rarely satisfied) that it does not meaningfully characterize the model's behavior, even locally. In practice, this often corresponds to a rule that only holds for the original instance (or not even reliably under perturbation), making it useless for understanding decision logic. Second, the apparent high precision is typically misleading or ill-defined. Precision in Anchors is estimated over sampled perturbations that satisfy the rule. If coverage is zero (or near-zero), then the number of such samples is either zero or extremely small, meaning the precision estimate is based on insufficient statistical support. In the extreme case, this may arise from sampling artifacts where no perturbed instance satisfies the anchor, rendering the precision estimate meaningless. Third, this situation may often reflect pathological behavior in the explanation process, such as overly restrictive predicates that collapse the rule space, poor or invalid perturbation strategies (especially in structured domains like peptide sequences), or numerical/implementation issues in the sampling procedure.

From a practical standpoint, anchor explanations are intended to provide high-precision rules with non-trivial coverage, that is, rules that reliably explain the prediction over a meaningful region of the input space. A useful explanation typically balances precision (fidelity to the model), and coverage (representativeness of the rule). When coverage collapses to zero, that balance is lost entirely. In summary, explanations with ~100% precision and 0% coverage should be treated as invalid or uninformative, and they warrant careful inspection of the perturbation mechanism, sampling adequacy, and rule construction process rather than interpretation as meaningful insights into the model.

A comparative analysis across the three methods in Table S6 reveals recurring 6-mer patterns that are consistently associated with antioxidant predictions. Among the most prominent motifs are:

- Hydrophobic/aromatic-rich segments, which are enriched in phenylalanine (F), tryptophan (W), and leucine (L). These residues are well-known to contribute to radical scavenging through electron donation and stabilization of reactive species.
- Positively charged motifs, which contain lysine (K) and arginine (R). These residues are frequently implicated in antioxidant peptides due to their ability to interact with negatively charged radicals or metal ions.
- Cysteine-containing fragments, which suggest potential roles in redox activity via thiol chemistry.
- Mixed polar-hydrophobic regions, which may contribute to both solubility and interaction with reactive oxygen species.

Table S6 suggests that the most likely 6-mers influencing antioxidant activity are those enriched in aromatic residues (F, W), positively charged residues (K, R), and redox-active cysteine motifs, often arranged in short contiguous segments. The convergence of LIME and SHAP on these motifs provides strong evidence for their relevance, while the limited utility of Anchor explanations underscores the need for careful selection of interpretability methods in protein sequence modeling. For future work, integrating these insights with physicochemical descriptors and structural analysis could further validate the functional role of these motifs and enhance the interpretability of ESM-based models in peptide bioactivity prediction.

A cross-method comparison in Table S7 reveals several recurrent 6-mer classes that are strongly associated with antioxidant activity:

1. Aromatic and hydrophobic-rich motifs, which are consistently identified. These are enriched in phenylalanine (F), tryptophan (W), tyrosine (Y), and leucine (L), residues known to contribute to antioxidant activity via electron donation and radical stabilization. The repeated identification of these motifs across LIME and SHAP strongly supports their functional relevance.
2. Positively charged and amphipathic segments, which are enriched of lysine (K) and arginine (R)-rich regions. These residues are associated with electrostatic interactions and metal ion chelation, both of which are critical in antioxidant mechanisms. The frequent appearance of these motifs suggests that charge distribution is a key determinant captured by the model.
3. Cysteine-containing and redox-active motifs, which contain the presence of cysteine (C), which plays a central role in thiol-mediated redox reactions. The recurrence of these motifs across multiple sequences and methods provides strong biochemical support for their involvement in antioxidant function.
4. Glycine- and flexible linker-rich regions, which suggest structural flexibility and may facilitate conformational adaptability and interaction with reactive species. These motifs are particularly prominent in LIME explanations with high influence scores.

Importantly, there is a high degree of overlap between LIME and SHAP explanations, often identifying nearly identical or shifted 6-mers (e.g., *FLPGLI*, *PGLIKA*, *GLIKAA*), reflecting the sliding-window nature of 6-mer tokenization. This redundancy is not a limitation but rather an indication that the model's decision function is locally smooth in sequence space, with contiguous residues contributing collectively to the prediction. In contrast, Anchor explanations tend to select a subset of these motifs but fail to capture their distributed contribution.

A critical insight emerges when examining motifs that appear across all three methods. For example, motifs such as *FLPGLI*, *GWANTL*, *RFIYMK*, *SVLCAV*, and *WRKPRW* are consistently identified, suggesting that they represent high-confidence determinants

of antioxidant activity. These motifs combine hydrophobic, aromatic, and charged residues, aligning well with known structure–activity relationships in antioxidant peptides. However, it is equally important to note that SHAP values are often of small magnitude (~10^{-3}), which, while expected, complicates direct interpretation of effect size. Additionally, the presence of negative contributions for certain k-mers in LIME (e.g., *SMKLSF*, *DSCRKQ*) highlights the context-dependent nature of sequence features, where the same residue composition may contribute differently depending on its position and surrounding context.

Table S7 demonstrates that antioxidant activity in peptides is encoded as a distributed, multi-feature signal, rather than being driven by single deterministic motifs. The most likely k-mers influencing antioxidant properties are those enriched in aromatic residues (F, W, Y), positively charged residues (K, R), cysteine (C), and flexible glycine-rich segments, often appearing in short contiguous regions. The strong agreement between LIME and SHAP supports the robustness of these findings, while the limitations of Anchor explanations highlight the challenges of rule-based interpretability in high-dimensional biological sequence spaces. These results underscore the importance of combining multiple interpretability approaches to obtain a reliable and biologically meaningful understanding of deep learning models applied to peptide classification.

Table S6. Performance of the model for the testing dataset (which was separated before splitting) after optimization of the deep learning model (TCN + LSTM) to classify antioxidant peptides using an Evolutionary Scale Modeling (ESM) language model (esm2_t6_8M_UR50D) to capture both global semantic and local structural features. The model was tested using a testing dataset with the original dataset was split to yield 10 instances (10 antioxidant peptides and 10 non-antioxidant peptides). Anchor, LIME, and SHAP explanations are presented for each correct prediction of antioxidant peptides.

Dataset	Sequence	Anchor		LIME		SHAP	
		k-mers	Prediction	k-mers	Influence	k-mers	Influence
1	VQYPVEHPNKFLKFGMTPSKGVLFY	VQYPVE, TPSKGV, KFLKFG	Precision: 0.975 Coverage: 1.0	QYPVEH FLKFGM KGVLFY	0.0557 0.0424 0.0291	PNKFLK VQYPVE HPNKFL PVEHPN KGVLFY	0.0011 -0.0011 0.0009 0.0006 -0.0005
1	AKAWGIPPHVIPQIVPVRIRPLCGNV	AKAWGI PQIVPV	Precision: 0.961 Coverage: 0.0	WGIPPH RIRPLC PVRIRP KAWGIP AKAWGI AWGIPP	0.0824 0.0719 0.0647 0.0630 0.0494 0.0324	AKAWGI PHVIPQ KAWGIP PPHVIP IVPVRI	-0.0009 0.0008 -0.0006 0.0005 -0.0004

				IRPLCG<br>IPPHVI<br>VPVRIR<br>IVPVRI	0.0269<br>0.0230<br>0.0176<br>-0.0167		
1	GFWDSVKEGLKNAAVTILNKIKCKISECPPA	GFWDSV<br>DSVKEG<br>LKNAAV<br>KNAAVT<br>IKCKIS<br>KISECP<br>CKISEC<br>LNKIKC	Precision: 0.994<br>Coverage: 0.0	KNAAVT<br>KISECP<br>CKISEC<br>ISECPP<br>FWDSVK<br>LNKIKC<br>WDSVKE<br>SVKEGL<br>KIKCKI<br>VTILNK	0.1004<br>0.0961<br>0.0825<br>0.0805<br>0.0717<br>0.0698<br>0.0675<br>0.0608<br>-0.0418<br>-0.0335	EGLKNA<br>GFWDSV<br>LKNAAV<br>GLKNAA<br>KEGLKN	0.0008<br>-0.0006<br>0.0003<br>0.0003<br>0.0002
1	FIPGLRRLFATVVPTVVCAINKLPPG	RLFATV<br>LFATVV<br>VVPTVV<br>FIPGLR	Precision: 0.975<br>Coverage: 0.0	VPTVVC<br>GLRRLF<br>VVCAIN<br>VCAINK<br>RRLFAT	0.0740<br>0.0704<br>0.0640<br>0.0628<br>0.0597	GLRRLF<br>VVCAIN<br>PGLRRL	0.0007<br>-0.0005<br>0.0005

				CAINKL<br>PGLRRL<br>INKLPP<br>AINKLP<br>TVVPTV	0.0596<br>0.0580<br>0.0547<br>0.0486<br>-0.0356	LFATVV<br>IPGLRR	0.0004<br>0.0003
1	SIITMTKEAKLPQLWKQIACRLYNTC	TKEAKL<br>LWKQIA<br>KQIACR<br>KLPQLW<br>TMTKEA<br>IACRLY<br>CRLYNT	Precision:<br>0.991<br>Coverage:<br>0.0	IACRLY<br>WKQIAC<br>RLYNTC<br>KLPQLW<br>SIITMT<br>LPQLWK<br>TMTKEA<br>TKEAKL<br>AKLPQL<br>CRLYNT	0.1428<br>0.1287<br>0.1110<br>0.1068<br>0.0958<br>-0.0618<br>0.0490<br>0.0472<br>0.0452<br>0.0331	EAKLPQ<br>TKEAKL<br>TMTKEA<br>MTKEAK<br>KEAKLP	0.0010<br>0.0007<br>0.0005<br>0.0004<br>0.0004
1	FRSGILKLASKIPSVLCAVLKNC	FRSGIL<br>SVLCAV	Precision:<br>0.961	FRSGIL<br>ASKIPS<br>SVLCAV<br>ILKLAS	0.1876<br>0.1150<br>0.1149<br>0.1109	LASKIP<br>ILKLAS<br>KIPSVL	0.0005<br>0.0005<br>-0.0004

		SKIPSV<br>ILKLAS	Coverage:<br>0.0	RSGILK<br>AVLKNC<br>LASKIP<br>IPSVLC<br>PSVLCA<br>KIPSV	0.0957<br>0.0851<br>0.0779<br>0.0708<br>0.0704<br>0.0664	ASKIPS<br>SVLCAV	0.0004<br>-0.0004
1	DDPDKGMLKWKNDFFQEF	DDPDKG<br>KGMLKW<br>NDFFQE<br>DFFQEF<br>LKWKND	Precision:<br>0.958<br>Coverage:<br>0.0	NDFFQE<br>KGMLKW<br>LKWKND<br>DFFQEF<br>WKNDFF<br>DPDKGM<br>KWKNDF<br>KNDFFQ<br>DDPDKG<br>DKGMLK	0.2519<br>0.2449<br>0.1899<br>0.1113<br>0.0747<br>0.0730<br>0.0606<br>0.0426<br>0.0352<br>-0.0307	DPDKGM<br>DKGMLK<br>LKWKND<br>KNDFFQ<br>DFFQEF	0.0007<br>0.0006<br>0.0006<br>-0.0004<br>-0.0004
1	GLLDSVKEGLKKVAGQLLDTLKCKIS<br>GCTPA	GLLDSV<br>EGLKKV<br>LKCKIS	Precision:<br>0.962	GQLLDT<br>KCKISG<br>KKVAGQ	0.2064<br>0.1437<br>0.1388	GLLDSV<br>EGLKKV<br>SVKEGL	-0.0007<br>0.0006<br>-0.0004

		DSVKEG<br>KISGCT	Coverage:<br>0.0	LLDSVK<br>CKISGC<br>GLKKVA<br>ISGCTP<br>LKKVAG<br>GLLDSV<br>TLKCKI	0.1033<br>0.0942<br>0.0892<br>0.0880<br>0.0654<br>-0.0617<br>-0.0412	LDSVKE<br>KEGLKK	-0.0003<br>0.0003
1	GFFDRIKALTKNVTLELLNTITCKLPVTPP	ALTKNV<br>RIKALT<br>TCKLPV<br>KNVTLE<br>LELLNT<br>TKNVTL	Precision:<br>0.983<br>Coverage:<br>0.0	LNTITC<br>LLNTIT<br>RIKALT<br>GFFDRI<br>ALTKNV<br>TITCKL<br>KNVTLE<br>LTKNVT<br>TKNVTL<br>LELLNT	0.1064<br>0.1037<br>0.1004<br>0.0932<br>0.0910<br>0.0904<br>0.0779<br>0.0773<br>0.0742<br>0.0646	DRIKAL<br>FDRIKA<br>KNVTLE<br>TKNVTL<br>IKALTK	0.0014<br>0.0006<br>-0.0005<br>-0.0004<br>-0.0003

1	SFGAKNAVKNGLQKLRNQCQANNY QGPFCDIFKKNP	GAKNAV<br>RNQCQA<br>CQANNY<br>QGPFCD<br>GLQKLR<br>NYQGPF	Precision:<br>0.992<br>Coverage:<br>0.0	PFCDIF<br>CQANNY<br>GLQKLR<br>KLRNQC<br>GAKNAV<br>AVKNGL<br>YQGPFC<br>NYQGPF<br>NAVKNG<br>SFGAKN	0.1340<br>0.1135<br>0.0999<br>0.0912<br>0.0751<br>0.0660<br>0.0659<br>0.0645<br>0.0485<br>-0.0476	GAKNAV<br>FGAKNA<br>VKNGLQ<br>KNAVKN<br>AVKNGL	0.0007<br>0.0006<br>0.0006<br>-0.0006<br>0.0004

Table S7. Performance of the model for the testing dataset (which was separated before splitting) after optimization of the deep learning model (TCN + LSTM) to classify antioxidant peptides using an Evolutionary Scale Modeling (ESM) language model (esm2_t6_8M_UR50D) to capture both global semantic and local structural features. The model was tested using a testing dataset with 31 antioxidant peptides from the APD6 database (Wang, G., Schmidt, C., Li, X. and Wang, Z. (2026) APD6: The antimicrobial peptide database is expanded to promote research and development by deploying an unprecedented information pipeline. *Nucleic Acids Research 54*, D363-D374.) Anchor, LIME, and SHAP explanations are presented for each correct prediction of antioxidant peptides.

Dataset	Sequence	Anchor		LIME		SHAP	
		k-mers	Prediction	k-mers	Influence	k-mers	Influence
2	SSMKLSFRARAYGFRGPGPQL	LSFRAR<br>MKLSFR<br>RARAYG<br>FRARAY<br>GFRGPG	Precision:<br>1.0<br>Coverage:<br>0.0	RARAYG<br>RAYGFR<br>SMKLSF<br>AYGFRG<br>LSFRAR<br>GFRGPG<br>MKLSFR<br>GPGPQL	0.1253<br>0.1172<br>-0.0986<br>0.0905<br>0.0886<br>0.0878<br>0.0713<br>0.0680	AYGFRG<br>YGFRGP<br>GPGPQL<br>KLSFRA<br>SSMKLS	0.0050<br>0.0038<br>0.0030<br>0.0029<br>0.0027

				FRGPGP<br>RGPGPQ	0.0653<br>-0.0589		
2	AELRCMCIKTTSGIHPKNIQSLEVIGKGTHCNQVEVIATLKDGRKICLDPDAPRIKKIVQKKLAGDES	CNQVEV<br>AELRCM<br>VQKKLA<br>EVIATL<br>CLDPDA<br>IVQKKL<br>RIKKIV<br>KNIQSL<br>ELRCMC<br>GRKICL<br>TLKDGR<br>VIGKGT<br>PDAPRI<br>ATLKDG<br>DGRKIC<br>TTSGIH<br>SLEVIG	Precision:<br>0.976<br>Coverage:<br>0.0	ELRCMC<br>ATLKDG<br>LRCMCI<br>EVIATL<br>GKGTHC<br>DGRKIC<br>IATLKD<br>APRIKK<br>NQVEVI<br>GTHCNQ	0.0467<br>0.0317<br>0.0261<br>0.0238<br>0.0229<br>0.0226<br>0.0225<br>-0.0175<br>-0.0171<br>-0.0168	HPKNIQ<br>TSGIHP<br>IHPKNI<br>ELRCMC<br>SGIHPK	0.0068<br>0.0054<br>0.0052<br>0.0051<br>0.0049

		QVEVIA<br>ICLDPD<br>MCIKTT					
2	GWANTLKNVAGGLCKITGAA	GWANTL<br>TLKNVA<br>NVAGGL<br>GLCKIT	Precision:<br>1.0<br>Coverage:<br>0.0	WANTLK<br>VAGGLC<br>GWANTL<br>LCKITG<br>GLCKIT<br>KNVAGG<br>TLKNVA<br>LKNVAG<br>GGLCKI<br>AGGLCK	0.0700<br>0.0636<br>0.0634<br>0.0630<br>0.0368<br>0.0346<br>0.0336<br>0.0300<br>0.0243<br>-0.0195	NTLKNV<br>ANTLKN<br>TLKNVA<br>LKNVAG<br>WANTLK	0.0077<br>0.0075<br>0.0060<br>0.0056<br>0.0046
2	RFIYMKGFGKPRFGKR	RFIYMK<br>GKPRFG<br>GFGKPR<br>FIYMKG	Precision:<br>1.0<br>Coverage:<br>0.0	GFGKPR<br>GKPRFG<br>YMKGFG<br>PRFGKR<br>RFIYMK<br>MKGFGK	0.0564<br>0.0437<br>0.0421<br>0.0395<br>0.0380<br>-0.0342	FIYMKG<br>IYMKGF<br>RFIYMK<br>YMKGFG<br>PRFGKR	0.0063<br>0.0058<br>0.0054<br>0.0040<br>0.0036

				KGFGKP<br>KPRFGK<br>FIYMKG<br>IYMKGF	0.0300<br>-0.0265<br>0.0237<br>0.0186		
2	CARLNCVPKGTSGNTETCPCYASLHSCRKYG	CARLNC<br>ETCPCY<br>GNTETC<br>NCVPKG<br>HSCRKY	Precision:<br>0.958<br>Coverage:<br>0.0	CARLNC<br>ASLHSC<br>SLHSCR<br>GNTETC<br>TETCPC<br>HSCRKY<br>ARLNCV<br>CYASLH<br>TCPCYA<br>LHSCRK	0.0116<br>0.0101<br>0.0101<br>0.0099<br>0.0098<br>0.0097<br>0.0095<br>0.0093<br>0.0083<br>-0.0082	CARLNC<br>NTETCP<br>ARLNCV<br>CYASLH<br>YASLHS	0.0050<br>0.0046<br>0.0046<br>0.0044<br>0.0043
2	RILTMTKRVKMPQLYKQIVCRLFKTC	KMPQLY<br>RILTMT<br>LYKQIV<br>TKRVKM<br>QIVCRL	Precision:<br>0.954<br>Coverage:<br>0.0	KMPQLY<br>TKRVKM<br>LYKQIV<br>YKQIVC<br>TMTKRV	0.1039<br>0.0716<br>0.0537<br>0.0519<br>0.0493	LYKQIV<br>YKQIVC<br>QIVCRL	0.0070<br>0.0054<br>0.0050

		TMTKRV<br>CRLFKT		RLFKTC<br>VKMPQL<br>MTKRVK<br>CRLFKT<br>IVCRLF	0.0437<br>0.0400<br>-0.0375<br>0.0372<br>-0.0241	IVCRLF<br>QLYKQI	0.0044<br>0.0043
2	FLSGILKLAFKIPSVLCAVLKNC	SVLCAV<br>FLSGIL<br>FKIPSV<br>KIPSVL<br>PSVLCA<br>CAVLKN<br>GILKLA<br>AFKIPS<br>VLCAVL	Precision:<br>1.0<br>Coverage:<br>0.0	FKIPSV<br>SVLCAV<br>PSVLCA<br>AFKIPS<br>CAVLKN<br>IPSVLC<br>AVLKNC<br>FLSGIL<br>LAFKIP<br>VLCAVL	0.2687<br>0.2394<br>0.1894<br>0.1538<br>0.1364<br>0.1288<br>0.0990<br>0.0781<br>0.0751<br>0.0726	SVLCAV<br>PSVLCA<br>VLCAVL<br>IPSVLC<br>AVLKNC	0.0073<br>0.0069<br>0.0067<br>0.0060<br>0.0056
2	SKWQHQQDSCRKQLQGVNLTPCEKHI<br>MEKIQGRGDDDDDDDDD	CEKHIM<br>NLTPCE<br>QGVNLT<br>EKHIME	Precision:<br>0.985<br>Coverage:	HQQDSC<br>EKHIME<br>CEKHIM<br>NLTPCE	0.3095<br>0.2320<br>0.2136<br>0.2115	LQGVNL<br>HIMEKI<br>KHIMEK	0.0042<br>0.0040<br>0.0036

		LQGVNL<br>QQDSCR<br>EKIQGR<br>SCRKQL<br>HQQDSC<br>HIMEKI<br>SKWQHQ<br>WQHQQD<br>GVNLTP	0.0	VNLTPC<br>CRKQLQ<br>IMEKIQ<br>DSCRKQ<br>DDDDDD<br>QHQQDS	0.1480<br>-0.1444<br>-0.1429<br>-0.1380<br>-0.1181<br>-0.1095	EKHIME<br>QLQGVN	0.0036<br>0.0034
2	SDYLNNNPLFPRYDIGNVELSTAYRSFA<br>NQKAPGRLNQNWALTADYTYR	ELSTAY<br>PLFPRY<br>TADYTY<br>NQKAPG<br>AYRSFA<br>SDYLNN<br>YLNNNP<br>VELSTA	Precision:<br>0.965<br>Coverage:<br>0.0	ELSTAY<br>ALTADY<br>FPRYDI<br>LNQNWA<br>FANQKA<br>TAYRSF<br>QKAPGR<br>RSFANQ<br>APGRLN<br>PGRLNQ	0.0538<br>0.0516<br>0.0467<br>0.0452<br>0.0451<br>0.0446<br>0.0421<br>-0.0300<br>-0.0207<br>-0.0129	DYLNNN<br>YLNNNP<br>LNNNPL<br>SDYLNN<br>NNPLFP	0.0087<br>0.0085<br>0.0075<br>0.0066<br>0.0051

2	FLPVLGKVIKLVGGLL		No Antioxidant				
2	FLPGLIKAAVGVGSTILCKITKKC	FLPGLI<br>TILCKI<br>VGVGST<br>AAVGVG<br>VGSTIL<br>GLIKAA<br>KITKKC<br>IKAAVG<br>GVGSTI	Precision:<br>0.988<br>Coverage:<br>0.0	VGVGST<br>FLPGLI<br>VGSTIL<br>ILCKIT<br>AVGVGS<br>TILCKI<br>PGLIKA<br>LPGLIK<br>GVGSTI<br>LCKITK	0.2357<br>0.1899<br>0.1682<br>0.1407<br>0.1186<br>0.1069<br>0.1045<br>0.1004<br>0.0767<br>-0.0573	VGVGST<br>STILCK<br>GVGSTI<br>GSTILC<br>VGSTIL	0.0061<br>0.0060<br>0.0058<br>0.0057<br>0.0055
2	FLPGLIKAAVGIGSTIFCKISKKC	FLPGLI<br>KISKKC<br>TIFCKI<br>IGSTIF<br>AAVGIG<br>GLIKAA<br>VGIGST	Precision:<br>1.0<br>Coverage:<br>0.0	FLPGLI<br>VGIGST<br>TIFCKI<br>IGSTIF<br>AAVGIG<br>AVGIGS<br>IFCKIS	0.2393<br>0.2093<br>0.1457<br>0.1265<br>0.1183<br>0.1058<br>0.0993	STIFCK<br>GSTIFC<br>IGSTIF<br>TIFCKI<br>GIGSTI	0.0072<br>0.0071<br>0.0057<br>0.0055<br>0.0055

		IKAAVG<br>LIKAAV<br>GSTIFC		PGLIKA<br>KISKKC<br>STIFCK	0.0990<br>0.0919<br>-0.0513		
2	FLPGLIKVAVGVGSTILCKITKKC	GVGSTI<br>FLPGLI<br>ILCKIT<br>TILCKI<br>VGVGST<br>VAVGVG<br>GLIKVA<br>IKVAVG<br>VGSTIL	Precision:<br>0.976<br>Coverage:<br>0.0	VGVGST<br>FLPGLI<br>ILCKIT<br>GVGSTI<br>VGSTIL<br>KITKKC<br>LIKVAV<br>LCKITK<br>TILCKI<br>CKITKK	0.2708<br>0.2049<br>0.1922<br>0.1537<br>0.1321<br>0.1101<br>0.1048<br>-0.1033<br>0.0870<br>-0.0643	VGVGST<br>GVGSTI<br>STILCK<br>GSTILC<br>VGSTIL	0.0070<br>0.0062<br>0.0060<br>0.0059<br>0.0056
2	FLPMLAGLAANFLPKIICKITKKC	FLPMLA<br>LAANFL<br>MLAGLA<br>KIICKI<br>LAGLAA<br>KITKKC	Precision:<br>0.995<br>Coverage:<br>0.0	LPMLAG<br>LAGLAA<br>IICKIT<br>MLAGLA<br>AANFLP<br>LAANFL	0.2006<br>0.1551<br>0.1411<br>0.1344<br>0.1013<br>0.0716	PMLAGL<br>LPMLAG<br>MLAGLA<br>FLPMLA<br>LAGLAA	0.0076<br>0.0071<br>0.0067<br>0.0061<br>0.0054

		GLAANF<br>LPMLAG<br>LPKIIC		KIICKI<br>ANFLPK<br>ICKITK<br>CKITKK	0.0690<br>-0.0614<br>-0.0604<br>-0.0486		
2	FFSLIPSLVGGLISAFK		No Antioxidant				
2	FLTGLIGGLMKALGK	FLTGLI<br>LTGLIG<br>LMKALG<br>GLIGGL<br>LIGGLM<br>GGLMKA	Precision:<br>1.0<br>Coverage:<br>0.0	LTGLIG<br>TGLIGG<br>GLIGGL<br>LMKALG<br>LIGGLM<br>MKALGK<br>FLTGLI<br>IGGLMK<br>GGLMKA<br>GLMKAL	0.3620<br>0.2935<br>0.2102<br>0.1562<br>0.0840<br>-0.0828<br>0.0734<br>-0.0646<br>-0.0316<br>-0.0135	LTGLIG<br>FLTGLI<br>TGLIGG<br>GLIGGL<br>LIGGLM	0.0054<br>0.0052<br>0.0050<br>0.0042<br>0.0021
2	GAPKGCWTKSYPPQPCFGKK	GAPKGC<br>APKGCW<br>CWTKSY	Precision:<br>0.964	CWTKSY<br>APKGCW<br>PPQPCF	0.1856<br>0.1844<br>0.0884	YPPQPC<br>QPCFGK<br>WTKSYP	0.0047<br>0.0032<br>0.0029

		YPPQPC<br>PKGCWT<br>SYPPQP	Coverage:<br>0.0	GAPKGC<br>PQPCFG<br>PKGCWT<br>QPCFGK<br>KSYPPQ<br>YPPQPC<br>PCFGKK	-0.0810<br>0.0644<br>0.0623<br>-0.0607<br>0.0606<br>0.0596<br>-0.0560	PCFGKK<br>SYPPQP	0.0027<br>0.0025
2	CVVSSGWKWNYKIRCKLTGNC	CVVSSG<br>GWKWNY<br>NYKIRC<br>SSGWKW<br>RCKLTG<br>KIRCKL	Precision:<br>1.0<br>Coverage:<br>0.0	GWKWNY<br>VVSSGW<br>IRCKLT<br>YKIRCK<br>KIRCKL<br>SSGWKW<br>NYKIRC<br>VSSGWK<br>WKWNYK<br>CKLTGN	0.0493<br>0.0353<br>0.0301<br>-0.0269<br>0.0264<br>0.0229<br>0.0133<br>0.0130<br>-0.0092<br>0.0082	CVVSSG<br>VVSSGW<br>VSSGWK<br>KLTGNC<br>CKLTGN	0.0071<br>0.0065<br>0.0054<br>0.0050<br>0.0049

2	SMATPHVAGAAALILSKHPTWTNAQVR DRLESTATYLGNSFYYGK	ESTATY<br>LILSKH<br>NSFYYG<br>LGNSFY<br>SKHPTW<br>TATYLG	Precision:<br>0.967<br>Coverage:<br>0.0	SFYYGK<br>GNSFYY<br>LGNSFY<br>STATYL<br>AGAAAL<br>WTNAQV<br>TWTNAQ<br>MATPHV<br>HVAGAA	0.0278<br>0.0234<br>0.0207<br>0.0185<br>0.0177<br>-0.0157<br>-0.0139<br>-0.0120<br>-0.0095	ATPHVA<br>ATYLGN<br>TPHVAG<br>AGAAAL<br>HVAGAA	0.0054<br>0.0050<br>0.0049<br>0.0048<br>0.0047
2	GLFSKKGGKGGKSWIKGVFKGIKGIGK EVGGDVIRTGIEIAACKIKGEC	GIKGIG<br>LFSKKG<br>DVIRTG<br>FSKKGG<br>KGGKGG<br>GIGKEV<br>EVGGDV<br>GGDVIR<br>KKGGKG<br>IGKEVG	Precision:<br>0.969<br>Coverage:<br>0.0	KGGKSW<br>FKGIKG<br>WIKGVF<br>KGGKGG<br>KSWIKG<br>IRTGIE<br>KKGGKG<br>IGKEVG<br>GKSWIK<br>GKGGK	0.0963<br>0.0878<br>0.0820<br>0.0805<br>0.0797<br>0.0733<br>0.0690<br>0.0676<br>-0.0534<br>-0.0516	FSKKGG<br>SKKGGK<br>LFSKKG<br>GKGGKS<br>GGKGGK	0.0043<br>0.0041<br>0.0040<br>0.0032<br>0.0032

		SWIKGV<br>KGVFKG<br>GLFSKK<br>FKGIKG					
2	FLPFFAACAITRKC		No Antioxidant				
2	GLISTIWNTASNVAGTLTDSVKCKFKKC	SVKCKF<br>TLTDSV<br>TIWNTA<br>NVAGTL<br>TASNVA<br>LISTIW<br>ISTIWN<br>GLISTI	Precision:<br>0.966<br>Coverage:<br>0.0	CKFKKC<br>TASNVA<br>STIWNT<br>SNVAGT<br>VAGTLT<br>VKCKFK<br>NTASNV<br>NVAGTL<br>DSVKCK<br>KCKFKK	0.1121<br>0.0852<br>0.0820<br>0.0814<br>0.0631<br>-0.0610<br>0.0585<br>0.0535<br>-0.0529<br>-0.0469	STIWNT<br>ISTIWN<br>NVAGTL<br>LISTIW<br>AGTLTD	0.0068<br>0.0065<br>0.0060<br>0.0053<br>0.0051
2	SLFSLIKAGAKFLGKNMLKQGPQYPAC<br>KVSKDSENVNWKS	NMLKQG<br>AGAKFL<br>KDSENV	Precision:<br>0.962	AGAKFL<br>LIKAGA<br>FSLIKA	0.1638<br>0.1463<br>0.1417	GKNMLK<br>LGKNML<br>KNMLKQ	0.0064<br>0.0059<br>0.0052

		LGKNML<br>KQGPQY<br>FSLIKA<br>LIKAGA<br>FLGKNM<br>YPACKV<br>SLIKAG<br>GAKFLG<br>SENVNW<br>PACKVS<br>CKVSKD	Coverage:<br>0.0	SLIKAG<br>SLFSLI<br>MLKQGP<br>SENVNW<br>GAKFLG<br>KQGPQY<br>LGKNML	0.1313<br>-0.1268<br>0.1196<br>0.1134<br>0.1133<br>0.1070<br>0.1017	FLGKNM<br>AGAKFL	0.0042<br>0.0042
2	KKCNFFCKLKKKVKSVGSRNLIGSATH<br>HHRIYRV	NFFCKL<br>KLKKKV<br>HHHRIY<br>KVKSVG<br>KSVGSR<br>VGSRNL<br>IGSATH<br>ATHHHR	Precision:<br>0.987<br>Coverage:<br>0.0	KLKKKV<br>ATHHHR<br>KVKSVG<br>KSVGSR<br>LKKKVK<br>KKVKSV<br>HHRIYR<br>SRNLIG	0.1122<br>0.1107<br>0.0941<br>0.0847<br>-0.0827<br>0.0784<br>0.0764<br>0.0754	VKSVGS<br>KVKSVG<br>VGSRNL<br>KSVGSR<br>SVGSRN	0.0094<br>0.0082<br>0.0077<br>0.0069<br>0.0063

				HHHRIY<br>FFCKLK	0.0744<br>-0.0666		
2	EGCNILCLLKRKVKAVKNVVKNVVKSV<br>VG	AVKNVV<br>LLKRKV<br>RKVKAV<br>VVKNVV<br>VVKSVV	Precision:<br>0.969<br>Coverage:<br>0.0	RKVKAV<br>LLKRKV<br>NVVKNV<br>KAVKNV<br>AVKNVV<br>VVKNVV<br>LCLLKR<br>LKRKVK<br>NVVKSV<br>VVKSVV	0.1389<br>0.1132<br>0.1024<br>0.1018<br>0.0894<br>0.0867<br>0.0836<br>-0.0789<br>0.0738<br>0.0441	KVKAVK<br>VKAVKN<br>KAVKNV<br>RKVKAV<br>AVKNVV	0.0064<br>0.0062<br>0.0062<br>0.0061<br>0.0058
2	MTPLWRIMGTKPYGAYCQNNYECSTG<br>VCRKGHCSFSQPLNS	MTPLWR<br>YCQNNY<br>KPYGAY<br>MGTKPY<br>LWRIMG<br>GVCRKG	Precision:<br>0.989<br>Coverage:<br>0.0	CRKGHC<br>CSFSQP<br>VCRKGH<br>TGVCRK<br>TKPYGA<br>RIMGTK<br>CSTGVC	0.0150<br>0.0127<br>0.0111<br>-0.0106<br>0.0099<br>-0.0099<br>0.0096	PYGAYC<br>YCQNNY<br>PLWRIM<br>KPYGAY<br>GAYCQN	0.0060<br>0.0057<br>0.0057<br>0.0053<br>0.0047

		ECSTGV<br>STGVCR		STGVCR<br>GVCRKG<br>PLWRIM	0.0087<br>0.0087<br>0.0079		
2	MTPLWRILASKPQGAYCHDHIECSTGLCR	MTPLWR<br>KPQGAY<br>AYCHDH<br>ILASKP<br>STGLCR<br>PQGAYC<br>ASKPQG	Precision:<br>0.980<br>Coverage:<br>0.0	CSTGLC<br>PQGAYC<br>STGLCR<br>LWRILA<br>ECSTGL<br>HDHIEC<br>ASKPQG<br>SKPQGA<br>KPQGAY<br>LASKPQ	0.0203<br>0.0175<br>0.0168<br>0.0162<br>0.0152<br>0.0151<br>0.0145<br>0.0128<br>0.0124<br>-0.0105	AYCHDH<br>GAYCHD<br>YCHDHI<br>PLWRIL<br>LWRILA	0.0064<br>0.0059<br>0.0055<br>0.0054<br>0.0050
2	GGCPDADKCTKYCQKQGISVGKCAKPVNACVCIL	GGCPDA<br>DKCTKY<br>PDADKC<br>KQGISV<br>SVGKCA	Precision:<br>0.954<br>Coverage:<br>0.0	DKCTKY<br>VNACVC<br>KCTKYC<br>PDADKC<br>ISVGKC<br>ADKCTK	0.0430<br>0.0366<br>0.0356<br>0.0323<br>0.0313<br>-0.0254	TKYCQK<br>VGKCAK<br>SVGKCA<br>ISVGKC<br>DKCTKY	0.0039<br>0.0034<br>0.0034<br>0.0031<br>0.0030

		ISVGKC<br>KCAKPV		TKYCQK<br>SVGKCA<br>AKPVNA<br>GKCAKP	-0.0242<br>0.0242<br>0.0209<br>0.0194		
2	GSKKTKCPR		No Antioxidant				
2	RKPRRWRWWPKRP	WWPKRP<br>RKPRRW<br>RWRWWP<br>KPRRWR<br>PRRWRW<br>RWWPKR	Precision:<br>1.0<br>Coverage:<br>0.0	RWRWWP<br>WRWWPK<br>WWPKRP<br>KPRRWR<br>RWWPKR<br>RRWRWW<br>PRRWRW<br>RKPRRW	0.2888<br>-0.2394<br>0.1546<br>0.0553<br>-0.0417<br>0.0286<br>0.0141<br>0.0061	RWWPKR<br>WRWWPK<br>PRRWRW<br>RWRWWP<br>KPRRWR	-0.0016<br>-0.0015<br>0.0013<br>-0.0013<br>0.0009
2	IRPWRKPRWPWKR	WRKPRW<br>RKPRWP<br>RPWRKP<br>KPRWPW	Precision:<br>1.0<br>Coverage:<br>0.0	WRKPRW<br>PRWPWK<br>RKPRWP<br>RPWRKP<br>PWRKPR	0.2863<br>-0.2611<br>0.1673<br>0.1490<br>0.1318	RPWRKP<br>PWRKPR<br>WRKPRW<br>KPRWPW<br>RWPWKR	0.0027<br>0.0022<br>0.0019<br>0.0015<br>-0.0011

		IRPWRK<br>RWPWKR		IRPWRK<br>KPRWPW<br>RWPWKR	-0.0920<br>0.0696<br>0.0416		

Table S8. Traditional model performances using the counting k-mers method in the original dataset.

Model	Accuracy	Precision	Recall	F1	MCC	Kappa
RandomForest	0.902	0.900	0.978	0.938	0.728	0.714
ExtraTrees	0.886	0.898	0.957	0.926	0.681	0.674
Support Vector	0.848	0.877	0.928	0.901	0.574	0.569
Gradient Boosting	0.886	0.927	0.920	0.924	0.698	0.698
KNeighbors	0.826	0.896	0.87	0.882	0.55	0.549
XGBoost	0.908	0.923	0.957	0.94	0.746	0.744

Table S9. Traditional model performances using the counting k-mers method in the augmented dataset.

Model	Accuracy	Precision	Recall	F1	MCC	Kappa
RandomForest	0.981	0.98	0.981	0.98	0.962	0.962
ExtraTrees	0.982	0.981	0.983	0.982	0.964	0.964
Support Vector	0.970	0.974	0.965	0.969	0.940	0.940
Gradient Boosting	0.912	0.917	0.901	0.909	0.823	0.823
KNeighbors	0.962	0.975	0.946	0.96	0.923	0.923
XGBoost	0.980	0.985	0.973	0.979	0.959	0.959

Table S10. Traditional model performances using the one-hot encoding method in the original dataset.

Model	Accuracy	Precision	Recall	F1	MCC	Kappa
RandomForest	0.886	0.903	0.949	0.926	0.683	0.679
ExtraTrees	0.880	0.903	0.942	0.922	0.669	0.667
Support Vector	0.853	0.888	0.920	0.904	0.596	0.594
Gradient Boosting	0.897	0.934	0.928	0.931	0.727	0.727
KNeighbors	0.821	0.872	0.891	0.882	0.512	0.511
XGBoost	0.908	0.923	0.957	0.940	0.746	0.744

Table S11. Traditional model performances using the one-hot encoding method in the augmented dataset.

Model	Accuracy	Precision	Recall	F1	MCC	Kappa
RandomForest	0.956	0.954	0.958	0.956	0.913	0.913
ExtraTrees	0.959	0.958	0.958	0.958	0.918	0.918
Support Vector	0.950	0.949	0.948	0.949	0.900	0.900
Gradient Boosting	0.882	0.876	0.885	0.88	0.764	0.764
KNeighbors	0.943	0.944	0.94	0.942	0.886	0.886
XGBoost	0.962	0.962	0.96	0.961	0.923	0.923

Table S12. LIME Explanations of the random forest model trained with the augmented dataset using k-mers for the independent test dataset 1. Note that all predicted labels are different than the true labels, indicating the random forest model does not offer a reasonable generalization.

sequence	true_label	predicted_label	K=1	K=2	K=3	K=4	K=5	K=6	K=7	K=8	K=9	K=10
VQYPVEHPNKFLKFGMTPSKGVLFY	1	Non-FRS	QYP	PSK	MTP	VEH	VQY	SKG	YPV	KGV	FGM	GMT
AKAWGIPPHVIPQIVPVRIRPLCGNV	1	Non-FRS	CGN	VIP	PPH	RPL	VRI	KAW	IVP	GNV	PHV	GIP
GFWDSVKEGLKNAAVTILNKIKCKISECPPA	1	Non-FRS	ISE	PPA	GLK	EGL	FWD	IKC	CPP	GFW	KCK	VTI
FIPGLRRLFATVVPTVVCAINKLPPG	1	Non-FRS	VCA	NKL	LRR	TVV	INK	LPP	PGL	VVP	AIN	IPG
SIITMTKEAKLPQLWKQIACRLYNTC	1	Non-FRS	CRL	TKE	LYN	YNT	PQL	NTC	IIT	TMT	WKQ	EAK
FRSGILKLASKIPSVLCAVLKNC	1	Non-FRS	LCA	FRS	CAV	IPS	ILK	KIP	ASK	SKI	PSV	GIL
DDPDKGMLKWKNDFFQEF	1	Non-FRS	NDF	MLK	DKG	QEF	FFQ	FQE	DFF	DDP	PDK	KND
GLLDSVKEGLKKVAGQLLDTLKCKISGCTPA	1	Non-FRS	GLK	EGL	LKK	SVK	KVA	LLD	KIS	SGC	GLL	QLL
GFFDRIKALTKNVTLELLNTITCKLPVTPP	1	Non-FRS	LPV	PVT	KAL	LLN	CKL	ALT	GFF	IKA	FDR	TCK
SFGAKNAVKNGLQKLRNQCQANNYQGPFCDIFKKNP	1	Non-FRS	NGL	ANN	LQK	NAV	QGP	IFK	KKN	YQG	KNA	QCQ

Table S13. LIME Explanations of the random forest model trained with the augmented dataset using k-mers for the independent test dataset 2. Note that only 5 predicted labels from 31 samples (16%) are correct comparted with the true labels, indicating the random forest model does not offer a reasonable generalization as well.

sequence	true_label	predicted_label	K=1	K=2	K=3	K=4	K=5	K=6	K=7	K=8	K=9	K=10
SSMKLSFRARAYGFRGPGPQL	1	Non-FRS	GPG	FRG	KLS	LSF	GFR	RGP	AYG	RAY	YGF	PGP
AELRCMCIKTTSGIHPKNIQSLEVIGKGTHCNQVEVIATLKDGRKICLDPDAPRIKKIVQKKLAGDES	1	Non-FRS	IHP	RCM	PDA	TTS	EVI	KKI	QKK	CLD	LAG	QSL
GWANTLKNVAGGLCKITGAA	1	Non-FRS	AGG	ITG	VAG	CKI	WAN	GLC	KIT	TGA	ANT	NVA
RFIYMKGFGKPRFGKR	1	Non-FRS	GKP	FGK	MKG	FIY	PRF	GKR	YMK	KGF	GFG	KPR
CARLNCVPKGTSGNTETCPCYASLHSCRKYG	1	Non-FRS	LHS	GNT	HSC	CYA	NTE	ETC	SGN	GTS	YAS	KGT
RILTMTKRVKMPQLYKQIVCRLFKTC	1	Non-FRS	CRL	RVK	PQL	ILT	RIL	MTK	IVC	KMP	FKT	TMT
FLSGILKLAFKIPSVLCAVLKNC	1	Non-FRS	LCA	CAV	FKI	GIL	IPS	ILK	LSG	VLK	SGI	AVL
SKWQHQQDSCRKQLQGVNLTPCEKHIMEKIQGRGDDDDDDDDD	1	Non-FRS	DDD	MEK	EKH	GVN	LQG	KHI	QGV	EKI	RKQ	QQD
SDYLNNNPLFPRYDIGNVELSTAYRSFANQKAPGRLNQNWALTADYTYR	1	Non-FRS	PGR	PLF	RSF	ADY	NQK	FPR	NNP	YLN	LFP	YTY
FLPVLGKVIKLVGGLL	1	FRS	VGG	LPV	LVG	KVI	VLG	VIK	KLV	GLL	FLP	LGK
FLPGLIKAAVGVGSTILCKITKKC	1	Non-FRS	GST	FLP	VGV	CKI	AAV	KKC	GVG	TIL	IKA	STI
FLPGLIKAAVGIGSTIFCKISKKC	1	Non-FRS	GST	FLP	KIS	IKA	SKK	STI	ISK	IFC	KKC	CKI
FLPGLIKVAVGVGSTILCKITKKC	1	Non-FRS	VAV	GST	FLP	KVA	GVG	CKI	ITK	TIL	LIK	VGS
FLPMLAGLAANFLPKIICKITKKC	1	Non-FRS	LAA	LAG	KII	FLP	MLA	ANF	CKI	TKK	KKC	NFL
FFSLIPSLVGGLISAFK	1	FRS	VGG	LVG	PSL	IPS	SLI	FSL	ISA	AFK	GGL	LIP
FLTGLIGGLMKALGK	1	Non-FRS	LTG	KAL	FLT	LIG	LGK	LMK	TGL	GLM	GLI	ALG
GAPKGCWTKSYPPQPCFGKK	1	FRS	YPP	FGK	WTK	KGC	GKK	TKS	APK	PKG	PQP	KSY
CVVSSGWKWNYKIRCKLTGNC	1	Non-FRS	KLT	TGN	LTG	CKL	NYK	YKI	SGW	WKW	GNC	SSG
SMATPHVAGAAALILSKHPTWTNAQVRDRLESTATYLGNSFYYGK	1	Non-FRS	YLG	MAT	SMA	WTN	KHP	SKH	TAT	HPT	NAQ	EST
GLFSKKGGKGGKSWIKGVFKGIKGIGKEVGGDVIRTGIEIAACKIKGEC	1	FRS	VGG	GGD	GVF	KGG	IKG	GIK	KGI	GKS	GDV	RTG
FLPFFAACAITRKC	1	Non-FRS	PFF	FFA	FLP	ACA	RKC	AAC	LPF	ITR	FAA	CAI

GLISTIWNTASNVAGTLTDSVKCKFKKC	1	Non-FRS	VKC	SVK	KCK	KKC	AGT	ASN	STI	IWN	CKF	TLT
SLFSLIKAGAKFLGKNMLKQGPQYPACKVSKDSENVNWKS	1	FRS	PQY	VSK	PAC	QYP	QGP	LKQ	LGK	KDS	LIK	WKS
KKCNFFCKLKKKVKSVGSRNLIGSATHHHRIYRV	1	Non-FRS	YRV	NLI	KCN	HRI	LIG	LKK	FFC	NFF	RIY	HHR
EGCNILCLLKRKVKAVKNVVKNVVKSVVG	1	Non-FRS	KAV	VVG	NVV	NIL	LLK	VVK	AVK	EGC	LKR	LCL
MTPLWRIMGTKPYGAYCQNNYECSTGVCRKGHCSFSQPLNS	1	Non-FRS	YCQ	MGT	RKG	MTP	YGA	TGV	WRI	SQP	TKP	KGH
MTPLWRILASKPQGAYCHDHIECSTGLCR	1	Non-FRS	HDH	CHD	LWR	PLW	ASK	WRI	LAS	QGA	GLC	MTP
GGCPDADKCTKYCQKQGISVGKCAKPVNACVCIL	1	Non-FRS	PDA	AKP	YCQ	CQK	KCT	TKY	QKQ	VNA	ISV	CPD
GSKKTKCPR	1	Non-FRS	KTK	CPR	GSK	TKC	SKK	KKT	KCP			
RKPRRWRWWPKRP	1	Non-FRS	PRR	WPK	WWP	PKR	WRW	RKP	KRP	RRW	KPR	RWW
IRPWRKPRWPWKR	1	FRS	PWR	PWK	RPW	WPW	RKP	IRP	WRK	WKR	PRW	KPR
